# Oxidative stress enhances the therapeutic action of a respiratory inhibitor in MYC‐driven lymphoma

**DOI:** 10.15252/emmm.202216910

**Published:** 2023-05-09

**Authors:** Giulio Donati, Paola Nicoli, Alessandro Verrecchia, Veronica Vallelonga, Ottavio Croci, Simona Rodighiero, Matteo Audano, Laura Cassina, Aya Ghsein, Giorgio Binelli, Alessandra Boletta, Nico Mitro, Bruno Amati

**Affiliations:** ^1^ European Institute of Oncology (IEO) – IRCCS Milan Italy; ^2^ Center for Genomic Science of IIT@SEMM Milan Italy; ^3^ DiSFeB, Dipartimento di Scienze Farmacologiche e Biomolecolari Università degli Studi di Milano Milan Italy; ^4^ IRCCS San Raffaele Scientific Institute Milan Italy; ^5^ Dipartimento di Biotecnologie e Scienze della Vita Università dell'Insubria Varese Italy

**Keywords:** lymphoma, mitochondria, MYC, ROS, targeted therapy, Cancer, Metabolism

## Abstract

MYC is a key oncogenic driver in multiple tumor types, but concomitantly endows cancer cells with a series of vulnerabilities that provide opportunities for targeted pharmacological intervention. For example, drugs that suppress mitochondrial respiration selectively kill MYC‐overexpressing cells. Here, we unravel the mechanistic basis for this synthetic lethal interaction and exploit it to improve the anticancer effects of the respiratory complex I inhibitor IACS‐010759. In a B‐lymphoid cell line, ectopic MYC activity and treatment with IACS‐010759 added up to induce oxidative stress, with consequent depletion of reduced glutathione and lethal disruption of redox homeostasis. This effect could be enhanced either with inhibitors of NADPH production through the pentose phosphate pathway, or with ascorbate (vitamin C), known to act as a pro‐oxidant at high doses. In these conditions, ascorbate synergized with IACS‐010759 to kill MYC‐overexpressing cells *in vitro* and reinforced its therapeutic action against human B‐cell lymphoma xenografts. Hence, complex I inhibition and high‐dose ascorbate might improve the outcome of patients affected by high‐grade lymphomas and potentially other MYC‐driven cancers.

## Graphical abstract

Deregulated expression of the oncogenic transcription factor MYC in B‐cell lymphoma induces a vulnerability to disruption of redox homeostasis by pharmacological treatment with the electron transport chain (ETC) complex I inhibitor IACS‐010759 and high‐dose ascorbate.

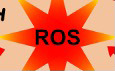

**The paper explained**

**Problem**

The oncogenic transcription factor MYC is often deregulated in cancer cells and is commonly associated with multidrug resistance and poor prognosis. Nonetheless, deregulated MYC activity also provides cancer cells with vulnerabilities that might be exploited for targeted pharmacological intervention. For example, high MYC expression is a negative prognostic factor in B‐cell lymphoma but also sensitizes tumor cells to drugs that suppress mitochondrial respiratory activity, such as the electron transport chain (ETC) complex I inhibitor IACS‐010759. While this selective pharmacogenetic interaction relies on the activation of specific signaling pathways (in particular, the integrated stress response and intrinsic apoptosis), the primary mechanisms through which MYC activation and ETC inhibition cooperate in triggering those processes remain unknown. Understanding those mechanisms might open new therapeutic perspectives against clinically problematic, multidrug‐resistant malignancies, as exemplified here by high‐grade MYC‐driven B‐cell lymphomas.

**Results**

Our data show that concurrent production of reactive oxygen species (ROS) upon MYC hyperactivation and IACS‐010759 treatment causes a lethal disruption of redox homeostasis. Most importantly, this phenomenon can be modulated by environmental and/or pharmacological cues. For example, high glucose concentrations boost NADPH production through the pentose phosphate pathway (PPP), favoring regeneration of reduced glutathione (GSH) and ROS scavenging, thus protecting from IACS‐010759‐induced cell death. Reciprocally, compounds that interfere with either GSH synthesis or regeneration increase the cytotoxic effects of IACS‐010759. A similar increase can be obtained by combining IACS‐010759 with high‐dose ascorbate (vitamin C), known to exert pro‐oxidant effects. In a preclinical setting, combining IACS‐010759 and high‐dose ascorbate provides synergistic antitumoral activity against xenografts of aggressive, MYC‐driven lymphomas, including Burkitt's and MYC/BCL2 “double‐hit lymphoma.”

**Impact**

Our results provide a therapeutic paradigm for MYC‐overexpressing B‐cell lymphomas, exploiting their vulnerability to oxidative stress. Given the synergistic action of IACS‐010759 and high‐dose ascorbate, we surmise that this association may allow to achieve effective therapeutic windows at lower doses of IACS‐010759, potentially circumventing its reported toxic effects. Most importantly, beyond IACS‐010759, we propose that other drugs interfering with mitochondrial activity—such as the antibiotic tigecycline—may show similar synergy with high‐dose ascorbate, pointing to the repurposing of these compounds for combinatorial treatment of high‐grade B‐cell lymphomas, and potentially other forms of MYC‐driven cancer.

## Introduction

The *MYC* proto‐oncogene and its product, the MYC transcription factor, have a central role in cellular growth control and are widely deregulated in many cancers, where they contribute to many facets of malignant transformation (Hsieh *et al*, 2015; Kress *et al*, 2015; Dhanasekaran *et al*, 2022) and are generally recognized as adverse prognostic factors (Kalkat *et al*, 2017; Donati & Amati, 2022), as best exemplified by diffuse large B‐cell lymphoma (DLBCL; Li *et al*, 2018; Bisso *et al*, 2019; Rosenwald *et al*, 2019). In particular, concurrent genomic alterations involving the *MYC* and *BCL2* proto‐oncogenes are present in a small subset of DLBCL (5–10%), traditionally named “double‐hit lymphomas” (DHL; now also categorized as “High‐Grade B‐cell Lymphoma”; Swerdlow *et al*, 2016), characterized by poor therapeutic responses and dismal prognosis (Bisso *et al*, 2019; Davies, 2019; Dunleavy, 2021; Zhuang *et al*, 2022). *MYC*‐driven tumors show oncogene addiction, indicating that MYC and a subset of its target genes are required for tumor maintenance, based on a diversity of cell‐intrinsic and ‐extrinsic mechanisms (Bisso *et al*, 2019; Dhanasekaran *et al*, 2022), and may thus represent potential therapeutic targets for effective pharmacological treatment of DHL and other MYC‐driven malignancies. Besides attempts to target MYC directly (Whitfield & Soucek, 2021; Llombart & Mansour, 2022), much effort in the field was aimed at the identification of synthetic lethal interactions as means to develop targeted therapies against MYC‐associated tumors (Bisso *et al*, 2019; Thng *et al*, 2021; Donati & Amati, 2022).

Multiple studies linked MYC to mitochondrial biogenesis and activity (Li *et al*, 2005; Morrish & Hockenbery, 2014; Wolpaw & Dang, 2018), in particular via activation of nuclear genes encoding the mitochondrial RNA polymerase POLRMT (Oran *et al*, 2016) or mitochondrial ribosomal proteins (D'Andrea *et al*, 2016), leading to enhanced respiratory activity (Donati *et al*, 2022). Along those lines, we and others identified inhibition of the mitochondrial ribosome with the antibiotic tigecycline as a therapeutically viable strategy against MYC‐driven lymphoma (D'Andrea *et al*, 2016; Oran *et al*, 2016). Inhibiting mitochondrial protein synthesis interferes with the assembly of Electron Transport Chain (ETC) complexes and ultimately with oxidative phosphorylation (OxPhos; Rudler *et al*, 2019), suggesting that the latter might also be a limiting activity—and hence a direct therapeutic target—downstream of MYC. In line with this concept, we reported that MYC‐ and OxPhos‐associated gene signatures were highly correlated in DLBCL and that a specific inhibitor of ETC complex I, IACS‐010759 (Molina *et al*, 2018), selectively killed MYC‐overexpressing cells by inducing intrinsic apoptosis (Donati *et al*, 2022). Accordingly, the antiapoptotic protein BCL2 counteracted killing by IACS‐010759 and, reciprocally, the BCL2 inhibitor venetoclax strongly synergized with IACS‐010759 against DHL (Donati *et al*, 2022), as also reported with tigecycline (Ravà *et al*, 2018). Hence, MYC sensitized cells to OxPhos inhibition, providing new opportunities for targeted drug combinations against aggressive DLBCL subtypes, and possibly other MYC‐associated tumors.

Here, we set out to characterize the mechanistic basis for the synthetic lethal interaction between oncogenic MYC and IACS‐010759. We report that MYC activation and pharmacological inhibition of the ETC coordinately disrupt redox homeostasis, underlying induction of the Integrated Stress Response (ISR) and apoptosis by IACS‐010759 (Bajpai *et al*, 2020; Donati *et al*, 2022). Most importantly, this mechanism does not strictly depend on the reliance of tumor cells upon OxPhos and can be exploited to further enhance killing of MYC‐overexpressing cells by combining IACS‐010759 with other pro‐oxidant drugs, improving the therapeutic efficacy against high‐grade lymphomas.

## Results

### Disruption of redox homeostasis sensitizes MYC‐overexpressing cells to ETC inhibition

**Figure 1:**
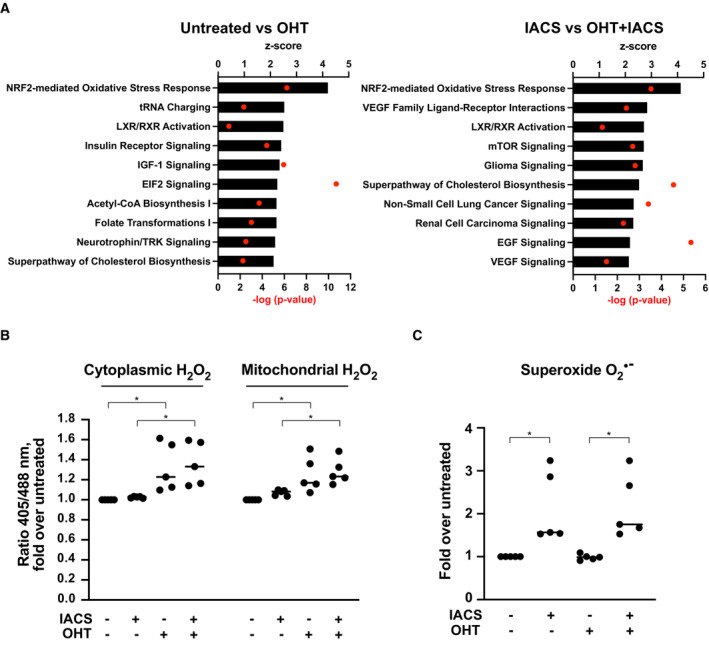
MycER activation causes ROS production and induces an oxidative stress response FL^MycER^ cells, primed or not with OHT (48 h), were treated with 135 nm IACS‐010759 (IACS) for 24 (A) or 48 h (B–D).
Top 10 OHT‐activated pathways (highest z‐score), as determined by IPA canonical pathway analysis on DEGs from cells treated or not with OHT (72 h), either alone (left) or in the presence of IACS‐010759 (for the last 24 h; right). Note that the RNA‐seq data and DEG lists used here are the same as in our previous study (Donati *et al*, 2022), while the IPA analysis presented here is new.H_2_O_2_ quantification, expressed as fold‐increase of the 405/488 nm fluorescence ratio in treated vs. untreated FL^MycER^ cells, expressing either the cytoplasmic (left) or mitochondrial (right) roGFP2‐ORP1 biosensor.Superoxide anion O_2_^·−^ production in treated vs. untreated FL^MycER^ cells, based on dihydroethidium staining. Data information: *P ≤ 0.05 (one‐way ANOVA). Each point in the graphs in (B and C) is from an independent biological replicate, each representing the average of thousands of events (single cells) in a distinct cell population, normalized to the untreated condition. Single‐cell measurement distributions from representative experiments are provided in Appendix Fig S1A and B, respectively. Source data are available online for this figure.

We previously showed that ectopic activation of the 4‐hydroxytamoxifen (OHT)‐responsive MycER™ chimaera (MycER) in the lymphoid precursor cell line FL5.12 (hereafter FL^MycER^ cells) augmented OxPhos‐associated gene expression and respiratory activity and sensitized cells to apoptotic killing by the ETC complex I inhibitor IACS‐010759 (Donati *et al*, 2022). To unravel additional MYC‐effector pathways that may mediate this synthetic lethal interaction, we further queried our previous transcriptomic data (Donati *et al*, 2022) with the Ingenuity Pathway Analysis package (IPA; see Materials and Methods). This analysis pointed to the Nrf2‐mediated oxidative stress response as the top OHT‐responsive pathway in FL^MycER^ cells, regardless of IACS‐010759 treatment (Fig 1A). Nrf2 is a transcription factor that under normal growth conditions is bound in the cytoplasm by the Keap1‐containing ubiquitin ligase complex and rapidly targeted to degradation. Oxidative stress triggers the release of Nrf2 from Keap1, resulting in its stabilization and migration to the nucleus, where it promotes an antioxidant transcription program (Baird & Yamamoto, 2020). MYC may promote Nrf2 activity at two levels: indirectly through the production of reactive oxygen species (ROS; Tanaka *et al*, 2002; Vafa *et al*, 2002; Cottini *et al*, 2015), or through direct transcriptional activation of the *Nrf2* locus (Liang *et al*, 2019). Through the latter, MYC activation may also lead to ROS reduction, as reported in some settings (DeNicola *et al*, 2011).

Based on the above observations, we sought to address whether—and how—modulation of oxidative stress may underlie the sensitization of MYC‐overexpressing cells to IACS‐010759. We first addressed the levels of hydrogen peroxide (H_2_O_2_), the most abundant cellular ROS, in FL^MycER^ cells expressing either the cytoplasmic or the mitochondrial variant of the H_2_O_2_ biosensor roGFP2‐ORP1 (Gutscher *et al*, 2009): as assessed by the 405/488 nm fluorescence ratio, MycER activation led to increased H_2_O_2_ levels in both compartments, while IACS‐010759 treatment showed negligible effects (Fig 1B and Appendix Fig S1A). Most relevant here, H_2_O_2_ is one of the species that can activate Nrf2 (Baird & Yamamoto, 2020), and may thus mediate this effect of MycER. Second, we monitored the superoxide anion O_2_^·−^, produced following impairment of the mitochondrial ETC, and in particular of complex I (Brand *et al*, 2004). Indeed, quantification with the fluorescent probe dihydroethidium (Rothe & Valet, 1990) revealed that, contrary to H_2_O_2_, O_2_^·−^ was induced upon IACS‐010759 treatment, with no significant contribution from MycER (Fig 1C). Notably, while O_2_^·−^ is less stable and abundant than H_2_O_2_, the reaction between those two species can produce the highly reactive ROS Hydroxyl radical (Collin, 2019). Thus, both MycER activation and IACS‐010759 treatment drive the production of oxidative species, which may underlie their cooperation toward cell killing.

**Figure 2:**
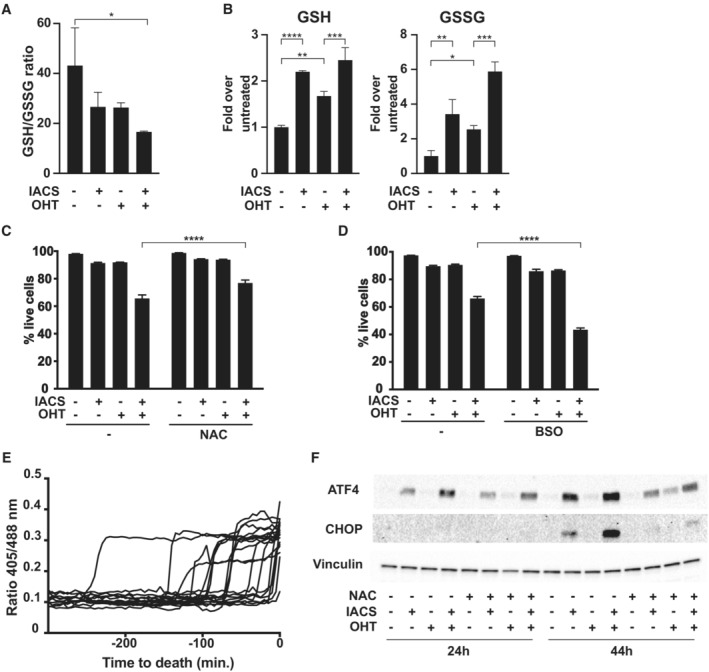
Disruption of redox homeostasis by IACS‐010759 induces cell death in MYC‐overexpressing cells FL^MycER^ cells were primed with 100 nM OHT (48 h) and/or treated with 135 nM IACS‐010759, as indicated.
A, BGlutathione redox state, given as (A) reduced to oxidized ratio and (B) glutathione quantification in FL^MycER^ cells, measured after 40 h of IACS‐010759 treatment.CCell viability, as determined by PI staining after 48 h of IACS‐010759 treatment, with or without the addition of 10 mM N‐acetyl‐cysteine (NAC).DSame as (C), with 50 μM buthionine sulfoximine (BSO).ETime‐lapse, single‐cell microscopic analysis of cytoplasmic glutathione redox potential before cell death, assessed as 405/488 nm fluorescence ratio from the Grx1‐roGFP2 biosensor in FL^MycER^ cells treated with OHT and IACS‐1010759 (36 h at the onset of filming). The time of death (*t* = 0) was scored based on the cellular incorporation of PI, present in the culture medium.FImmunoblot on lysates from FL^MycER^ cells treated as indicated, with the addition of 10 mM NAC concomitantly with IACS‐010759. Data information: In (A–D) *n* = 3 biological replicates; error bars: SD. *P ≤ 0.05; **P ≤ 0.01; ***P ≤ 0.001; ****P ≤ 0.0001 (one‐way ANOVA). Source data are available online for this figure.

To further monitor the oxidative stress induced by OHT and IACS‐010759 in FL^MycER^ cells, we quantified the ratio of reduced to oxidized glutathione (GSH and GSSG, respectively) after 40 h of IACS‐010759 treatment, a time preceding overt cell death (observed at ca. 48 h; Donati *et al*, 2022). Remarkably, each agent alone caused a moderate decrease in GSH/GSSG ratio, which became more pronounced in double‐treated cells (Fig 2A). Concomitant with these changes, the net levels of both GSH and GSSG were increased by both OHT and IACS‐010759 treatment (Fig 2B), most likely reflecting the activation of compensatory mechanisms to maintain redox homeostasis and favor survival.

We next sought to address whether the observed imbalances in redox homeostasis might be required for the death of OHT/IACS‐010759‐treated FL^MycER^ cells. Supplementation of the cultures with the ROS scavenger and glutathione precursor N‐acetylcysteine (NAC) reduced the killing of double‐treated cells (Fig 2C); of note, a similar effect of NAC was reported for tigecycline‐induced killing of MYC‐overexpressing U2OS cells (Oran *et al*, 2016). Reciprocally, the inhibitor of GSH synthesis buthionine sulfoximine (BSO) enhanced killing (Fig 2D). This effect of BSO in potentiating the cytotoxic action of IACS‐010759 was confirmed in two MYC‐rearranged human lymphoma cell lines, DoHH2 and Ramos, derived from a double‐hit and a Burkitt's lymphoma (BL), respectively (Appendix Fig S2A). Finally, time‐lapse microscopy on FL^MycER^ cells expressing Grx1‐roGFP2, a biosensor of cytoplasmic glutathione redox potential (Gutscher *et al*, 2008), revealed that an abrupt fall in GSH availability (as revealed by the increase in 405/488 nm fluorescence ratio) regularly preceded death in double‐treated cells (Fig 2E and Movie EV1). The observed time window between the drop in GSH and cell death was variable, ranging from few minutes to hours: this should be considered the product of a series of stochastic parameters that are variable from cell to cell (Skommer *et al*, 2011), including the expression of pro‐ and antiapoptotic genes, the time passed from previous mitotic division (Kirova *et al*, 2022) and others. Altogether, the above data strongly support a causative role of oxidative stress in the IACS‐010759‐induced killing of MYC‐overexpressing cells.

We and others previously showed that the integrated stress response (ISR) drives cell death in response to IACS‐010759 (Bajpai *et al*, 2020; Donati *et al*, 2022). Since ISR signaling can also be activated by oxidative stress (Costa‐Mattioli & Walter, 2020; Tian *et al*, 2021), we tested whether NAC could counteract the action of IACS‐010759 in triggering this response, as assayed by accumulation of the ISR‐associated transcription factors ATF4 and CHOP. Indeed, while both proteins readily accumulated in IACS‐010759 and—to a larger extent—in OHT/IACS‐010759‐treated cells (Donati *et al*, 2022), this effect was largely abrogated in the presence of NAC (Fig 2F). Finally, antibiotics that inhibit the mitochondrial ribosome and consequently suppress OxPhos activity (e.g., tigecycline and other tetracyclines) also activate the ISR (Bruning *et al*, 2014; Sasaki *et al*, 2020; Vendramin *et al*, 2021; Sanchez‐Burgos *et al*, 2022). As with IACS‐010759, tigecycline‐dependent induction of the ISR was quenched by NAC in FL^MycER^ cells (Appendix Fig S2B). These results point to a widespread role for ROS in ISR activation when interfering with OxPhos.

### Glucose and the pentose phosphate pathway maintain redox homeostasis in IACS‐010759‐treated cells

In all cell types examined so far, including OHT‐treated FL^MycER^ cells, the cytotoxic action of IACS‐010759 was suppressed by excess glucose in the culture medium (Molina *et al*, 2018; Naguib *et al*, 2018; Vangapandu *et al*, 2018; Donati *et al*, 2022), an effect that might be linked to the need to produce ATP through glycolysis upon blockade of the respiratory chain. Indeed, as assessed by glycolytic proton efflux rate (glycoPER) analysis, IACS‐010759 treatment increased the basal glycolytic rate and profoundly suppressed mitochondrial energy production in FL^MycER^ cells (Appendix Fig S3A and B); however, in contrast with cell killing, this effect of IACS‐010759 was independent from OHT priming. We previously reported that IACS‐010759‐induced cell death in OHT‐primed cells was not associated with ATP reduction and energy impairment in OHT‐primed cells (Donati *et al*, 2022). Altogether, these observations imply a distinct metabolic requirement for glucose—other than sustaining glycolysis for ATP production—in blocking the cytotoxic action of IACS‐010759.

**Figure 3:**
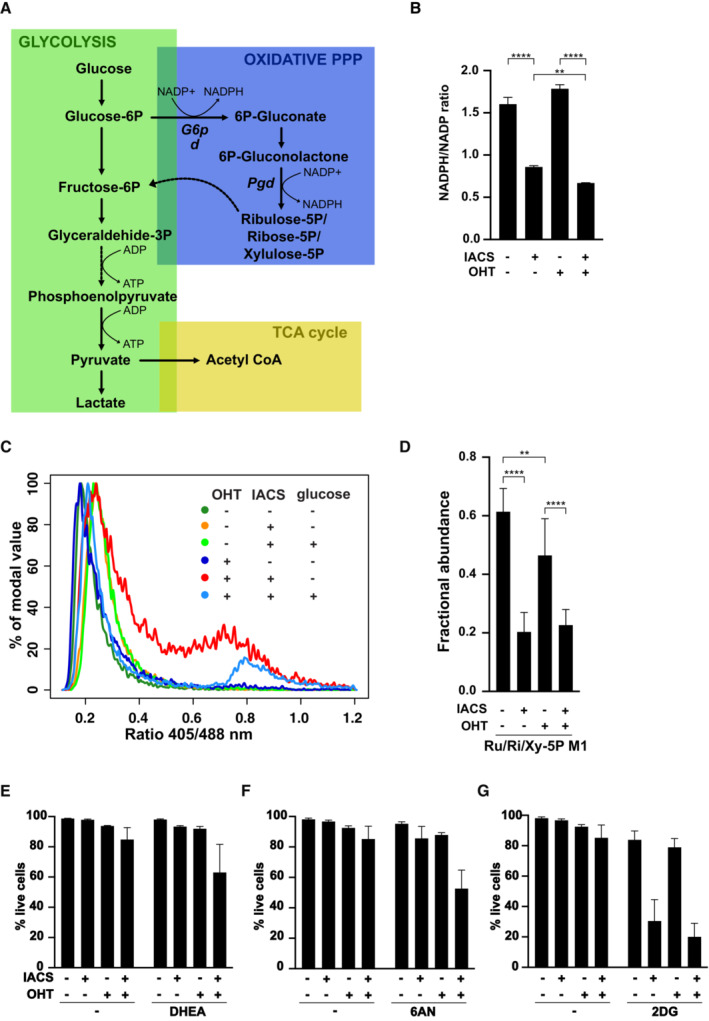
Pentose phosphate pathway protects MYC‐overexpressing cells from IACS‐010759‐induced toxicity ASchematic representation of glucose metabolism, highlighting NADPH‐ and ATP‐producing reactions.B–GFL^MycER^ cells were primed with 100 nM OHT and treated with 135 nM IACS‐010759, as indicated. (B) NADPH/NADP redox state in FL^MycER^ cells, assayed after 40 h of IACS‐010759 treatment. *n* = 3 biological replicates. **P ≤ 0.01; ****P ≤ 0.0001 (one‐way ANOVA). (C) cytoplasmic glutathione redox potential, assessed after 44 h of IACS‐010759 treatment in FL^MycER^ cells expressing the Grx1‐roGFP2 biosensor. Where indicated, 2.75 mM glucose was added to the medium and the measurement repeated after 3 h. (D) Fractional abundance of the 13C‐labeled glucose metabolites ribulose‐5P, ribose‐5P and xylulose‐5P (Ru/Ri/Xy‐5P), endpoints of the PPP oxidative phase, after 24 h of IACS‐010759 treatment. **P ≤ 0.01; ****P ≤ 0.0001 (two‐way ANOVA). (E–G) Cell viability after 40 h of IACS‐010759 treatment in the presence of (E) 50 μM dehydroepiandrosterone (DHEA), (F) 10 μM 6‐aminonicotinamide (6AN) or (G) 1 mM 2‐deoxyglucose (2DG). See Appendix Fig S3F for detailed statistical analysis. Data information: In (D, E, G) *n* = 6; in (F) *n* = 9; all biological replicates; error bars: SD. Source data are available online for this figure.

Among its multiple metabolic fates, glucose serves to regenerate NADPH from NADP through the action of G6pd and Pgd, two enzymes of the oxidative phase of the pentose phosphate pathway (PPP, Fig 3A). Most relevant in this context, the conversion of NADPH to NADP serves to recycle GSSG to GSH (Racker, 1955). Indeed, in parallel with the decline in GSH/GSSG ratio (Fig 2A), IACS‐010759 elicited significant drops in NADPH/NADP ratio (Fig 3B), an effect reinforced by co‐treatment with OHT. Thus, we hypothesized that glucose might prevent IACS‐010759‐induced killing through the maintenance of redox homeostasis. In line with this concept, monitoring of the Grx1‐roGFP2 biosensor revealed that the fall in GSH availability, which precedes cell death in OHT/IACS‐010759 double‐treated FL^MycER^ cells (Fig 2E), was reversed by adding glucose to the medium (Fig 3C).

To better understand the effects of Myc and IACS‐010759 on the oxidative PPP, we monitored the levels and activities of its two NADPH‐generating enzymes, G6pd and Pgd (Fig 3A). G6pd was induced upon OHT treatment, with no effect of IACS‐010759 (Appendix Fig S3C). Yet, while OHT had only a marginal effect on G6pd enzymatic activity, IACS‐010759 strongly suppressed it (Appendix Fig S3D). Pgd instead showed no significant variations with either OHT or IACS‐010759 (Appendix Fig S3C and D). Since G6pd catalyzes the rate‐limiting step of the PPP, we surmised that suppression of its activity by IACS‐010759 may reduce the overall glucose flux through this pathway: indeed, metabolic flux analysis with the isotopic tracer [1,2‐^13^C]glucose revealed that, regardless of OHT treatment, IACS‐010759 suppressed production of the final products on the oxidative PPP reactions—the phosphopentoses ribulose‐5‐P, ribose‐5‐P, and xylulose‐5‐P (Ru/Ri/Xy‐5P, Fig 3D).

Lactate is the end product of glycolysis; by using the [1,2‐^13^C]glucose tracer, lactate produced by glucose that passed directly through glycolysis can be distinguished from that produced by glucose processed through the PPP (Fig 3A): the former would be quantified as lactate M2 isotopomer, and the latter as lactate M1. Given the decreased PPP flux in IACS‐010759‐treated cells, we expected a reduced production of lactate M1: while apparent in our data, this effect remained below statistical significance (Appendix Fig S3E). On the contrary, IACS‐010759 treatment increased the relative abundance of lactate M2, produced by direct passage through glycolysis: this result is consistent with the increased basal glycolytic rate observed in IACS‐010759‐treated cells (Appendix Fig S3A and B). Regardless of MYC activation, rerouting glucose from the PPP to glycolysis would fulfill the need to maintain energetic homeostasis upon suppression of OxPhos.

To confirm the importance of the oxidative PPP for the selective killing of MYC‐overexpressing cells by IACS‐010759, we inhibited G6pd and Pgd (Fig 3A) with dehydroepiandrosterone (DHEA) and 6‐aminonicotinamide (6AN), respectively (Kohler *et al*, 1970; Raineri & Levy, 1970). Indeed, either of these compounds enhanced cell death selectively in OHT/IACS‐010759 double‐treated FL^MycER^ cells (Fig 3E and F; see Appendix Fig S3F for statistical analysis). In contrast, inhibiting the first step of glucose metabolism with 2‐deoxyglucose (2DG; Wick *et al*, 1957) sensitized FL^MycER^ cells to IACS‐010759 irrespective of OHT treatment (Fig 3G and Appendix Fig S3F), most likely as a consequence of energy impairment due to simultaneous inhibition of OxPhos and glycolysis (Vangapandu *et al*, 2018). Moreover, similar results were obtained in high glucose medium (Appendix Fig S3G), confirming that NADPH regeneration through the PPP is responsible for glucose‐mediated protection from IACS‐010759 cytotoxicity. Finally, either DHEA or 6AN potentiated killing by IACS‐010759 also in human MYC‐rearranged lymphoma cell lines (Appendix Fig S3H).

We then sought to confirm these results in a genetic model of PPP impairment obtained by ablation of *Pgd* through CRISPR‐Cas9 targeting. All of the Pgd KO FL^MycER^ clones obtained were heterozygous, with residual Pgd protein expression (Appendix Fig S3I), consistent with the essential nature of this gene, as defined in the Broad Institute Dependency Map (DepMap) portal (Ghandi *et al*, 2019). This notwithstanding, these *Pgd*‐targeted clones showed increased sensitivity to IACS‐010759 following OHT priming (Appendix Fig S3J).

Altogether, the above data show that glucose protects MYC‐overexpressing cells from IACS‐010759‐induced killing by sustaining NAPDH production through the oxidative phase of the PPP (Fig 3A), ensuring the regeneration of GSH required to maintain redox homeostasis.

### Ascorbate potentiates the pro‐oxidant and antitumoral effects of IACS‐010759

**Figure 4:**
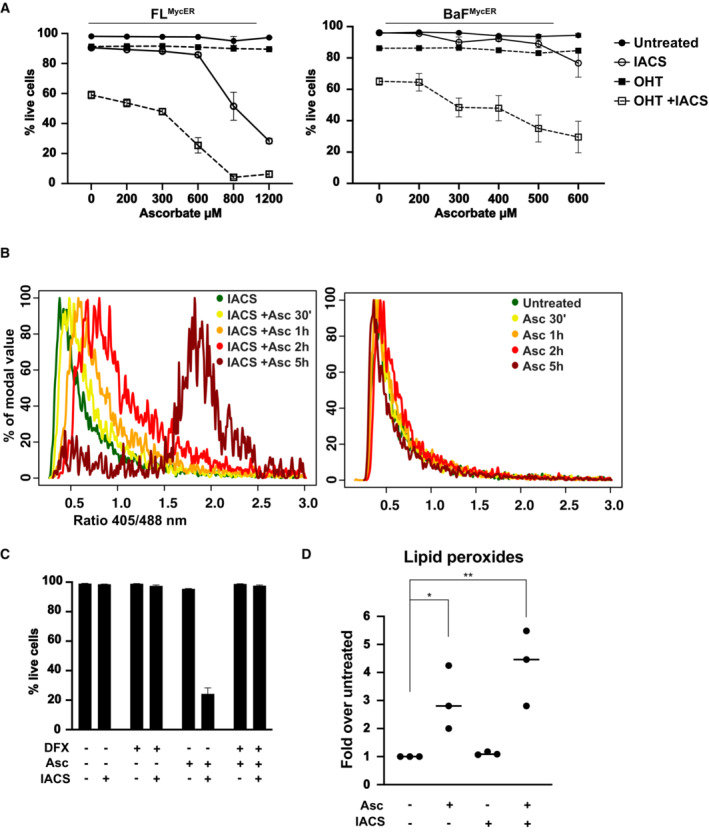
Ascorbate potentiates IACS‐010759‐induced cell death by increasing oxidative stress Viability of FL^MycER^ and BaF^MycER^ cells primed or not with 100 nM OHT and treated with 135 nM IACS‐010759 for 48 h and/or with ascorbate at the indicated concentration for 6 h. *n* = 3 biological replicates; error bars: SD.405/488 nm fluorescence ratio from the cytoplasmic Grx1‐roGFP2 reporter in FL^MycER^ cells treated with 400 μM ascorbate (Asc) for the indicated periods of time, either with IACS‐010759 (36 h, left) or without it (right). The same experiment with OHT‐primed cells is shown in Appendix Fig S4D.Cell viability of FL5.12 cells at the end of treatment (48 h IACS‐010759, 6 h Asc), in the presence or absence of 50 μM deferoxamine (DFX, added 1 h before Asc). *n* = 3 biological replicates; error bars: SD.Quantification of lipid peroxides in FL5.12 cells treated with 135 nM IACS‐010759 for 24 h and/or 400 μM Asc for 3 h. **P ≤ 0.01; *P ≤ 0.05 (one‐way ANOVA). Each point in the graph is from an independent biological replicate and represents the average of thousands of events (single cells) in a distinct cell population, normalized to the untreated condition. Single‐cell measurement distributions from a representative experiment are provided in Appendix Fig S4F. Source data are available online for this figure.

Given the protective role of antioxidant defenses, we hypothesized that pro‐oxidant agents might increase the cytotoxic action of IACS‐010759. Parenteral administration of a high dose of ascorbate (vitamin C) has been shown to have pro‐oxidant and anticancer activity in preclinical models (Chen *et al*, 2005, 2007, 2008; Di Tano *et al*, 2020). These results prompted several clinical trials using high‐dose ascorbate to treat advanced human cancer, in which ascorbate showed no activity if given as monotherapy (Hoffer *et al*, 2008; Stephenson *et al*, 2013), but had some efficacy when combined with chemotherapy (Monti *et al*, 2012; Welsh *et al*, 2013). Importantly, all of these studies concurred to show that ascorbate had minimal toxicity. We thus tested ascorbate in combination with IACS‐010759 in both FL^MycER^ and BaF^MycER^ cells (the latter derived from the Ba/F3 lymphoid cell line). As expected, OHT priming sensitized both MycER cell lines to killing by IACS‐010759 alone (Donati *et al*, 2022); most remarkably, ascorbate potentiated this effect without showing any cytotoxicity on its own (Fig 4A). In FL^MycER^ cells, in which a broader concentration range of ascorbate was tested, the highest concentrations of this vitamin allowed killing by IACS‐010759 in the absence of OHT priming.

Measurements with the roGFP2‐ORP1 biosensor showed that ascorbate treatment rapidly induced high levels of H_2_O_2_ in both the cytoplasm and mitochondria, with IACS‐010759 co‐treatment further enhancing this effect in the cytoplasm (Appendix Fig S4B). Consistent with H_2_O_2_ production, the Grx1‐roGFP2 reporter revealed that ascorbate caused a progressive and profound fall of glutathione redox potential in IACS‐010759‐treated cells (Fig 4B, left), while those treated with ascorbate alone (right) showed a moderate and transient reduction (2 h), followed by full recovery (5 h). Comparable results were obtained in the presence of OHT (Appendix Fig S4D).

Of note here, and somewhat counterintuitive, ascorbate blunted superoxide production in IACS‐010759‐treated cells (Appendix Fig S4C), which seem at odds with its pro‐oxidant effects. A possible explanation for this result could be that superoxide is being scavenged by the ascorbate radical (Nishikimi, 1975; Scarpa *et al*, 1983) at a rate similar to that achieved with dihydroethidium (Zhao *et al*, 2003), the fluorescent probe used for superoxide quantification.

Previous work linked the toxicity of ascorbate to ROS production through a Fenton reaction with the cell's labile iron pool (i.e., “free,” redox‐active intracellular iron; Schoenfeld *et al*, 2017). Since inhibition of mitochondrial complex I can increase the labile iron pool (Mena *et al*, 2011), the observed potentiation of ascorbate‐induced H_2_O_2_ production and pro‐oxidant activity by IACS‐010759 could be linked to this effect. In line with this scenario, the levels of two mRNAs known to be destabilized by free iron, *Tfrc* and *Slc11a2* (Anderson *et al*, 2012), were reduced upon IACS‐010759 treatment (Appendix Fig S4D). Furthermore, a short pretreatment with the ferric iron chelator deferoxamine (DFX) fully prevented cell death in IACS‐010759/ascorbate double‐treated cells (Fig 4C). Note that unlike DFX, excess glucose in the medium was unable to block the cytotoxic action of the IACS‐010759/ascorbate combination (Appendix Fig S4E), most likely due to insufficient ability of the PPP to compensate for the rapid GSH depletion seen in these experimental conditions (Fig 4B and Appendix Fig S4C). Finally, given the importance of iron in the combinatorial effects of IACS‐010759 and ascorbate, we investigated the involvement of ferroptosis, a form of regulated cell death initiated in response to lipid peroxidation by iron‐generated ROS (Jiang *et al*, 2021). Indeed, ascorbate treatment caused a marked increase in lipid peroxidation (Fig 4D); while IACS‐010759 had no effect alone, it showed a tendency (albeit below statistical significance) to reinforce the effect of ascorbate. Altogether, the above results suggest that ferroptosis contributes to the potentiation of IACS‐010759‐induced cell death by ascorbate.

**Figure 5:**
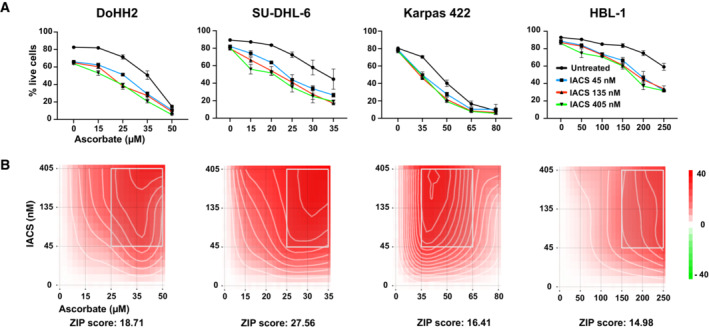
Ascorbate and IACS‐010759 synergistically induce cell death of Myc‐overexpressing DLBCL cells *in vitro* The DLBCL cell lines DoHH2, SU‐DHL‐6, Karpas 422 and HBL‐1 were treated with the indicated concentrations of IACS‐010759 and ascorbate, and cell viability determined after 24 h.
Live cell counts. *n* = 3 biological replicates; error bars: SD.Drug interaction landscapes and synergy scores, calculated according to the ZIP model: a positive ZIP score (> 10) signifies a synergistic interaction. The landscape identifies the doses at which the drugs either synergize (red) or antagonize each other (green)—the latter not observed here.
Source data are available online for this figure.

We then tested the combination of IACS‐010759 and ascorbate on DLBCL‐derived cell lines expressing high levels of MYC. In line with the data obtained in FL^MycER^ cells, ascorbate also increased IACS‐010759 mediated killing in these cells, with the two drugs displaying significant synergistic effects within defined concentration ranges (Fig 5A and B). The same was confirmed in *MYC*‐rearranged BL cells (Appendix Fig S5A). In summary, IACS‐010759 and ascorbate synergized *in vitro* to kill MYC‐overexpressing mature B‐cell neoplasms, regardless of their origin and molecular subtype (Appendix Fig S5B and C).

**Figure 6:**
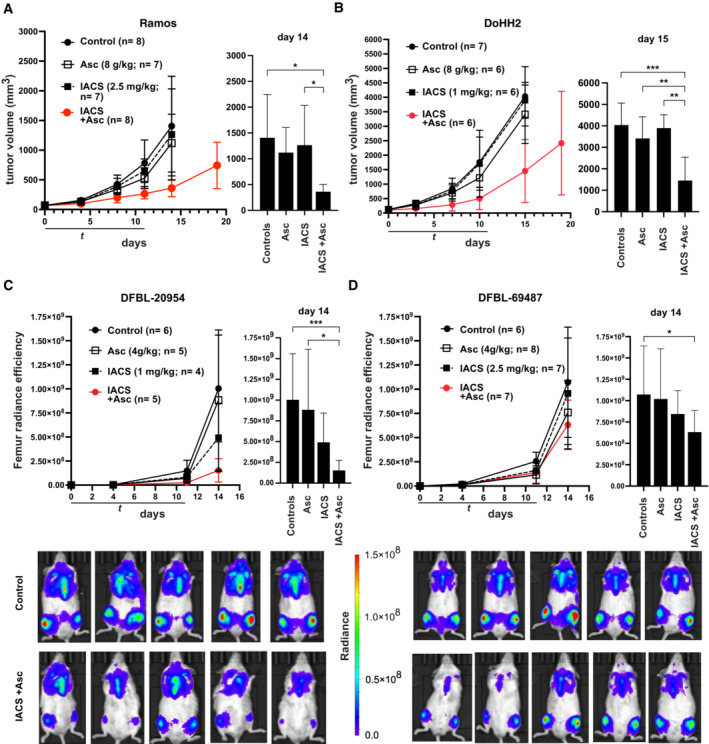
Combinatorial action of IACS‐010759 and ascorbate against Myc‐overexpressing lymphoma xenografts The indicated lymphoma cell lines and PDX samples were xenografted in recipient animals and tumors allowed to develop to detectable sizes prior to randomization and treatment with IACS‐010759 (IACS) by oral gavage and/or ascorbate (Asc) by intraperitoneal injection, as indicated (see Materials and Methods). Daily doses and the number of animals per group (*n*) are indicated in parenthesis.
A, BTumor progression (mm^3^) in CD1 nude mice bearing subcutaneous Ramos (A) or DoHH2 tumors (B).C, DTumor progression in NSG mice injected with the luciferase‐positive DHL‐derived PDX lines DFBL‐20954 (C) or DFBL‐69487 (D), as determined by *in vivo* imaging and quantification of bilateral femur radiance efficiency. Examples from the control and double‐treated groups are shown at the bottom. Data information: In each panel, the plot on the right shows the data for individual groups on the indicated day. Error bars: SD; *n* = animals per group. Right: one‐way between‐group ANOVA. *P ≤ 0.05; **P ≤ 0.01; ***P ≤ 0.001. Source data are available online for this figure.

To address whether this combinatorial activity could be exploited in a preclinical setting, CD1 nude mice were transplanted subcutaneously with either Ramos or DoHH2 cells: After tumor engraftment, the mice were treated with ascorbate and/or IACS‐010759 over 2 weeks. Remarkably, the growth of either Ramos or DoHH2 xenografts was significantly delayed by the combination, but not by each drug alone (Fig 6A and B). Similar effects were obtained with two DHL‐derived patient‐derived xenografts (PDX; Townsend *et al*, 2016), injected systemically in NSG mice, and monitored by whole‐body bioluminescence (Fig 6C and D). Note that one of the PDX tumors, PDX‐69487, showed a remarkable resistance to IACS‐010759 alone even if used at a higher dose; nonetheless, as with all other xenografts, the combination did cause a significant reduction in tumor growth relative to untreated controls. Altogether, these results demonstrate a potentiation of the antitumoral activity of IACS‐010759 by ascorbate on Myc‐overexpressing B‐cell lymphoma, identifying redox homeostasis as a valid therapeutic target to treat this type of cancer.

## Discussion

Current standard R‐CHOP immunochemotherapy achieves cure in 50–60% of patients suffering from Diffuse large B‐cell lymphoma (DLBCL), the most common form of B‐cell neoplasm. Refractory DLBCL is often progressive and fatal, with limited efficacy of tested rescue therapies (Crump *et al*, 2017). Indeed, substantial efforts are being invested toward the definition of the specific molecular features—such as oncogene translocations and mutational and transcriptional profiles—that may allow to identify those patients that will respond poorly to standard therapy and may instead benefit from alternative regimens (Reddy *et al*, 2017; Chapuy *et al*, 2018; Schmitz *et al*, 2018; Wright *et al*, 2020; Kotlov *et al*, 2021). Besides the translocation and/or overexpression of specific oncogenes such as *MYC* and *BCL2*, those features that are the most noteworthy here are transcriptome‐based classifiers, such as the so‐called *cell‐of‐origin* (COO; Alizadeh *et al*, 2000) and *comprehensive consensus clustering* (CCC; Monti *et al*, 2005). The latter in particular includes an “OxPhos” group, characterized by elevated expression of genes associated with respiratory activity: further work confirmed that this classification represents a bona fide metabolic signature, with “OxPhos” DLBCL cell lines being vulnerable to inhibition of mitochondrial fatty acid oxidation and glutathione synthesis (Caro *et al*, 2012). We recently reported that OxPhos‐ and *MYC*‐associated gene signatures are highly correlated in DLBCL (Donati *et al*, 2022) and that overexpressed MYC sensitizes cells to inhibition of OxPhos activity, either indirectly with tigecycline (D'Andrea *et al*, 2016) or directly with IACS‐010759 (Donati *et al*, 2022).

In the present work, we clarify that the MYC‐mediated sensitization to IACS‐010759 is brought about by a critical accumulation of oxidative stress, rather than increased reliance on OxPhos for energy metabolism. Ectopic MycER activation and IACS‐010759 treatment in the B‐lymphoid cell line FL5.12 drove the production of at least two distinct reactive oxygen species, namely H_2_O_2_ and O_2_^·−^, which together caused a disruption of redox homeostasis and participated in activating the integrated stress response, which ultimately instigates cell death under these conditions (Donati *et al*, 2022). These effects were consistently suppressed and reinforced, respectively, by the addition of compounds that up‐ and down‐modulated glutathione synthesis, pinpointing redox unbalance as the cause of selective killing by IACS‐010759.

The ability of cells to regenerate GSH from oxidized glutathione (GSSG) is dependent upon the availability of NADPH, which in turn is regenerated from NADP by utilizing different substrates: glucose in particular is the main source of NADPH in cancer cells when metabolized through the pentose phosphate pathway (PPP), under the control of G6pd and Pgd (Fig 3A; Patra & Hay, 2014). Most relevant here, the cytotoxic action of IACS‐010759 in different cell types was suppressed by excess glucose in the medium (Molina *et al*, 2018; Naguib *et al*, 2018; Vangapandu *et al*, 2018; Donati *et al*, 2022) and, as shown in our FL^MycER^ cells, the sensitizing effect of glucose deprivation was not necessarily linked to an impairment in energy homeostasis, pointing to additional protective effects of this sugar (Donati *et al*, 2022).

The data reported here reveal that the protective effect of glucose in MYC‐overexpressing cells is mediated by its antioxidant action through the PPP pathway. First, addition of glucose reversed the loss of reduced glutathione elicited by MycER activation and IACS‐010759 treatment in FL^MycER^ cells. Second, IACS‐010759‐treated cells showed a significant suppression of G6pd activity, as well as decreased production of phosphopentoses, the final products of the PPP oxidative phase (Ru/Ri/Xy‐5P, Fig 3A). Third, pharmacological inhibition of either G6pd or Pgd further enhanced the selective killing of MYC‐overexpressing cells by IACS‐010759. This result is in line with the previous finding that depletion of Pgd sensitized H1975 lung cancer cells to IACS‐010759 (Sun *et al*, 2019): in this regard, it is noteworthy that H1975 cells bear an amplified *MYC* locus and significantly overexpress the protein (Tateishi *et al*, 2016; Beaulieu *et al*, 2019). Finally, independently from OHT treatment, energy production in FL^MycER^ cells was mainly glycolytic (Appendix Fig S3B): thus, MYC‐induced sensitization to IACS‐010759 did not depend upon OxPhos‐driven ATP production, as was instead the case for IACS‐010759 mediated killing of glycolysis‐deficient cells (Molina *et al*, 2018). We conclude that the combined effects of MYC and IACS‐010759 render the cells dependent upon glucose and the PPP pathway to prevent the accumulation of lethal oxidative damage.

Altogether, the aforementioned findings are consistent with the long‐known pro‐oxidant effects of MYC (Tanaka *et al*, 2002; Vafa *et al*, 2002; Cottini *et al*, 2015) and reveal that these can sensitize cells to complementary pro‐oxidative cues—as achieved here by OxPhos inhibition—leading to critical disruptions of redox balance. This concept led us to test whether exacerbating the disruption of redox homeostasis caused by IACS‐010759 could reinforce its anticancer activity against MYC‐driven B‐cell lymphomas. Indeed, suppressing glutathione synthesis with BSO increased IACS‐010759 activity on MYC‐overexpressing B‐cells and lymphoma cell lines. We further developed this concept by combining IACS‐010759 with ascorbate, which has a pro‐oxidant activity when injected at pharmacological doses and has shown toxicity toward diverse tumor cells (Gonzalez‐Montero *et al*, 2022), including lymphoma (Chen *et al*, 2005, 2008; Di Tano *et al*, 2020). Remarkably, IACS‐010759 and ascorbate synergized *in vitro* to kill MYC‐overexpressing B‐cells, owing most likely to the cooperative induction of oxidative damage, including lipid peroxidation and ferroptosis. This combination also showed synergy in BL and DLBCL lymphoma cell lines of multiple molecular subtypes, not restricted to the “OxPhos” category (Appendix Fig S5B). Finally, IACS‐010759 and ascorbate also suppressed the growth of lymphoma xenografts *in vivo*.

Similar to what observed after ectopic MycER activation (Donati *et al*, 2022; Appendix Fig S3A), mitogenic stimulation of B‐cells coordinately potentiates glycolysis and mitochondrial respiration (e.g., Caro‐Maldonado *et al*, 2014) as well as ROS production (Wheeler & Defranco, 2012). Thus, we cannot *a priori* exclude that a pro‐oxidant therapeutic regimen such as IACS‐010759 and ascorbate may be toxic for activated B‐cells. However, we note that high‐dose ascorbate has already proven safe and tolerable in a clinical setting, either alone or in association with platinum‐based and other ROS‐producing chemotherapeutic agents (Bottger *et al*, 2021). Moreover, high‐dose ascorbate reinforced anticancer immunotherapy in multiple solid tumor models (Magri *et al*, 2020), implying that it does not impair—or rather may favor—anticancer immunity: It will be of high interest to address whether the same may be true in combination with IACS‐010759 or other mitochondrial inhibitors.

The combinatorial action of IACS‐010759 and ascorbate unraveled here might prove to be relevant in diverse clinical settings. First, high‐dose ascorbate might increase the therapeutic window of OxPhos inhibitors, allowing their administration at clinically safe doses. This is a critical priority indeed, since a recent phase I trial revealed mechanism‐related toxicity of IACS‐010759, limiting its usage at effective antitumoral doses (Yap *et al*, 2023). Similar problems were encountered with the complex I inhibitors BAY‐87‐2243 and ASP4132 (Xu *et al*, 2020; Janku *et al*, 2021). Second, our findings might provide a much‐needed alternative treatment for some of the most aggressive forms of DLBCL that are refractory to front‐line R‐CHOP immunochemotherapy. MYC translocation and/or overexpression have been associated with reduced survival in these patients, especially if associated with alterations of the BCL2 oncogene (Li *et al*, 2018; Rosenwald *et al*, 2019): our present results point to MYC as a biomarker for a therapeutic approach based on IACS‐010759 and ascorbate.

Of note here, our previous studies highlighted therapeutic synergies between either IACS‐010759 (Donati *et al*, 2022) or tigecycline (Ravà *et al*, 2018) and the BCL2 inhibitor venetoclax against the aggressive DHL subtype, defined by joint translocation of *MYC* and *BCL2*. Having now shown that ascorbate reinforces the effect of IACS‐010759 on the same DHL lines, it can be hypothesized that a triple‐combination of IACS‐010759, ascorbate, and venetoclax may further improve the therapeutic window and treatment efficacy on these highly aggressive lymphomas.

Besides DLBCL, other tumor types showed an association of MYC and/or OxPhos gene signatures with increased resistance to various therapies, including ibrutinib in mantle cell lymphoma, neoadjuvant therapy in triple‐negative breast cancer, and venetoclax in acute myeloid leukemia (Lee *et al*, 2017; Sharon *et al*, 2019; Zhang *et al*, 2019). In all of these cases, the treatment‐refractory cancer cells were shown to be sensitive to pharmacological inhibition of OxPhos: our results suggest that additional combination with a pro‐oxidant drug like ascorbate may further improve the effectiveness of this therapeutic strategy. Finally, a recent report linked activation of the MYC paralogue MYCN in neuroblastoma to dependence on cysteine import and synthesis, needed to produce glutathione and avoid ferroptotic cell death (Alborzinia *et al*, 2022). Altogether, it is reasonable to expect that the combination of IACS‐010759 with ascorbate or other ferroptotic inducers might be effective against multiple classes of MYC‐associated tumors.

## Materials and Methods

### Chemicals and biochemical assays

IACS‐010759 (from the Institute for Applied Cancer Science at MD Anderson; Molina *et al*, 2018) and 4‐hydroxytamoxifen (Merck Life Science, Darmstadt, Germany) were dissolved in DMSO and ethanol, respectively; sodium L‐ascorbate, N‐acetyl‐L‐cysteine (NAC), L‐buthionine sulfoximine, 6‐aminonicotinamide, deferoxamine mesylate (Merck Life Science), dehydroepiandrosterone (Cayman Chemical Co., Ann Arbor, MI, USA), and tigecycline (Carbosynth, Newbury, UK) were dissolved in water.

GSH/GSSG and NADPH/NADP were quantified with luminescence‐based assay kits (Promega, Madison, WI, USA) and the enzymatic activities of G6pd and Pgd with colorimetric assay kits (BioVision/Abcam, Waltham, MA, USA), according to manufacturer's specifications.

### Cell lines

The human lymphoma cell lines were DoHH‐2 (CVCL_1179), SU‐DHL‐6 (CVCL_2206), Karpas 422 (CVCL_1325), HBL‐1 (CVCL_4213), Ramos (CVCL_0597) and Raji (CVCL_0511), and the murine B‐lymphoid cell lines FL5.12 (CVCL_0262) and Ba/F3 (CVCL_0161) expressing MycER™ (FL^MycER^, BaF^MycER^; Donati *et al*, 2022) were grown in regular RPMI medium (Euroclone, Pero, Italy), which includes 2 mM glutamine and 11 mM glucose, supplemented with 10% fetal bovine serum (FBS) and, for FL5.12 and Ba/F3 cells, 2 and 1 ng/ml murine interleukin 3 (PeproTech, Rocky Hill, NJ, USA), respectively. Prior to experiments involving IACS‐010759, all cells were passaged in glucose‐free RPMI‐1640 medium (Thermo Fisher Scientific, Waltham, MA, USA), which includes 2 mM glutamine, supplemented with 10% FBS and 2.75 mM glucose. To induce MycER activity, 100 nM 4‐hydroxytamoxifen (OHT; Merck) was added to the medium 48 h before treatment with IACS‐010759 or other drugs, as indicated. All cells were incubated at 37°C in a humidified air atmosphere supplemented with 5% CO_2_. SU‐DHL‐6, Raji and Ramos cell lines were imported from the ATCC repository (https://www.lgcstandards‐atcc.org); Ba/F3, DoHH‐2 and Karpas 422 cells were imported from the DSMZ repository (https://www.dsmz.de); FL5.12 and HBL‐1 cells were a gift from Pier Giuseppe Pelicci and Enrico Derenzini, respectively. All lines were stocked and made available by IEO's core Tissue Culture facility, where they were also validated and tested for mycoplasma infection.

### Xenograft models and treatment

5 × 10^6^ DoHH‐2 or Ramos cells were xenografted subcutaneously in sublethally irradiated (3 Gray), 8 weeks old, female CD1‐nude nu/nu mice (IMSR_ENV:HSD‐069, Envigo Indianapolis, IN, USA) and expanded by serial subcutaneous transplantation of tumor fragments. Tumors were allowed to grow for about 10–14 days, followed by assessment with a digital caliper to exclude outliers and form experimental groups with comparable average and variance of tumor size at the start of treatments (days 0). Tumor volumes were then measured twice a week with a digital caliper and calculated as 1/2 length × width^2^ (mm^3^). The following treatment schemes were used: daily oral gavage with IACS‐010759 (1 or 2.5 mg/kg, as indicated in the figures) for 5 days, followed by 2 days off and a repeat of the same scheme, for a total of 12 days; intraperitoneal injection of ascorbate twice a day (separated by 8 h) to reach a daily dose of 4 or 8 g/kg for 5 days, followed by 2 days off and a repeat of the same scheme. IACS‐010759 was suspended in 0.5% methylcellulose (Merck Life Science), while ascorbate was dissolved in physiological saline.

The DHL patient‐derived xenografts (PDX) DFBL‐20954‐V3‐mCLP and DFBL‐69487‐V3‐mCLP (Townsend *et al*, 2016) were obtained from the Dana Farber Cancer Institute Center for Patient Derived Models (CPDM). 1 × 106 cells were xenografted via tail vein injection into 8‐week‐old male NSG mice (IMSR_JAX:005557, Charles River, Calco, Italy). Tumor engraftment was confirmed 7 days after transplant by whole‐body imaging on an IVIS Lumina III platform following intraperitoneal injection of 150 mg/kg XenoLight D‐Luciferin (PerkinElmer, Waltham, MA, USA) and anesthesia with isoflurane. The animals were subsequently randomly distributed in the different experimental groups to start the treatment protocol with IACS‐010759 and/or ascorbate, as described previously. The response to treatment was assessed by whole‐body imaging at Days 4, 11, and 14. The data were analyzed with the Living Image Software, version 4.2 (Caliper Life Sciences, Hopkinton, MA, USA). Radiant efficiency was quantified bilaterally on femurs based on the epifluorescence signal as indicated in the user manual.

The size of experimental groups with animals carrying either cell line‐ or PDX‐derived tumors was chosen based on previous results (Donati *et al*, 2022) in order to reveal biologically relevant effects with sufficient statistical power.

Experiments involving animals were done in accordance with the Italian Laws (D.lgs. 26/2014), which enforces Dir. 2010/63/EU (Directive 2010/63/EU of the European Parliament and of the Council of 22 September 2010 on the protection of animals used for scientific purposes), and authorized by the Italian Health Ministry with project nr. 173/2021‐PR. Mice were housed in individually ventilated caging (IVC) systems (Sealsafe Plus, Tecniplast, Buguggiate, Italy), on autoclaved sawdust bedding (Lignocel® 3/4; Rettenmaier & Sohne, Ellwangen‐Holzmühle, Germany), provided with autoclaved diet (VRF1 (P), SDS, Witham, UK) and autoclaved water *ad libitum*. Animals were handled and treated in laminar flow hoods (CS5 Evo and BS48, Tecniplast, Buguggiate, Italy).

### Time‐lapse analysis of the Grx1‐roGFP2 biosensor

Retroviral pLPCX vectors expressing the mitochondrial or cytoplasmic form of Grx1‐roGFP2 (Gutscher *et al*, 2008) and roGFP2‐ORP1 (Gutscher *et al*, 2009) were obtained from Addgene (Addgene, Watertwon, CA, USA) and used to transduce FL5.12^MycER^ cells, followed by a one‐week selection with puromycin (AdipoGen AG, Liestal, CH) 1.5 μg/ml. Cells expressing either form of Grx1‐roGFP2 were primed with OHT and treated with IACS‐010759 for approximately 36 h and then transferred to glass bottom petri dishes (MatTek Corporation, Ashland, MA, USA) in the continuous presence of the drugs, with the addition of 1 μg/ml PI to mark dead cells. The cultures, kept at 37°C and 5% CO_2_, were imaged with a Leica SP8 FSU confocal microscope (Leica Microsystems, Wetzlar, Germany) through a 63×/1.4NA oil immersion objective lens. The oxidized and reduced forms of the Grx1‐roGFP2 biosensor were excited by the 405 and 488 nm laser lines, respectively. The emitted fluorescence for both forms was collected with an opened pinhole (2.7 AU) in the 500–540 nm acquisition window and sequentially acquired with the same HyD detector set in counting mode with a pixel size of 180 nm. The PI molecule was excited with the 561 nm laser line and the emitted signal collected between 590 and 700 nm. Images were acquired every 5 min for 12 h.

The ratio between the emission intensities of oxidized and reduced Grx1‐roGFP2 was calculated thanks to a custom‐made ImageJ/Fiji macro. Briefly, after background subtraction, the combination of the two signals was obtained and the resulting image (combined image) was filtered with a Gaussian filter and segmented with the Otsu algorithm. The resulting mask was applied to the original images of oxidized and reduced Grx1‐roGFP2 to obtain the ratio image. To segment the single cells in the images at fixed time‐points, the combined image was segmented using a more permissive algorithm (Li) and the resulting binary image subjected to the “analyze particle” function of ImageJ. The region‐of‐interest (ROI) values of single cells were then used to calculate the oxidized/reduced Grx1‐roGFP2 fluorescence ratio in every segmented cell. Single cells were manually tracked to record the Grx1‐roGFP2 ratio and PI signal intensity over time.

### Flow cytometry

Flow cytometry was conducted on a MACSQuant Analyzer (Miltenyi Biotec, Bergisch Gladbach, Germany) and data analyzed with FlowJo software (version 10.6.1; BD Biosciences, Franklin Lakes, NK, USA). For cell viability counts, cells were resuspended in ice‐cold PBS in the presence of propidium iodide (PI) 1 μg/ml. Viability was expressed as percentage of live (PI negative) cells on total cells counted.

To quantify the fluorescence from Grx1‐roGFP2 and roGFP2‐ORP1 biosensors, the oxidized and reduced forms were analyzed with the V2 (405/585–40 nm) and B1 (488/525–50 nm) channels, respectively. Immediately before counting, PI 1 μg/ml was added to the medium to exclude dead cells.

Quantification of superoxide anion was performed by staining live cells with 10 μM dihydroethidium (Thermo Fisher Scientific): Following 30 min of incubation with the reagent, cells were washed and resuspended in phosphate‐buffered saline for flow cytometric analysis. Immediately before counting, 4′,6‐diamidino‐2‐phenylindole (DAPI; Merck Life Science) 0.1 μg/ml was added to the medium to exclude dead cells.

Quantification of lipid peroxidation was performed by staining cells with 10 μM BODIPY 581/591 C11 (Thermo Fisher Scientific): Following 10 min of incubation, ascorbate was added where appropriate, and then, the cells were incubated for 3 h before being washed and resuspended in phosphate‐buffered saline for flow cytometric analysis.

### Immunoblot analysis and cell fractionation

Immunoblot analysis was performed as described previously (Donati *et al*, 2022). For the analysis of subcellular fractions, the same numbers of cells from each sample were lysed 5′ on ice with NP‐40 fractionation buffer (10 mM Hepes pH 7.4 1, 250 mM Sucrose, 25 mM KCl, 2 mM MgCl_2_, 1 mM EGTA, 0.1% NP‐40, 1 mM PMSF). This total lysate was then centrifuged at 1,000 × *g* for 5 min, and the supernatant collected as cytoplasmic fraction. The nuclear fraction was obtained by washing the pellet twice with fractionation buffer without NP‐40 and finally resuspending it in Laemmli buffer. The following antibodies were used: mouse monoclonals against vinculin (hVIN‐1, Merck Life Science; 1:20,000) and CHOP (L63F7, Cell Signaling Technology, Danvers, MA, USA; 1:1,000); rabbit monoclonals against ATF4 (D4B8, Cell Signaling Technology; 1:1,000), G6pd (D5D2, Cell Signaling Technology; 1:1,000), Pgd (EPR6565, Abcam, Cambridge, UK; 1:1,000), and MYC (Y69, Abcam; 1:1,000).

### Glycolytic proton efflux rate analysis

Proton efflux rate (PER) analysis was performed on a Seahorse XFe96 Analyzer (Agilent Technologies, Santa Clara, CA, USA) using Agilent Seahorse XF Glycolytic Rate Assay kit, following the manufacturer's instructions. Before the assay, FL^MycER^ cells were treated with OHT and IACS‐010759 for 72 and 24 h as indicated. The cells were then counted and attached to 96‐well Seahorse cell culture microplates, precoated with Corning™ Cell‐Tak (Life Sciences) according to the manufacturer's instructions, at a density of 80,000 cells per well, in XF RPMI Medium pH 7.4 with 1 mM HEPES (Agilent Technologies) supplemented with 2.75 mM glucose, 1 mM sodium pyruvate, and 2 mM L‐glutamine. The plate was incubated at 37°C for 1 h in a non‐CO_2_ incubator. After ECAR baseline measurements, 0.5 μM rotenone plus 0.5 μM antimycin A and 50 mM 2‐deoxyglucose (2‐DG) were added sequentially to each well. The results were analyzed using the Seahorse Wave Desktop Software Version 2.6 (Agilent Technologies) and normalized by cell number using CyQUANT Cell Proliferation Assay (Thermo Fisher Scientific). Data were exported into the XF Report Generator for calculation of the parameters from the Glycolytic Rate Assay. Results are mean ± SD of minimum eight technical replicates and representative of two independent experiments.

### Metabolic flux analysis

For metabolic flux analysis, cells were exposed for 4 h to 1.5 mM D‐[1,2‐^13^C_2_]glucose in complete medium containing 1.5 mM glucose. Cells were then harvested and washed twice in ice‐cold PBS, and the pellets were snap‐frozen in liquid nitrogen. Pellets were then resuspended in 250 μl methanol/acetonitrile 1:1 and spun at 20,000 *g* for 5 min at 4°C. Supernatant was then passed through a regenerated cellulose syringe filter, dried, and resuspended in 100 μl of MeOH for subsequent analysis.

We used an ExionLC™ AC System (AB Sciex, Framingham, MA, USA) coupled with an API‐3500 triple quadrupole mass spectrometer (AB Sciex). Quantification of different metabolites was performed using a cyano‐phase LUNA column (50 × 4.6 mm, 5 μm; Phenomenex, Torrance, CA, USA) by a 5 min run in negative ion mode. Mobile phases were (A) water and (B) 2 mM ammonium acetate in MeOH; the gradient was isocratic 90% B with a flow rate of 500 μl/min. MultiQuant™ software (version 3.0.2, AB Sciex) was used for data analysis and peak review of chromatograms. The identity of all metabolites was confirmed using pure standards.

### CRISPR‐Cas9 engineering of knockout cell lines

The *Pgd* gene knockout clones were obtained as previously described for other genes (Donati *et al*, 2022). Briefly, two sites on the gene were targeted: one close to, and the other ca. 100 bases downstream of the start codon. The genomic sequences targeted by the corresponding sgRNAs were the following: GCGAAGGACCGAGCGCTCCG and GGAGACCCAGGCGACCACCG. Each sgRNA was ligated into a PX458 plasmid (Addgene; plasmid # 48138, a gift from Feng Zhang): 1 μg of plasmid was used to electroporate in 4 × 10^5^ FL5.12 cells using the Neon Transfection System (Thermo Fisher Scientific). After 2 days, GFP‐positive cells were sorted on a FACSMelody (BD Biosciences) and single clones isolated by limiting dilution followed by expansion *in vitro*.

### Quantification and statistical analysis

Drug interaction landscapes and delta scores for synergy were based on the ZIP model in SynergyFinder (Ianevski *et al*, 2017). Comparisons between treatments for *in vitro* cell culture and biochemical experiments were carried out by one‐way or two‐way ANOVA. Partitioning the interaction effects in two‐way ANOVA allowed us to test for particular differences among treatments. Between treatment tests for *in vivo* tumor growth were performed by one‐way ANOVA. Comparisons between treatment tests for *in vivo* tumor growth were performed by one‐way ANOVA. Normal distribution and homogeneity of variances of data analyzed by ANOVA were confirmed by Shapiro–Wilk *W* and Levene tests, respectively. When pairwise comparisons of means were needed, *post hoc* tests were made according to Tukey's procedure. GraphPad PRISM 8 (RRID: SCR_002798) or R (RRID:SCR_001905) software was used for all analyses. The numbers of independent biological replicates are indicated in the figures. No blinding was done.

### RNA‐seq analysis

The RNA‐seq data and differentially expressed genes (DEGs) called in FL^MycER^ cells treated with OHT and/or IACS‐010759 were described in our previous work (Donati *et al*, 2022) and are accessible through NCBI's Gene Expression Omnibus (GEO; RRID: SCR_005012; GSE149073). Canonical pathway analysis to identify the most significantly activated pathways upon MycER activation with OHT (based on z‐score) was performed with the Ingenuity Pathway Analysis (IPA) software package (QIAGEN, Venlo, The Netherlands; RRID: SCR_008653).

## Author contributions

**Bruno Amati:** Conceptualization; formal analysis; supervision; funding acquisition; writing – original draft; project administration; writing – review and editing. **Giulio Donati:** Conceptualization; formal analysis; supervision; investigation; visualization; methodology; writing – original draft; project administration; writing – review and editing. **Paola Nicoli:** Investigation. **Alessandro Verrecchia:** Investigation. **Veronica Vallelonga:** Investigation. **Ottavio Croci:** Software; formal analysis. **Simona Rodighiero:** Formal analysis; investigation; visualization. **Matteo Audano:** Formal analysis; investigation. **Laura Cassina:** Formal analysis; investigation. **Aya Ghsein:** Investigation. **Giorgio Binelli:** Formal analysis. **Alessandra Boletta:** Formal analysis; supervision. **Nico Mitro:** Formal analysis; supervision.

## Disclosure and competing interests statement

The authors declare that they have no conflict of interest.

## Supporting information



Appendix

Movie EV1

Source Data for Figure 1

Source Data for Figure 2

Source Data for Figure 3

Source Data for Figure 4

Source Data for Figure 5

Source Data for Figure 6

## Data Availability

This study includes no data deposited in external repositories.
