# Supplementary material for: Oxidative stress enhances the therapeutic action of a respiratory inhibitor in MYC‐driven lymphoma
Source: EMBO Mol Med. 2023 May 9;15(6):e16910. doi: 10.15252/emmm.202216910 (PMC10245039; doi:10.15252/emmm.202216910)
Supplement: Supplementary file 1 — Appendix [file EMMM-15-e16910-s009.pdf]

Oxidative stress enhances the therapeutic action of a respiratory inhibitor in MYC-driven lymphoma

Giulio Donati *et al*. 2023

APPENDIX

Table of contents:

Appendix Figure S1	2
Appendix Figure S2	3
Appendix Figure S3	4
Appendix Figure S4	6
Appendix Figure S5	7

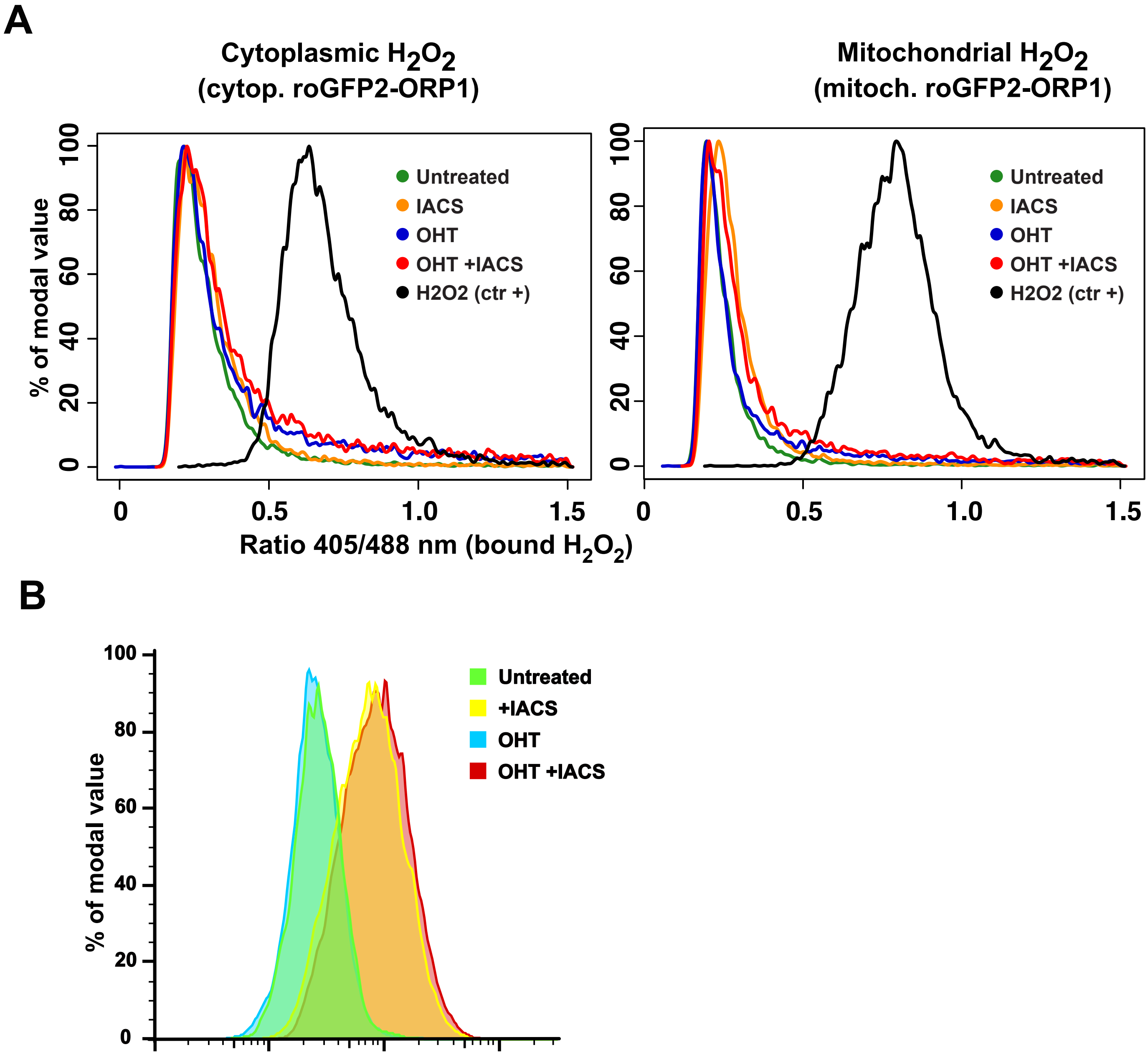

Appendix Figure S1. MycER activation causes ROS production and induces an oxidative stress response. (**A**) Representative distribution plots for the 405/488 nm fluorescence ratio with the cytoplasmic (left) and mitochondrial (right) roGFP2-ORP1 biosensor in OHT- and/or IACS-010759-treated FL^{MycER} cells (as in Figure 1B). As a positive control (ctr +), cells were treated with 200 µM H_2O_2 immediately before cytometric analysis. (**B**) Representative flow cytometry of superoxide quantification by dihydroethidium staining of FL^{MycER} cells, primed with 100 nM OHT and treated with 135 nM IACS-010759 for 24h where indicated (as in Figure 1C).

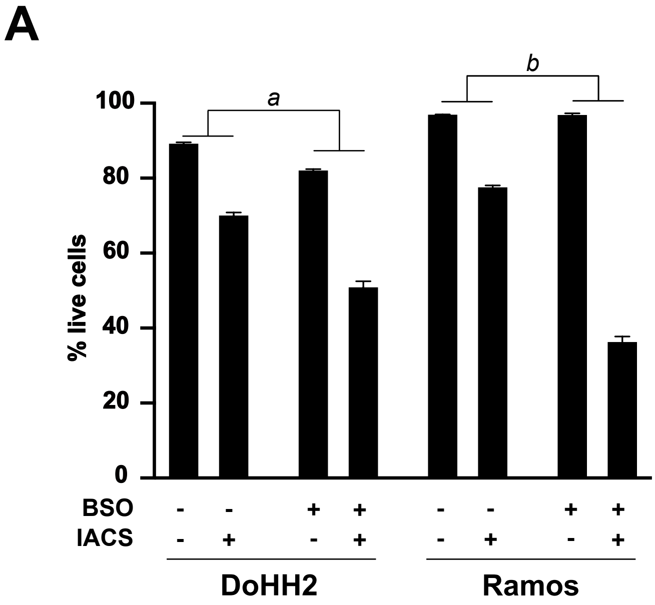

	$F_{1,8}$	p-value
a	125	3.65E-06
b	1799	1.05E-10

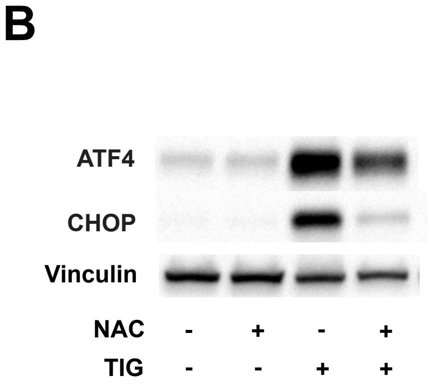

Appendix Figure S2. Disruption of redox homeostasis by IACS-010759 induces cell death in Myc-overexpressing cells. (**A**) Representative Cell viability of DoHH2 and Ramos lymphoma cells treated where indicated with 135 nM IACS-010759 for 40 hours, either alone or in the presence of 50 μM BSO. Error bars: SD (n=3). The table shows the two-way ANOVA analysis for IACS-010759-induced cell death in the presence and absence of BSO. (**B**) Immunoblot on lysates from FL^{MycER} cells treated with 5 μM tigecycline (TIG) for 48 hours, in the presence or absence of 10 mM NAC, as indicated.

A B C D

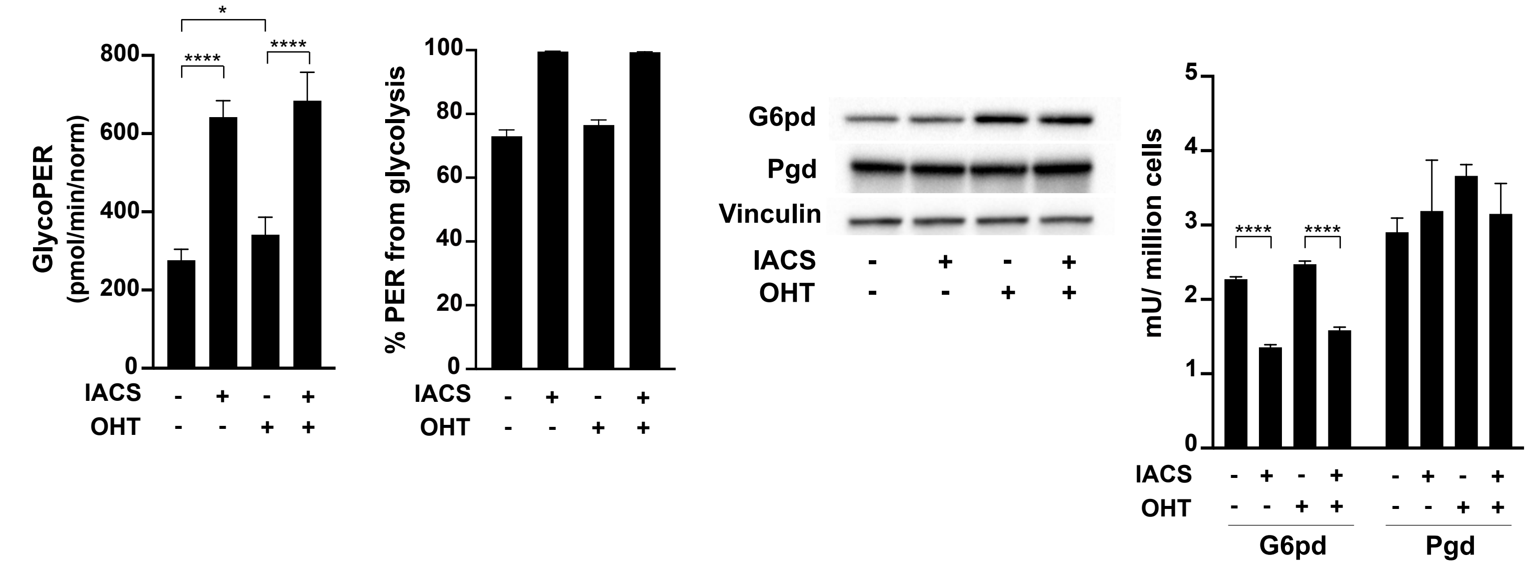

E

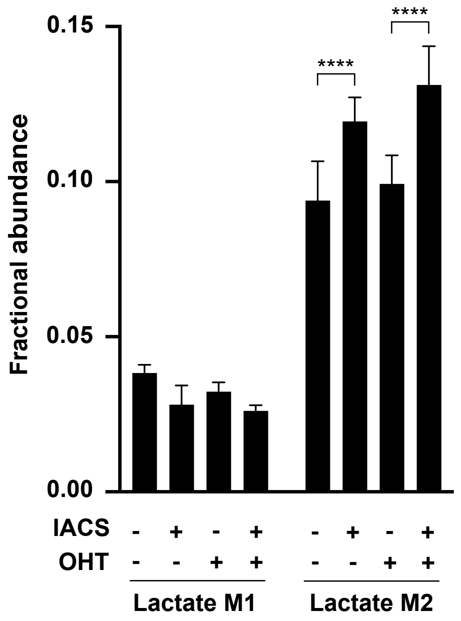

F

	two-way ANOVA	DF	SS	MS	F	p-value
Fig. 3E	Between treatments	7	5916.9	845.27	16.45	5.52E-10
	IACS	3	4423.4	1474.47	28.70	4.57E-10
	DHEA	1	624.5	624.5	12.16	0.0012
	Interaction	3	869.0	289.67	5.64	0.0025
	Error	40	2055.0	51375		
	IACS selectivity for OHT	1	251.72	251.72	4.90	0.033
Fig. 3F	Between treatments	7	9018	1288	36	2.61E-15
	IACS	3	5369	1790	50	1.38E-13
	6AN	1	1978	1978	55	4.78E-09
	Interaction	3	1671	557	16	7.49E-07
	Error	40	1434	36		
	IACS selectivity for OHT	1	407	407	11	1.68E-03
Fig. 3G	Between treatments	7	39001.4	5571.63	106.27	8.39E-24
	IACS	3	11905.98	3968.7	75.70	1.53E-16
	2DG	1	19043.14	19043.14	363.21	1.12E-21
	Interaction	3	8052.3	2684.1	51.19	1.14E-08
	Error	40	2097.2	52.43		
	IACS selectivity for OHT	1	2.215	2.215	0.0422	0.838

G

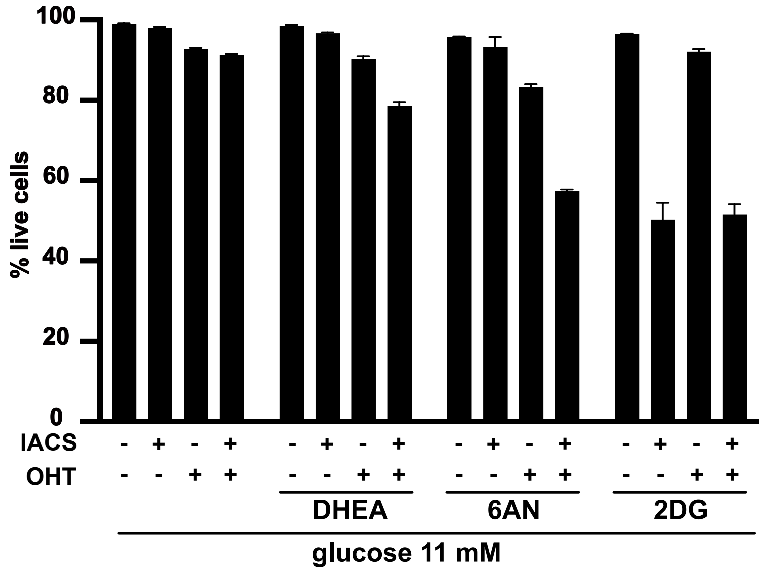

H

	$F_{1,8}$	p-value
a	2661	2.21E–11
b	5177	1.55E–12
c	327	9.00E–08
d	140	2.42E–06

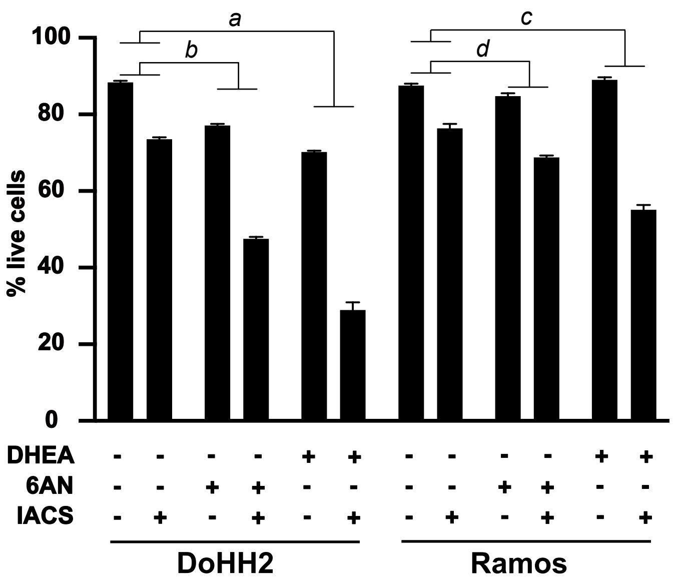

J

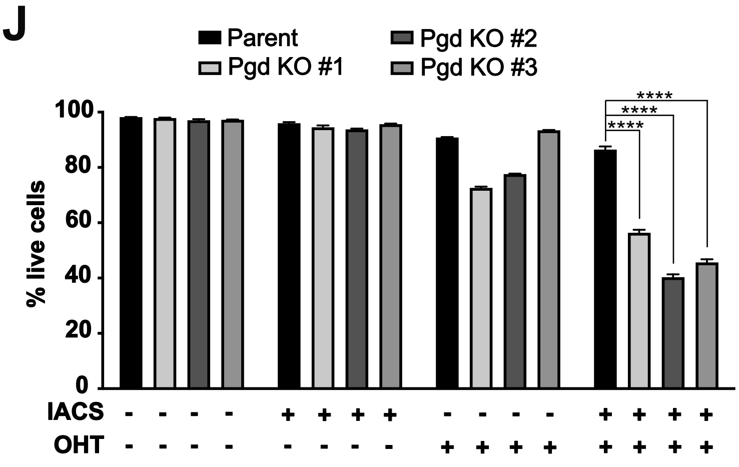

I

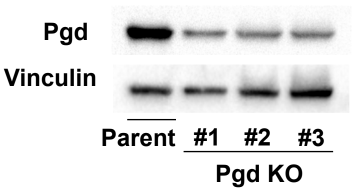

Appendix Figure S3. The pentose phosphate pathway protects MYC-overexpressing cells from IACS-010759-induced toxicity. (**A**) Basal glycolytic rate (glycoPER), calculated from the proton efflux rate (PER) of FL^{MycER} cells, primed with 100 nM OHT (48h) and/or treated with 135 nM IACS-010759 for 24 hours, as indicated. * $p \leq 0.05$; **** $p \leq 0.0001$. (**B**) Percentage of PER from glycolysis (as opposed to mitochondrial respiration) on the same samples shown in (A). (**C**) Immunoblot analysis and (**D**) Enzymatic activity assays for G6pd and Pgd after 24 hours of IACS-010759 treatment. (**E**) Fractional abundance of ^{13}C-labeled lactate, the endpoint of the anaerobic glycolytic pathway, after addition of [1,2-^{13}C]glucose to FL^{MycER} cells treated as in (A). (**F**) Two-way ANOVA analysis of the data shown in Figure 3E, 3F and 3G, as indicated. A test for the selective cytotoxicity of IACS-010759 toward OHT-treated FL^{MycER} cells is reported in red at the end of each section for the co-treatments with DHEA, 6AN and 2DG, respectively. (**G**) Cell viability FL^{MycER} cells grown in medium containing 11 mM glucose after 40 hours of IACS-010759 treatment in the presence of 50 µM dehydroepiandrosterone (DHEA), 10 µM 6- aminonicotinamide (6AN) or 1 mM 2-deoxyglucose (2DG). Error bars: SD (n=3). (**H**) Immunoblot on lysates from parental FL^{MycER} cells and clones derived after CRISPR-Cas9 mediated *Pgd* gene knockout. (**I**) Cell viability of FL^{MycER} cells and the Pgd KO clones shown in (H) primed with OHT and treated for 40h with 135 nM IACS-010759. Error bars: SD (n=3). (**J**) Cell viability of DoHH2 and Ramos lymphoma cells treated with 135 nM IACS-010759 (40h), either alone, with 50 µM DHEA, or with 10 µM 6AN. Error bars: SD (n=3). The table below the graph shows the two-way ANOVA analysis of IACS-010759-induced cell death in the presence and absence of either DHEA or 6AN.

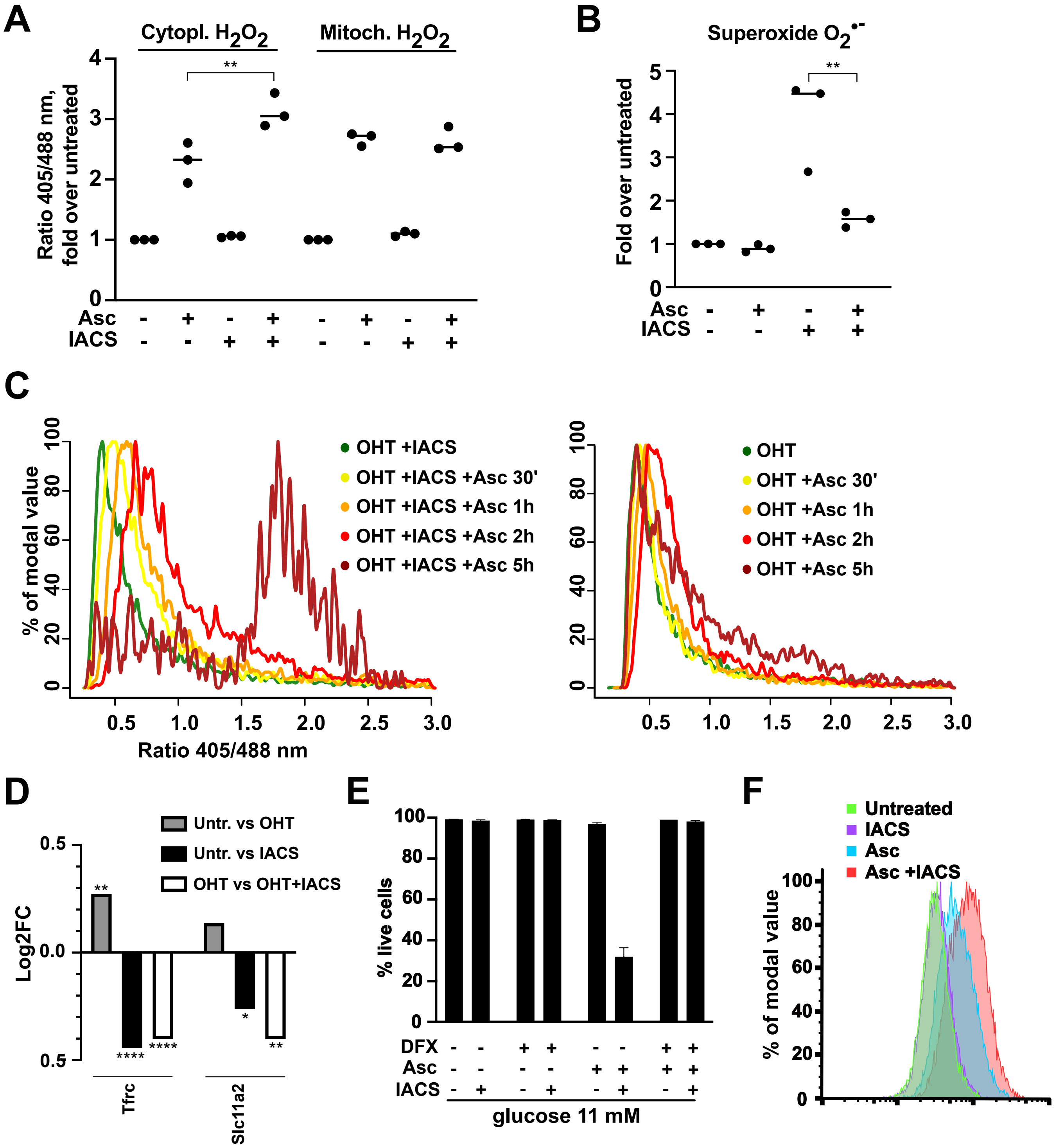

Appendix Figure S4. Ascorbate potentiates IACS-010759-induced cell death by increasing oxidative stress. (**A**) H_2O_2 quantification, expressed as fold-increase of the 405/488 nm fluorescence ratio over untreated of either the cytoplasmic (left) or mitochondrial (right) roGFP2-ORP1 biosensor from FL^{MycER} cells treated with 135 nM IACS-010759 (IACS) for 24h and/or 400 µM ascorbate (Asc) for 3h. ** $p \leq 0.01$. (**B**) dihydroethidium staining quantification of superoxide anion $O_2^{\bullet-}$ production in FL^{MycER} cells, based on. * $p \leq 0.05$. Each dot in the graphs in **A** and **B** is from an independent biological replicate and represents the average of thousands of events (single cells) in a distinct cell population, normalized to the untreated condition. (**C**) As Figure 4B, in OHT-primed cells. (**D**) Log fold change (Log2FC) of the iron-regulated mRNAs *Tfrc* and *Slc11a2* in FL^{MycER} cells with 135 nM IACS-010759, with or without pre-treatment with OHT, based on our previous RNA-seq profiles (Donati *et al.*, 2022), with the following q-values: * $q \leq 0.05$; ** $q \leq 0.01$; **** $q \leq 0.0001$. (**E**) Cell viability of FL5.12 cells grown in medium containing 11 mM glucose at the end of treatment (48h IACS-010759, 6h Asc), in the presence or absence of 50 µM deferoxamine (DFX, added 1 hour before Asc). Error bars: SD (n=3). (**F**) Representative flow cytometry of lipid peroxides quantification by BODIPY C11 staining of FL5.12 cells treated with 135 nM IACS for 24h and/or 400 µM Asc for 3h where indicated (as in Figure 4D).

A

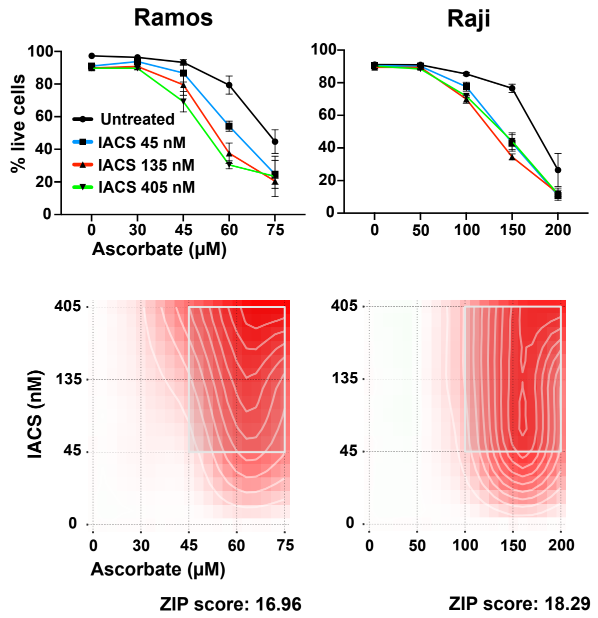

B

	B cell neoplasm	COO	CCC	*MYC* locus
**DoHH2**	DHL	GCB		transl.
**SU-DHL-6**	DHL	GCB	BCR	transl.
**Karpas 422**	DLBCL	GCB	OxPhos	*
**HBL-1**	DLBCL	ABC	BCR	wt
**Ramos**	BL			transl.
**Raji**	BL			transl.

C

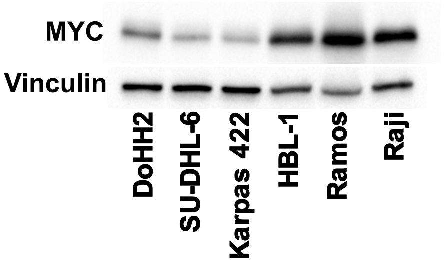

Appendix Figure S5. Ascorbate potentiates IACS-010759-induced cell death by increasing oxidative stress. (**A**) The Burkitt lymphoma cell lines Raji and Ramos were treated with the indicated concentrations of IACS-010759 and ascorbate, and cell viability determined after 24 hours. Error bars: SD (n=3). Top: live cell counts; bottom: drug interaction landscapes and synergy scores, calculated according to the ZIP model. Classification (**B**) and MYC protein levels (**C**) in the B-cell lymphoma cell lines used in this work. The table in (B) shows the B-cell neoplasm of origin, COO and CCC subtype classification (Caro *et al*, 2012; Devin *et al*, 2019; Polo *et al*, 2007; Reddy *et al*, 2017), and the status of the *MYC* locus (transl.: translocated). The status of the *MYC* locus in Karpas 422 (*) remains uncertain, having been described as either wt or rearranged (Deng *et al*, 2018; Dyer *et al*, 1990; Farrugia *et al*, 1994); nonetheless these cells express MYC protein levels comparable to those of MYC-rearranged, DLBCL lines DoHH2 and SU-DHL-6.
