# Supplementary material for: Oxidative stress enhances the therapeutic action of a respiratory inhibitor in MYC‐driven lymphoma
Source: EMBO Mol Med. 2023 May 9;15(6):e16910. doi: 10.15252/emmm.202216910 (PMC10245039; doi:10.15252/emmm.202216910)
Supplement: Supplementary file 2 — Movie EV1 [file EMMM-15-e16910-s003.zip › Movie EV1 legend.pdf]

Movie EV1. Related to Figure 2E. Time-lapse microscopy on FL^{MycER} cells expressing the Grx1-roGFP2 biosensor, following treatment with OHT and IACS-010759. Dead cells were identified by Propidium iodide staining (shown in grayscale). The 405/488 nm fluorescence ratio was visualized based on the false color heatmap shown below.

Ratio 405/488 nm:

0 1.5
