# Supplementary figures and images for: Oxidative stress enhances the therapeutic action of a respiratory inhibitor in MYC‐driven lymphoma
Source: EMBO Mol Med. 2023 May 9;15(6):e16910. doi: 10.15252/emmm.202216910 (PMC10245039; doi:10.15252/emmm.202216910)

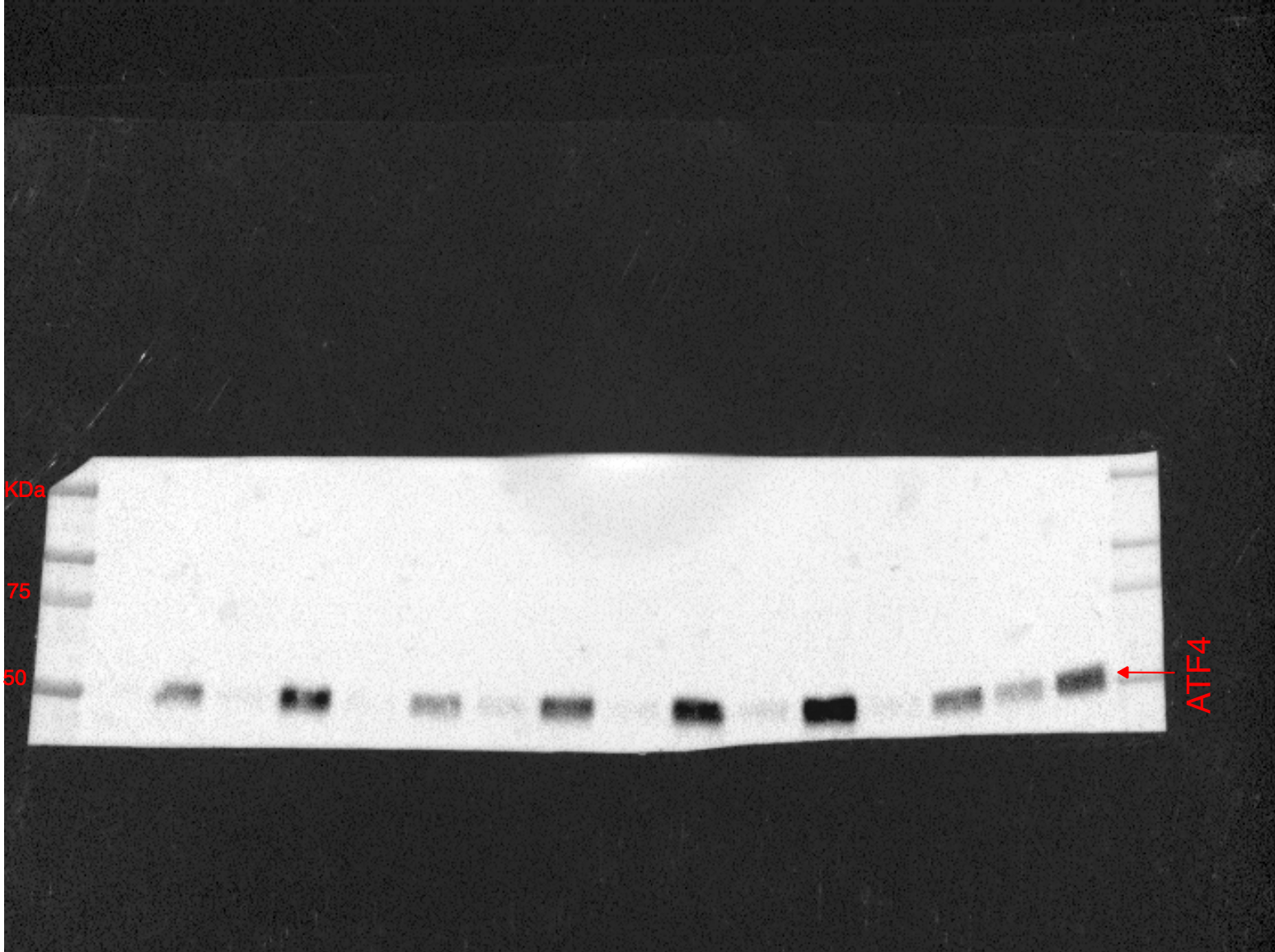

Supplement: Supplementary file 4 — Source Data for Figure 2 [file EMMM-15-e16910-s005.zip › 2F/2F_ATF4.tif]

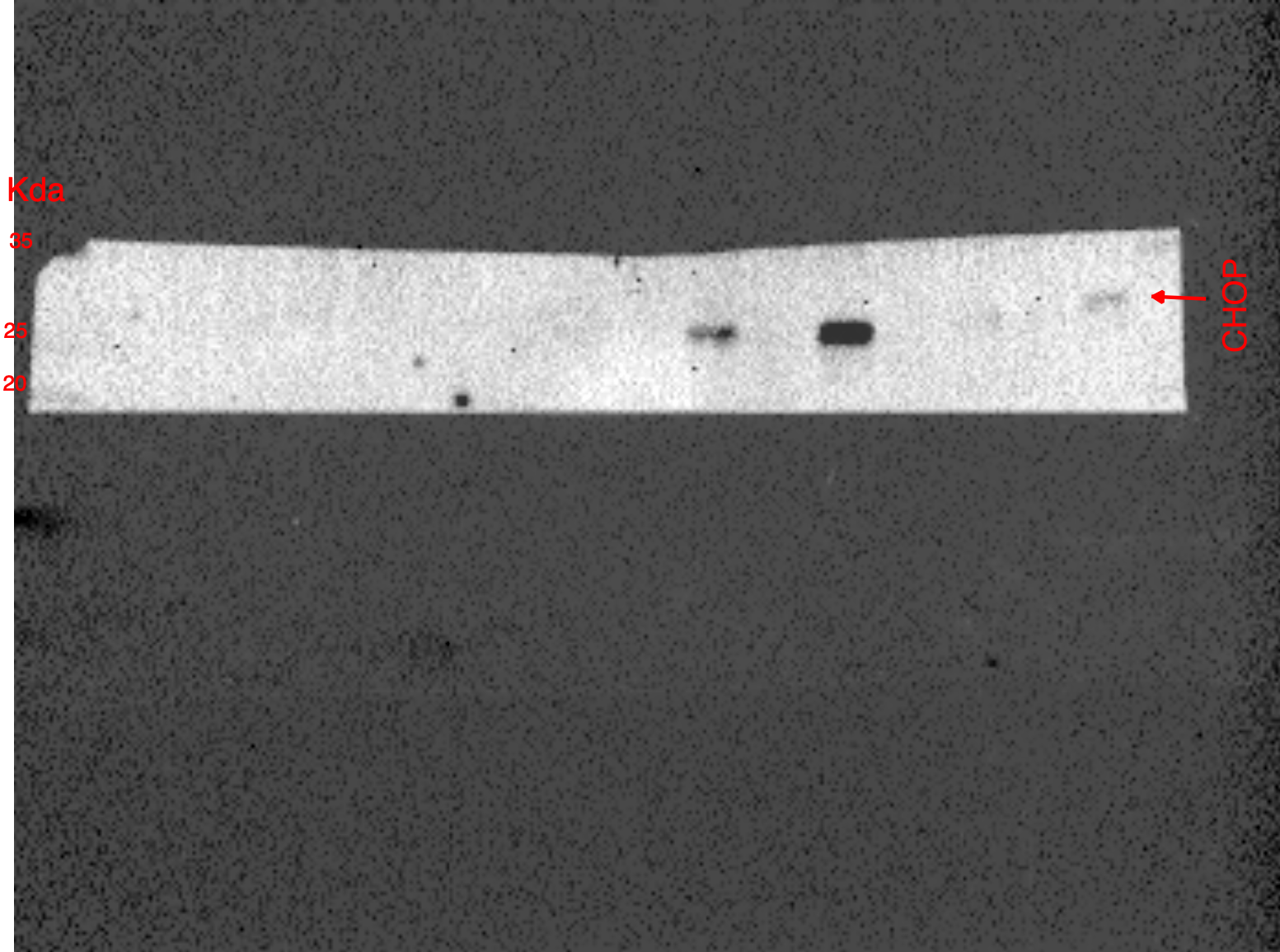

Supplement: Supplementary file 4 — Source Data for Figure 2 [file EMMM-15-e16910-s005.zip › 2F/2F_CHOP.tif]

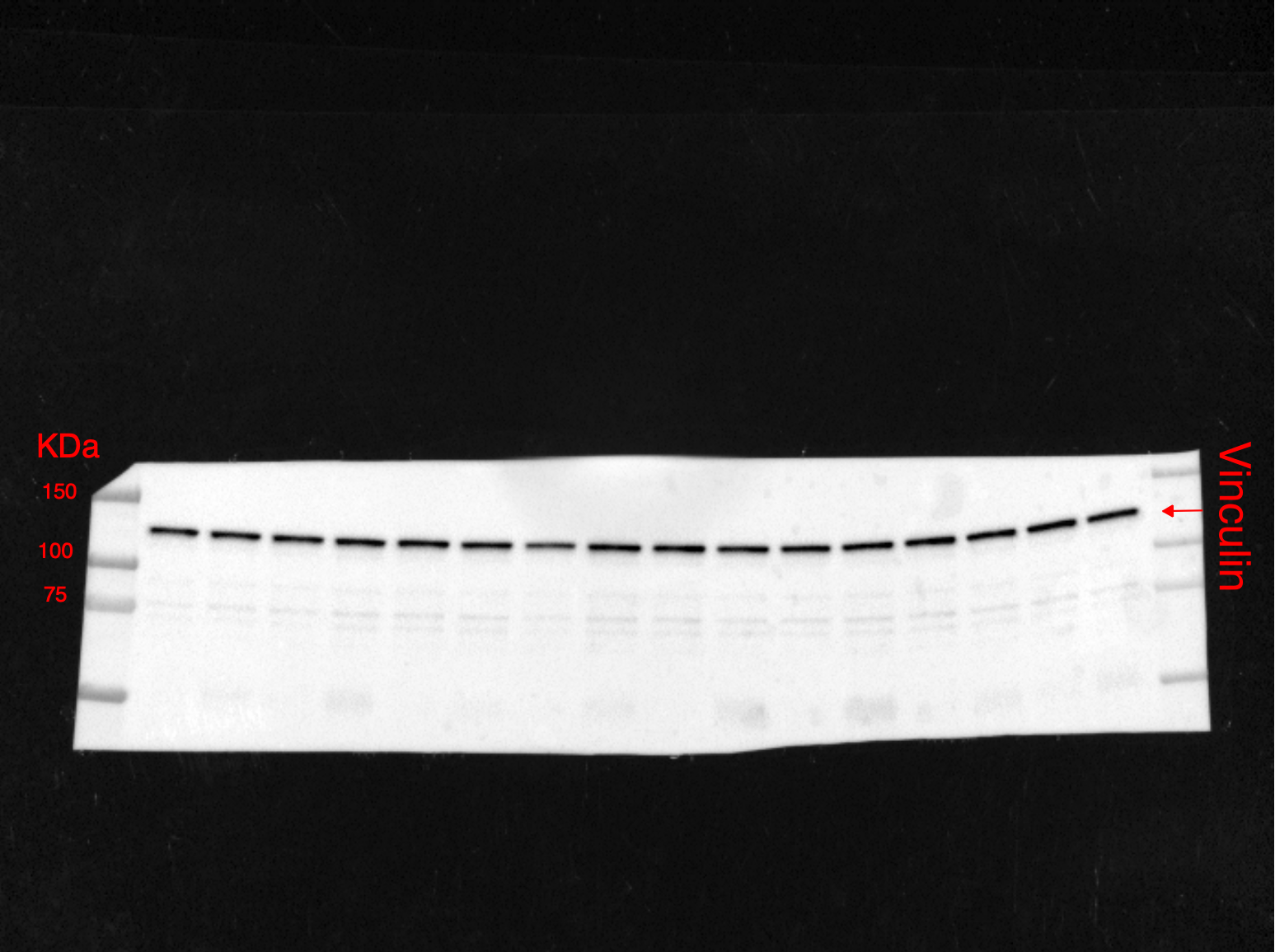

Supplement: Supplementary file 4 — Source Data for Figure 2 [file EMMM-15-e16910-s005.zip › 2F/2F_Vinculin.tif]
