# Supplementary material for: Oxidative stress enhances the therapeutic action of a respiratory inhibitor in MYC‐driven lymphoma
Source: EMBO Mol Med. 2023 May 9;15(6):e16910. doi: 10.15252/emmm.202216910 (PMC10245039; doi:10.15252/emmm.202216910)
Supplement: Supplementary file 9 [file EMMM-15-e16910-s006.pdf]

EMBO Molecular Medicine

Oxidative stress enhances the therapeutic action of a respiratory inhibitor in MYC-driven lymphoma

Bruno Amati, Giulio Donati, Paola Nicoli, Alessandro Verrecchia, Veronica Vallelonga, Ottavio Croci, Simona Rodighiero, Matteo Audano, Laura Cassina, Aya Ghsein, Giorgio Binelli, Alessandra Boletta, and Nico Mitro
DOI: 10.15252/emmm.202216910

Corresponding author(s): Bruno Amati (bruno.amati@ieo.it) , Giulio Donati (giulio.donati@ieo.it)

Review Timeline:

Submission Date:	19th Sep 22
Editorial Decision:	18th Oct 22
Revision Received:	13th Mar 23
Editorial Decision:	4th Apr 23
Revision Received:	13th Apr 23
Accepted:	21st Apr 23

Editor: Jingyi Hou / Zeljko Durdevic

Transaction Report:

(Note: With the exception of the correction of typographical or spelling errors that could be a source of ambiguity, letters and reports are not edited. Depending on transfer agreements, referee reports obtained elsewhere may or may not be included in this compilation. Referee reports are anonymous unless the Referee chooses to sign their reports.)

18th Oct 2022

Dear Dr. Amati,

Thank you again for submitting your work to EMBO Molecular Medicine. We have now heard back from three referees who agreed to evaluate your manuscript. As you will see from the reports below, the referees acknowledge the potential interest of the study. However, they raise a series of concerns, which we would ask you to address in a major revision of the manuscript.

I think that the referees' recommendations are relatively straightforward, so there is no need to reiterate their comments. In particular, Referee #2 was concerned that most of the experiments were performed in an in vitro context, and we would ask you to strengthen the in vivo relevance for at least some of the key findings. Referee #3' major comments #4 and #5 need to be carefully addressed.

We would welcome the submission of a revised version within three months for further consideration. Please note that EMBO Molecular Medicine in principle only allows a single round of revision. As acceptance or rejection of the manuscript will depend on another round of review, your responses should be as complete as possible.

EMBO Molecular Medicine has a "scooping protection" policy, whereby similar findings that are published by others during review or revision are not a criterion for rejection. Should you decide to submit a revised version, I do ask that you get in touch after three months if you have not completed it to update us on the status.

We are aware that many laboratories cannot function at full efficiency during the current COVID-19/SARS-CoV-2 pandemic and have therefore extended our "scooping protection policy" to cover the period required for a full revision to address the experimental issues. Please let me know should you need additional time, and also if you see a paper with related content published elsewhere.

Please read below for important editorial formatting and consult our author's guidelines for proper formatting of your revised article for EMBO Molecular Medicine.

I look forward to receiving your revised manuscript.

Sincerely,
Jingyi

Jingyi Hou
Editor
EMBO Molecular Medicine

*****

When submitting your revised manuscript, please carefully review the instructions that follow below. We perform an initial quality control of all revised manuscripts before re-review; failure to include requested items will delay the evaluation of your revision.

We require:

1) A .docx formatted version of the manuscript text (including legends for main figures, EV figures and tables). Please make sure that the changes are highlighted to be clearly visible.

2) Individual production quality figure files as .eps, .tif, .jpg (one file per figure). For guidance, download the 'Figure Guide PDF': (https://www.embopress.org/page/journal/17574684/authorguide#figureformat).

3) A .docx formatted letter INCLUDING the reviewers' reports and your detailed point-by-point responses to their comments. As part of the EMBO Press transparent editorial process, the point-by-point response is part of the Review Process File (RPF), which will be published alongside your paper.

4) A complete author checklist, which you can download from our author guidelines

(https://www.embopress.org/page/journal/17574684/authorguide#submissionofrevisions). Please insert information in the checklist that is also reflected in the manuscript. The completed author checklist will also be part of the RPF.

5) Please note that all corresponding authors are required to supply an ORCID ID for their name upon submission of a revised manuscript.

6) It is mandatory to include a 'Data Availability' section after the Materials and Methods. Before submitting your revision, primary datasets produced in this study need to be deposited in an appropriate public database, and the accession numbers and database listed under 'Data Availability'. Please remember to provide a reviewer password if the datasets are not yet public (see https://www.embopress.org/page/journal/17574684/authorguide#dataavailability).

In case you have no data that requires deposition in a public database, please state so in this section. Note that the Data Availability Section is restricted to new primary data that are part of this study.

7) For data quantification: please specify the name of the statistical test used to generate error bars and P values, the number (n) of independent experiments (specify technical or biological replicates) underlying each data point and the test used to calculate p-values in each figure legend. The figure legends should contain a basic description of n, P and the test applied. Graphs must include a description of the bars and the error bars (s.d., s.e.m.). See also 'Figure Legend' guidelines: https://www.embopress.org/page/journal/17574684/authorguide#figureformat

8) At EMBO Press we ask authors to provide source data for the main and expanded view figures. Our source data coordinator will contact you to discuss which figure panels we would need source data for and will also provide you with helpful tips on how to upload and organize the files.

9) Our journal encourages inclusion of *data citations in the reference list* to directly cite datasets that were re-used and obtained from public databases. Data citations in the article text are distinct from normal bibliographical citations and should directly link to the database records from which the data can be accessed. In the main text, data citations are formatted as follows: "Data ref: Smith et al, 2001" or "Data ref: NCBI Sequence Read Archive PRJNA342805, 2017". In the Reference list, data citations must be labeled with "[DATASET]". A data reference must provide the database name, accession number/identifiers and a resolvable link to the landing page from which the data can be accessed at the end of the reference. Further instructions are available at .

10) We replaced Supplementary Information with Expanded View (EV) Figures and Tables that are collapsible/expandable online. A maximum of 5 EV Figures can be typeset. EV Figures should be cited as 'Figure EV1, Figure EV2" etc... in the text and their respective legends should be included in the main text after the legends of regular figures.

- For the figures that you do NOT wish to display as Expanded View figures, they should be bundled together with their legends in a single PDF file called *Appendix*, which should start with a short Table of Content. Appendix figures should be referred to in the main text as: "Appendix Figure S1, Appendix Figure S2" etc.

- Additional Tables/Datasets should be labeled and referred to as Table EV1, Dataset EV1, etc. Legends have to be provided in a separate tab in case of .xls files. Alternatively, the legend can be supplied as a separate text file (README) and zipped together with the Table/Dataset file.

See detailed instructions here:

.

11) The paper explained: EMBO Molecular Medicine articles are accompanied by a summary of the articles to emphasize the major findings in the paper and their medical implications for the non-specialist reader. Please provide a draft summary of your article highlighting

- the medical issue you are addressing,

- the results obtained and

- their clinical impact.

This may be edited to ensure that readers understand the significance and context of the research. Please refer to any of our published articles for an example.

12) For more information: There is space at the end of each article to list relevant web links for further consultation by our readers. Could you identify some relevant ones and provide such information as well? Some examples are patient associations,

relevant databases, OMIM/proteins/genes links, author's websites, etc...

13) Author contributions: You will be asked to provide CRediT (Contributor Role Taxonomy) terms in the submission system. These replace a narrative author contribution section in the manuscript.

14) A Conflict of Interest statement should be provided in the main text.

15) Every published paper now includes a 'Synopsis' to further enhance discoverability. Synopses are displayed on the journal webpage and are freely accessible to all readers. They include a short stand first (maximum of 300 characters, including space) as well as 2-5 one-sentences bullet points that summarizes the paper. Please write the bullet points to summarize the key NEW findings. They should be designed to be complementary to the abstract - i.e. not repeat the same text. We encourage inclusion of key acronyms and quantitative information (maximum of 30 words / bullet point). Please use the passive voice. Please attach these in a separate file or send them by email, we will incorporate them accordingly.

Please also suggest a striking image or visual abstract to illustrate your article as a PNG file 550 px wide x 300-600 px high.

EMBO Molecular Medicine has a "scooping protection" policy, whereby similar findings that are published by others during review or revision are not a criterion for rejection. Should you decide to submit a revised version, I do ask that you get in touch after three months if you have not completed it, to update us on the status.

Please note: When submitting your revision you will be prompted to enter your funding and payment information. This will allow Wiley to send you a quote for the article processing charge (APC) in case of acceptance. This quote takes into account any reduction or fee waivers that you may be eligible for. Authors do not need to pay any fees before their manuscript is accepted and transferred to the publisher.

EMBO Press participates in many Publish and Read agreements that allow authors to publish Open Access with reduced/no publication charges. Check your eligibility: https://authorservices.wiley.com/author-resources/Journal-Authors/open-access/affiliation-policies-payments/index.html

***** Reviewer's comments *****

Referee #1 (Comments on Novelty/Model System for Author):

The data is quite compelling, with regards to the effectiveness of the drug combinations. These drug combinations were designed through a rationalities approach, which is a strength of the manuscript. However, a lingering problem is that the initial experiments, and the main theme of the manuscript, stress the idea that these combinations were meant to target cells with MYC deregulation and with and OXPHOS metabolic switch. The final result would suggest that the outcomes may very well be MYC-independent, and more widespread or inespecific.

Referee #1 (Remarks for Author):

In this manuscript, Donati et al. investigate the molecular mechanisms that underlie the synthetic lethal interaction between MYC deregulation and IACS-010759, a specific inhibitor of the electron transport chain (ETC) complex I. These studies refine a previous report by this same group (Donati et al, Mol Oncol 2022; 16).

The authors combine in vitro studies, xenografts and metabolic analyses to discover that IACS+MYC deregulation disrupt redox homeostasis, sensitizing cells to the action of pro-oxidant drugs, like ascorbate. The combination of IACS and ascorbate proves to be very effective at killing different B cell non-Hodgkin lymphoma cell lines in vitro, and also in xenograft studies, which are also extended to two different, primary derived xenografts of double-hit lymphoma. These studies refine a previous report by this same group (Donati et al, Mol Oncol 2022; 16).

MYC deregulation is thought to cause important metabolic changes in cells, some of which represent new dependencies. These metabolic dependencies could be used as points of entry for new therapeutic strategies. Exploiting these vulnerabilities offers an opportunity to treat MYC-driven cancers, and particularly those that are refractory to current therapies, like certain aggressive B cell lymphomas (i.e. double-hit lymphomas). Such unmet medical need makes new potential therapeutic combinations, like the one proposed in this manuscript, of particular interest.

The results of this study show that IACS-010759 increases oxidative stress by disrupting redox homeostasis. This response is in part compensated in cells overexpressing MYC by shuttling glucose toward the generation of NADPH via the pentose phosphate pathway. Inhibiting this pathway with specific drugs, or using vitamin C (ascorbate, a pro-oxidant), tilts the balance and reduces cell viability. These observations are all supported by a thorough and careful study on metabolites and experiments that take advantage of selective compounds that enhance of relieve this cellular response (e.g. NAC, BSO). Overall, the message is that

MYC deregulation sensitizes cells to the inhibition of oxidative phosphorylation by different means. Links between MYC activity and oxidative stress were previously reported, and the current manuscript is consistent with this idea.

The (perhaps) most compelling piece of data is the fact that a rational (data-based) combination of ascorbate and IACS-010759 effectively kills B-cell non-Hodgkin lymphoma cell lines (aggressive Burkitt and Diffuse Large cell lymphoma) and patient-derived xenografts. The process by which authors dissect the pathway and reason the use of specific drug combinations is a strength of this study. The tumor study results are quite relevant, because of their clinical potential. But conceptually, what seems lost as the data flow, is the connection to MYC deregulation. The IACS+Ascorbate combination seems to kill everything, and despite the results in the FL5.12 MycER cell line, which are in some cases subtle, there is no strong evidence to say for sure that this is something specific to cells with MYC deregulation. So maybe the message of the manuscript, and the title, should be mindful of that.

Some additional pints would also warrant revision.

Major comments:

1- Metabolic changes equivalent to the ones proposed here upon MYC deregulation also occur in normal B cells upon activation with cytokines+/-BCR crosslink (metabolism is normally rewired toward aerobic glycolysis). A prediction is that the combination of IACS+ascorbate could be also toxic to normal B cells in this setting. The xenograft experiments using immunodeficient mice cannot assess potential toxicities in normal B cell counterparts, which may be relevant if this drug combination were to advance to preclinical studies.

2- The authors imply a connection between OXPHOS and the response to the different drug combinations, but this is not really sustained in the cell line data, in part because of the lack of metabolic data on all the cell lines used in these experiments. Only Karpas 422 would fit the OxPHOS category described by Shipp and cols in Monti et al, Blood 2005, but the synergistic effect of the IACS+Ascorbate combination in this cell line is perhaps the less compelling (Fig. 5A). Other cell lines used here belong to different gene expression groups and the evidence for an OXPHOS metabolic makeup is less clear. The two double-hit PDX models are also of unclear metabolic profile. Although the authors showed in the past that OXPHOS and MYC expression seem to positively correlate, it is a bit of a stretch to assume that this happens in all cases.

3- Fig 6C, D: In these PDX xenograft studies, the size of the error bars does not seem to support the existence of statistically significant differences, particularly for DFBL-69487. An alternative statistical test, different from a One-Way ANOVA comparing endpoints (day 14), may help resolve this dicordance.

4- The changes in Nrf2 levels shown in Fig.1 are somewhat subtle. One can infer that the authors are using Nrf2 here as an indirect reporter of oxidative stress, but this is a MYC target (also mentioned in the text) that may be important to help cells cope with oxidative stress. The fact that IACS reduces Nrf2 levels would suggest some kind of mechanistic connection. Does depletion of Nrf2 by genetic means (e.g. RNAi) alter cell survival in cell with MYC deregulation? Does IACS add anything to that response?

5- Fig 1D: the increase in superoxide levels in response to IACS is variegated (2 outliers, 2 replicates with much lower response). Were these independent experiments? Maybe a larger number of replicates would help discern if the response is or not homogeneous, and if there are any true outliers here.

6- Figure 5A and Suppl Fig 4 would indicate that ascorbate at relatively high doses is toxic to most of the cell lines tested, except for FL5.12 cells. There is some additional toxicity provided by the combination with IACS, but most of the effect seems to be driven by ascorbate. However, this effect is lost in vivo, at least from what can be inferred from the xenograft studies. Any reason for this discrepancy? Is this technical?

7- Fig. 4A: The graph shows that in absence of ascorbate (=0), cell viability is already reduced in the IACS+4-OHT group (to about 70%). While this is expected, the authors mention that they used 135nM IACS, which, looking at the IACS only curve, doesn't seem to have any effect on viability. This would suggest some effect of MYC deregulation, which is not reflected in the text. Also, the slope of the IACS+4-OHT curve is different from the IACS only one, suggesting some kind of interaction. Can the authors discuss this more carefully?

Minor comments:

8- The changes in cell viability in all experiments, and particularly when combining MYC deregulation (4-OHT) and IACS seem to have limited penetrance, this is, only kill a fraction of cells. Any idea why this is the case? Do longer times lead to further reductions in viability, or are there always a fraction of cells not affected?

9- Some studies seem to indicate that tamoxifen can promote oxidative stress in different cell types, e.g. in murine hepatocytes and also in breast cancer cells (e.g. Nazarewicz et al, Cancer Res 2007; 67(3)). Potential off target effects could be addressed

by including controls with FL5.12 parental cells (without MYC-ER) exposed to 4-OHT.

10- Page 7: looks like when mentioning Supplementary Fig 2A, C, the authors may have been referring to Suppl. Fig 2 C,D.

11- Lactate M1 is not shown in the scheme in Fig 3A

Referee #2 (Comments on Novelty/Model System for Author):

I find the exclusive use of various compounds (of unknown selectivity) to interrogate biology quite limiting. key conclusions should be tested with more defined genetic tools.

Experiments are technically well done.

Novelty is limited by many previous studies on this compound from this group and others.

The medical impact as it stands today is limited, to my knowledge the compound has not advanced to the clinic.

Model systems. Most of the study is done in vitro, this is limiting with respect to tumor metabolism. Only the final conclusion (Vit C and IACS) is tested in vivo. I am not a metabolism expert, but this would raise concerns for me.

Referee #2 (Remarks for Author):

The authors use a series of pharmacological single agent and combination studies to show that MYC expressing lymphoma cell lines are sensitive to a ETC I inhibitor (IACS010759) and that various (more or less characterized) compounds that purport to target glucose metabolism, or redox signals alter sensitivity to the IACS compound. The study builds on a series of previous studies by this group and others on the ETC 1 inhibitory compound, but it appears to pursue a new angle related to redox and the role of Vit. C in drug synergy through the production of ROS.

I would be curious to learn more about the compound and its selectivity and the introduction should provide a better explanation. I would also be curious to learn more about the selective activity against MYC expressing tumor cells. Does MYC directly increase ETC 1 activity? Does MYC increase expression or (ribosomal?) translation of the ETC1 proteins?

Experimentally, the study is interesting although somewhat minimal and only the final conclusion (synergy of IACS and Vit C) has been tested in more than one cell line or in vivo.

Given the uncertain specific of compounds that are used to elicit biological effect, the study would be improved if key data were supported using genetic studies: E.g., the study relies heavily on compounds that directly/indirectly influence ROS biology (NAC, VitC, PPP inhibition etc). the conclusions would be strengthened by experiments that show effects of NRF2 activation or inhibition (e.g. by modulating Keap1 levels).

The drug synergy shown in Figure 4 seems modest (or the presentation is hard to interpret). A more intuitive comparison of IC50 data might be more convincing. The in vivo synergy data showing tumor volumes look good. I am less sure about the imaging studies.

Some results remain speculative and inconclusive. E.g., compounds (of unclear specificity) are used to block the PPP shunt. This will alter glucose levels and increase glycolysis may bypass the ETC1 inhibition. Alternatively, blocking PPP will affect NADPH and redox potential of cells. What is the relevant role of these effects in cells in vitro and in tumor model (physiological glucose) in vivo?

Referee #3 (Comments on Novelty/Model System for Author):

Note to editor: this is a rather fundamental paper, some aspects require more rigorous data, and the direct medical impact cannot be predicted as yet (impossible question in my opinion)

Referee #3 (Remarks for Author):

Donati et al employ an inducible system for expresion of Myc in a B cell line to investigate the synthetic lethality between Myc overexpression and inhibition of ETC complex I by IACS-010759 - a compound currently in clincial trials.
They report that MYC hyperactivation and ETC inhibition disrupt redox homeostasis, leading to oxidative stress and apoptosis. By combining IACS-010759 with pro-oxidant drugs such as ascorbate, the efficacy can be further enhanced.

The work is a direct extension of a related recent paper by the group (Donati et al Mol Oncol 2022) where Myc overexpression combined with Bcl2 inhibition were investigated. The current work complements the previous paper by showing that Myc makes cells vulnerable for oxidative stress, which is in itself not a novel observation. The authors do provide novel mechanistic insight in order to make this paper novel and relevant. In addition, though all mechanistic experiments are done with just 1 genetically manipulated cell line, authors do make the transition to additional B cell lines and patient derived PDX in Figure 6, which is commendable.
Nevertheless several aspects should be improved and/or clarified to make the article more coherent and impactful.

Major comments
1. Figure 1B: This essential WB data require quantification of multiple experiments, as the change in Nrf2 levels in the nucleus is not clear. Why does OHT+IACS not result in increased Nrf2, if the signature of Nrf2 is present regardless of IACS+/-? In addition protein markers/ kD should be indicated, especially as there is controversy on the size of Nrf2 by western blot.
2. Figure 2 E: in the text is mentioned there is an abrupt increase of 405/488. If t0 is defined per each cell, then many of them have this abrupt increase much before (2h) they die. Can these differences between cells be explained/discussed?
3. Figure 4: Which kind of ROS does ascorbate generate? Are IACS and ascorbate inducing two ROS generating pathways, or do they generate the same kind of ROS species but at higher levels? It would be informative to perfom H2O2 and superoxide measurements as in Fig. 1 C and 2 with the 2 compounds + combination.
4. The role of glucose in the culture medium is important in determining the effects of IACS - as presented in the previous paper. Authors should make more clear why and what is new here in relation to glucose. The current article should be an independent body of work; proposed is to show new data on the role of glucose concentration in media and subsequent effects of IACS, ascorbate etc. - see also next point.
5. Since ferric iron chelator deferoxamine (DFX) fully prevented cell death in double-treated cells (Figure 4C), the question arise to what extent the processes studied may involve a ferroptotic component. IS there synergy with ferroptosis inducers/prevention by ferroptosis inhibitors? What is the role of GPX4? In Discussion authors make some assumptions about that, suggesting future investigations, but these aspects should be included here, to increase significance and impact.

Minor comments
1. Fig 1A Which part of these data is new and which was already published? In the previous paper Donati, 2022 also RNAseq analysis of OHT-treated versus not-treated are shown but a diferent time point. In there only the 1st 3 pathways are shown and Nrf2 does not appear. Please explain.
2. Fig3C graph ap;pears to be missing, only one representative histogram is shown.
3. The synergy between Myc, IACS and ascorbate in Figures 5 and S4; these figures are of too low resolution.
4. Figure 6; Y-axes in B, D do not have a legend.

EMM-2022-16910

Donati et al.

Oxidative stress enhances the therapeutic action of a respiratory inhibitor in MYC-driven lymphoma

<u>Authors' Response</u>

We thank the Referees for their constructive comments, which have significantly helped us improving the impact and clarity of our manuscript. Our point-by-point rebuttal and a description of the changes implemented in our work are provided below. For full information of the Referees, we include here some additional experiments performed during the revision process (Figure R1).

Referee #1 (Comments on Novelty/Model System for Author):

The data is quite compelling, with regards to the effectiveness of the drug combinations. These drug combinations were designed through a rationalities approach, which is a strength of the manuscript. However, a lingering problem is that the initial experiments, and the main theme of the manuscript, stress the idea that these combinations were meant to target cells with MYC deregulation and with and OXPHOS metabolic switch.

We previously reported that OxPhos- and MYC-related transcriptional programs are closely correlated in DLBCL, and that MYC hyperactivation sensitizes cells to the OxPhos inhibitor IACS-010759 (Donati *et al*, 2022). Here, we clarify that this MYC-dependent sensitization to IACS-010759 depends on cooperation in causing oxidative stress, and not on increased OxPhos metabolic dependency. In fact, most of our mechanistic experiments were performed in FL^{MycER} cells, which maintain a glycolytic energy metabolism regardless of exogenous MYC activation. We have amended our text to clarify this concept: "independently from OHT treatment, energy production in FL^{MycER} cells was mainly glycolytic (Supplemental Figure 3B)".

The final result would suggest that the outcomes may very well be MYC-independent, and more widespread or inespecific.

The oxidative stress induced by IACS-010759 is indeed independent of MycER activation, however, we clearly show that MYC hyperactivation causes additional oxidative stress (Fig. 1B, Fig. 2A), which sensitizes cells to pharmacological disruption of redox homeostasis (Fig. 2D, Fig. 3E, F, Fig. 4A).

Referee #1 (Remarks for Author):

In this manuscript, Donati et al. investigate the molecular mechanisms that underlie the synthetic lethal interaction between MYC deregulation and IACS-010759, a specific inhibitor of the electron transport chain (ETC) complex I. These studies refine a previous report by this same group (Donati et al, Mol Oncol 2022; 16).

The authors combine in vitro studies, xenografts and metabolic analyses to discover that IACS+MYC deregulation disrupt redox homeostasis, sensitizing cells to the action of pro-oxidant drugs, like

ascorbate. The combination of IACS and ascorbate proves to be very effective at killing different B cell non-Hodgkin lymphoma cell lines in vitro, and also in xenograft studies, which are also extended to two different, primary derived xenografts of double-hit lymphoma. These studies refine a previous report by this same group (Donati et al, Mol Oncol 2022; 16).

MYC deregulation is thought to cause important metabolic changes in cells, some of which represent new dependencies. These metabolic dependencies could be used as points of entry for new therapeutic strategies. Exploiting these vulnerabilities offers an opportunity to treat MYC-driven cancers, and particularly those that are refractory to current therapies, like certain aggressive B cell lymphomas (i.e. double-hit lymphomas). Such unmet medical need makes new potential therapeutic combinations, like the one proposed in this manuscript, of particular interest.

The results of this study show that IACS-010759 increases oxidative stress by disrupting redox homeostasis. This response is in part compensated in cells overexpressing MYC by shuttling glucose toward the generation of NADPH via the pentose phosphate pathway. Inhibiting this pathway with specific drugs, or using vitamin C (ascorbate, a pro-oxidant), tilts the balance and reduces cell viability. These observations are all supported by a thorough and careful study on metabolites and experiments that take advantage of selective compounds that enhance of relieve this cellular response (e.g. NAC, BSO). Overall, the message is that MYC deregulation sensitizes cells to the inhibition of oxidative phosphorylation by different means. Links between MYC activity and oxidative stress were previously reported, and the current manuscript is consistent with this idea.

It is true that previous reports established the links between MYC and oxidative stress: our manuscript fully acknowledges these previous links and – as also noted by the Referee in the next paragraph – builds upon this concept to pinpoint an important therapeutic mechanism-of-action.

The (perhaps) most compelling piece of data is the fact that a rational (data-based) combination of ascorbate and IACS-010759 effectively kills B-cell non-Hodgkin lymphoma cell lines (aggressive Burkitt and Diffuse Large cell lymphoma) and patient-derived xenografts. The process by which authors dissect the pathway and reason the use of specific drug combinations is a strength of this study. The tumor study results are quite relevant, because of their clinical potential. But conceptually, what seems lost as the data flow, is the connection to MYC deregulation. The IACS+Ascorbate combination seems to kill everything, and despite the results in the FL5.12 MycER cell line, which are in some cases subtle, there is no strong evidence to say for sure that this is something specific to cells with MYC deregulation. So maybe the message of the manuscript, and the title, should be mindful of that.

The sensitizing effects of MYC activation in FL^{MycER} cells shown in our original submission (formerly Fig. 4A) were indeed somewhat subtle, yet fully reproducible. To illustrate this effect in a clearer manner, the experiment was repeated using a shorter time of ascorbate treatment (6h instead of 12h). Moreover, in addition to FL^{MycER} we used BaF^{MycER}, a distinct B-cell line that was also described in our previous work (Donati *et al.*, 2022). The new results clearly show increased effectiveness of the IACS/ascorbate treatment in OHT-induced cells (Fig. 4A).

Moreover, the Referee is right in noting that IACS-010759-induced sensitization to the pro-oxidant activity of ascorbate is not strictly dependent on MYC hyperactivation. This is now explicitly noted in our Results section: “In FL^{MycER} cells, in which a broader concentration range of ascorbate was tested, the highest concentrations of this vitamin allowed killing by IACS-010759 in the absence of OHT priming.” However, this is precisely where the added relevance to MYC-driven tumors lays, based on the mechanistic aspects reported in our work. Briefly here:

(i.) Killing by IACS is clearly potentiated by MYC overexpression, as reported in our previous work (Donati *et al.*, 2022), and confirmed here.

(ii.) In this work, we demonstrate that this is due to the combined oxidative stress induced by MYC and IACS.

(iii.) Ascorbate exploits this mechanism to further strengthen IACS' anti-tumoral activity.

Altogether, the title of our manuscript appropriately conveys the therapeutic potential of this drug combination to treat MYC-overexpressing B-cell lymphomas.

Some additional pints would also warrant revision.

Major comments:

1- Metabolic changes equivalent to the ones proposed here upon MYC deregulation also occur in normal B cells upon activation with cytokines+/-BCR crosslink (metabolism is normally rewired toward aerobic glycolysis). A prediction is that the combination of IACS+ascorbate could be also toxic to normal B cells in this setting. The xenograft experiments using immunodeficient mice cannot assess potential toxicities in normal B cell counterparts, which may be relevant if this drug combination were to advance to preclinical studies.

The Referee is correct in pointing this out, and we have now added a paragraph covering this issue in our Discussion: "Similar to what observed after ectopic MycER activation (Donati *et al.*, 2022) (Supplemental Fig. 3A), mitogenic stimulation of B-cells coordinately potentiates glycolysis and mitochondrial respiration (e.g. Caro-Maldonado *et al*, 2014) as well as ROS production (Wheeler & Defranco, 2012). Thus, we cannot a priori exclude that a pro-oxidant therapeutic regimen such as IACS-010759 and ascorbate may be toxic for activated B-cells. However, we note that high-dose ascorbate has already proven safe and tolerable in a clinical setting, either alone or in association with platinum-based and other ROS-producing chemotherapeutic agents (Bottger *et al*, 2021). Moreover, high-dose ascorbate reinforced anti-cancer immunotherapy in multiple solid tumor models (Magri *et al*, 2020), implying that it does not impair – or rather may favor – anti-cancer immunity: it will be of high interest to address whether the same may be true in combination with IACS-010759 or other mitochondrial inhibitors."

We shall also emphasize here that, while treatment of our experimental animals with IACS-010759 and ascorbate yielded no overall toxicity, the potential effects on normal activated B-cells (or other activated cell types) are a common caveat of effective anti-cancer therapies. As also outline by Referee 3, "direct medical impact cannot be predicted as yet (impossible question in my opinion)" and is objectively beyond the scope of the present pre-clinical study.

2- The authors imply a connection between OXPHOS and the response to the different drug combinations, but this is not really sustained in the cell line data, in part because of the lack of metabolic data on all the cell lines used in these experiments. Only Karpas 422 would fit the OxPHOS category described by Shipp and cols in Monti et al, Blood 2005, but the synergistic effect of the IACS+Ascorbate combination in this cell line is perhaps the less compelling (Fig. 5A). Other cell lines used here belong to different gene expression groups and the evidence for an OXPHOS metabolic makeup is less clear. The two double-hit PDX models are also of unclear metabolic profile. Although the authors showed in the past that OXPHOS and MYC expression seem to positively correlate, it is a bit of a stretch to assume that this happens in all cases.

This is an important point to be clarified, and we thank the Referee for bringing it up. Indeed, as we shall explain if further detail below, the relevance of our findings goes beyond the metabolic classification of tumors as “OxPhos”.

Our previous data unraveled the sensitization of MYC overexpressing cells to two distinct mitochondrial inhibitors, tigecycline (D'Andrea *et al*, 2016; Ravà *et al*, 2018) and IACS-010759 (Donati *et al.*, 2022). These results, together with the positive correlation between MYC- and OxPhos-associated gene expression signatures in DLBCL patient datasets (Donati *et al.*, 2022) led us to the hypothesis that MYC-overexpressing lymphomas were more dependent on proficient OxPhos, and thus could be selectively targeted with mitochondrial inhibitor-based therapies. However, here we present observations clarifying that MYC-driven sensitization to mitochondrial inhibitors is not due to increased reliance on OxPhos for energy production, but rather to increased sensitivity to the oxidative stress caused by these drugs.

Consistent with the above, the synergistic anti-cancer activity of IACS and ascorbate is not limited to the OxPhos CCC subtype, which has been associated with reliance upon OxPhos as main energy source (Caro *et al*, 2012). Accordingly, we had already stated in the Introduction that “this mechanism does not strictly depend on the reliance of tumor cells upon OxPhos, and can be exploited to further enhance killing of MYC-overexpressing cells by combining IACS-010759 with other pro-oxidant drugs”. In the Results, we conclude: “In summary, IACS-010759 and ascorbate synergized in vitro to kill MYC-overexpressing mature B-cell neoplasms, regardless of their origin and molecular subtype”. Then again, in the Discussion: “In the present work, we clarify that the MYC-mediated sensitization to IACS-010759 is brought about by a critical accumulation of oxidative stress, rather than increased reliance on OxPhos for energy metabolism” followed by “MYC-induced sensitization to IACS-010759 did not depend upon OxPhos-driven ATP production, as was instead the case for IACS-010759 mediated killing of glycolysis-deficient cells (Molina *et al.*, 2018)” and finally “This combination also showed synergy in BL and DLBCL lymphoma cell lines of multiple molecular subtypes, not restricted to the “OxPhos” category (Supplemental Figure 5B).”

3- Fig 6C, D: In these PDX xenograft studies, the size of the error bars does not seem to support the existence of statistically significant differences, particularly for DFBL-69487. An alternative statistical test, different from a One-Way ANOVA comparing endpoints (day 14), may help resolve this dicordance.

The Referee states that “the size of the error bars does not seem to support the existence of statistically significant differences”. Please note that, as indicated in the legend, the bars represent the standard deviation (SD), not the standard error (SE) of the sample. There is no discordance between the graph and the result of the one-way ANOVA, which remains the most powerful test available to detect significant differences between means.

In the Results, we have now added the following “Similar effects were obtained with two DHL-derived patient-derived xenografts (PDX) (Townsend *et al*, 2016), injected systemically in NSG mice and monitored by whole-body bioluminescence (Figure 6C, D). Note that one of the PDX tumors, PDX-69487, showed a remarkable resistance to IACS-010759 alone even if used at a higher dose; nonetheless, as with all other xenografts, the combination did cause a significant reduction in tumor growth relative to untreated controls.”

Altogether, our data fully support the conclusions given in the text for the response to ascorbate and/or IACS-010759, for both cell line- and PDX-based xenografts (Figure 6).

4- The changes in Nrf2 levels shown in Fig.1 are somewhat subtle. One can infer that the authors are using Nrf2 here as an indirect reporter of oxidative stress, but this is a MYC target (also mentioned in the text) that may be important to help cells cope with oxidative stress. The fact that IACS reduces Nrf2 levels would suggest some kind of mechanistic connection. Does depletion of Nrf2 by genetic means (e.g. RNAi) alter cell survival in cell with MYC deregulation? Does IACS add anything to that response?

To clarify the role of Nrf2 in modulating the response to the oxidative stress linked to MycER activation and IACS treatment, we employed CRISPR-Cas9 engineering on our FL^{MycER} cells and derived KO clones lacking either Nrf2 or its negative regulator Keap1. While these experiments allowed us to critically reassess the quality of the anti-Nrf2 antibody used in our original submission, they did not provide the additional molecular or phenotypic insight that would have formally been needed for inclusion in our revised manuscript. The data are included here for the Referees (Figure R1) and described in the following two subsections:

4A – NRF2 IMMUNOBLOTTING:

The immunoblot performed to confirm Nrf2 ablation in Nrf2 KO clones (Fig. R1A) revealed a non-specific band recognized by the antibody (clone D1Z9C, Cell Signaling Technology). This non-specific band is predominant and runs very close to the real Nrf2 band, which is essentially undetectable in our cells without prior treatment with the proteasome inhibitor MG132 (Fig. R1A). Most problematically, the two bands fail to be reproducibly resolved in most SDS-PAGE gels (Fig. R1B, top). Given this problem with clone D1Z9C, we probed our blots with anti-Nrf2 polyclonal antibody (PA5-27882, ThermoFisher), with which we could confirm increased Nrf2 levels in MG132-treated cells (Fig. R1B), as well as in Keap1 KO clones, as compared to untreated parental cells (Fig. R1C).

We conclude that the immunoblot presented in Fig. 1B of our original manuscript (shown again here: Fig. R1D, top) cannot be trusted to represent a specific Nrf2 signal. Moreover, immunoblotting of equivalent subcellular fractions from OHT- and IACS-treated FL^{MycER} cells with the polyclonal anti-Nrf2 antibody did not detect a specific signal above background noise, not even upon prolonged exposures (Fig. R1D, bottom).

On a formal basis, given the above results, we conclude that immunoblotting on cell fractions is not a reliable means to monitor Nrf2 activity. We thus removed this experiment (formerly Fig. 1B) from our manuscript. We are truly thankful to the Referees for having prompted us to produce the information that led to this decision. Most importantly, this impacts in no way on the initial observation made on our work, namely the identification of the Nrf2-mediated oxidative stress response as the top OHT-responsive pathway in FL^{MycER} cells (Figure 1A). All the experiments that followed from this observation remain fully valid.

4B – PHENOTYPIC ANALYSIS OF NRF2 AND KEAP1 KO CLONES:

Having derived Nrf2 and Keap1 knockout (KO) FL^{MycER} clones, we addressed the response of those cells to OHT and IACS treatment.

Six Nrf2 KO clones were tested (Fig. R1E), revealing incongruent changes, with drug sensitivities ranging from reduced (KO #1, #2) to unchanged (KO #3, #6) to increased (KO #4, #5) relative to parental cells. We surmise that clonal variability predominated over the effects of Nfr2 loss in those clones. Of note here, KO efficiencies did not allow us to work with polyclonal populations,

and transduction of FL^{MycER} cells with Nrf2 shRNAs achieved only partial knockdown, precluding this strategy as an alternative to the KO. In conclusion, we did not find definitive evidence for an involvement of the Nrf2 pathway in cell survival after MYC-activation and/or IACS-010759 treatment and thus decided to only show the activation of the Nrf2 pathway as an indirect readout of oxidative stress.

Unlike for Nrf2 KO, phenotypic assessment of 4 Keap1 KO FL^{MycER} clones consistently showed increased resistance to IACS-010759 (Fig. R1F). A possible explanation for the apparent contradiction between the results obtained with Nrf2 and Keap1 KO could be the existence of non-canonical, Nrf2-independent functions of Keap1 (Kopacz *et al*, 2020). Specifically, while the Keap1-Nrf2 pathway responds to moderate oxidative stress, the Keap1-Pgam5 pathway is activated by heavy oxidative damage to induce oxeiptosis, a ROS-induced mitochondrial pathway of cell death (Holze *et al*, 2018). The eventual involvement of this mechanism in the effects of IACS-010759 is an intriguing possibility, but way too preliminary to make a formal point here, and must therefore be the subject of detailed studies.

5- Fig 1D: the increase in superoxide levels in response to IACS is variegated (2 outliers, 2 replicates with much lower response). Were these independent experiments? Maybe a larger number of replicates would help discern if the response is or not homogeneous, and if there are any true outliers here.

The superoxide values shown in Fig. 1C (1D in the previous version) are indeed from independent experiments. The same is true for H2O2 measurements in Fig. 1B. This is now fully clarified in the legend, "Each point in the graphs in **B** and **C** is from an independent biological replicate, each representing the average of thousands of events (single cells) in a distinct cell population, normalized to the untreated condition".

Even though the relative increase of superoxide level induced by IACS is different among the biological replicates, a consistent induction of this ROS species is evident in all of them, with no significant impact of OHT. Similar results and variability for IACS treated cells were obtained in subsequent experiments aimed at quantifying $O2^{\bullet -}$ induced by IACS and ascorbate (Supplemental Fig. 4B).

6- Figure 5A and Suppl Fig 4 would indicate that ascorbate at relatively high doses is toxic to most of the cell lines tested, except for FL5.12 cells. There is some additional toxicity provided by the combination with IACS, but most of the effect seems to be driven by ascorbate. However, this effect is lost in vivo, at least from what can be inferred from the xenograft studies. Any reason for this discrepancy? Is this technical?

These in vitro assays (now in Fig. 5A and Suppl. Fig. 5A) demonstrate synergistic interactions between ascorbate and IACS in defined concentration ranges in all the cell lines tested, regardless of their differential cytotoxic activity as single agents. It is therefore inexact to deduce, as done here by the Referee, that "most of the effect seems to be driven by ascorbate". We have amended our description in the Results, in order to better emphasize this concept: "… ascorbate also increased IACS-010759 mediated killing in these cells, with the two drugs displaying significant synergistic effects within defined concentration ranges (Figure 5A, B)".

Regarding the in vivo results, the daily dose of ascorbate (4 g/kg) was initially selected from a published protocol (Chen *et al*, 2008) and was maintained as it did not show any obvious toxic effect for the animals. While the effective local concentrations reached in our in vivo experiments remain to be determines, the fact that ascorbate enhances the anti-tumoral activity of IACS-

010759 is conclusively demonstrated in our four tumor models (Fig. 6A-D). Altogether, there is no discrepancy here: our in vivo and in vitro data with IACS and ascorbate are consistent, and fully support the conclusions drawn in the text.

7- Fig. 4A: The graph shows that in absence of ascorbate (=0), cell viability is already reduced in the IACS+4-OHT group (to about 70%). While this is expected, the authors mention that they used 135nM IACS, which, looking at the IACS only curve, doesn't seem to have any effect on viability. This would suggest some effect of MYC deregulation, which is not reflected in the text. Also, the slope of the IACS+4-OHT curve is different from the IACS only one, suggesting some kind of interaction. Can the authors discuss this more carefully?

Detailed comments on this figure were provided above under the general remarks. We shall add here that the conditional toxicity of IACS-010759 after exogenous MYC activation in FL^{MycER} cells was documented in our previous study (Donati *et al.*, 2022) and is fully consistent with the data reported here: no killing by IACS alone, but partial killing (at this particular concentration) in OHT-treated cells.

We hypothesized that the observed difference in the slope of the viability curves between IACS and OHT+IACS in Fig. 4A might result from excessive oxidative stress induced by ascorbate at higher concentrations and at a relatively late time point (12h), which eventually exceeded that induced by IACS and overwhelmed cellular redox defenses. This experiment was repeated at a shorter time point (6h) in 2 different MycER-expressing cell lines: as discussed above, the new results showed consistently increased toxicity for the combination in OHT-primed cells (Fig. 4A).

Minor comments:

8- The changes in cell viability in all experiments, and particularly when combining MYC deregulation (4-OHT) and IACS seem to have limited penetrance, this is, only kill a fraction of cells. Any idea why this is the case? Do longer times lead to further reductions in viability, or are there always a fraction of cells not affected?

"Limited penetrance" seems a somewhat inappropriate concept here: the point is that enhanced cell killing, even if quantified as a partial effect over a defined period of time (as inherent to any viability measurement), may be sufficient to achieve significant anti-tumoral effects if it supersedes cell proliferation. Eventually, preclinical in vivo data are the only means inform of the potential therapeutic window provided by a given drug combination, as clearly confirmed in our work for IACS and ascorbate.

9- Some studies seem to indicate that tamoxifen can promote oxidative stress in different cell types, e.g. in murine hepatocytes and also in breast cancer cells (e.g. Nazarewicz et al, Cancer Res 2007; 67(3)). Potential off target effects could be addressed by including controls with FL5.12 parental cells (without MYC-ER) exposed to 4-OHT.

This control was provided in our previous study (Donati *et al.*, 2022), where we showed that OHT priming sensitized FL^{MycER} but not parental FL5.12 cells to killing by IACS-010759. Hence, the on-target action of OHT was established, and does not need to be re-addressed here.

10- Page 7: looks like when mentioning Supplementary Fig 2A, C, the authors may have been referring to Suppl. Fig 2 C,D.

This is true and has been corrected in the text (now Suppl. Fig. 3). We thank the Referee for spotting the mistake.

11- Lactate M1 is not shown in the scheme in Fig 3A

What is shown in Fig. 3A is a schematic summary of the relevant glucose metabolic pathways, not the tracing experiment. We acknowledge that as originally written in our text, this was prone to confusion, and thank the Referee for pointing this out. We have now rewritten the text as follows: "This decreased PPP flux would also be expected to suppress the production of lactate from glucose passing through the PPP before re-entering glycolysis (Figure 3A), measurable as lactate M1 in our tracing experiment: while apparent in our data, this effect remained below statistical significance (Supplemental Figure 3E)."

Referee #2 (Comments on Novelty/Model System for Author):

I find the exclusive use of various compounds (of unknown selectivity) to interrogate biology quite limiting. key conclusions should be tested with more defined genetic tools.

This concern is addressed below.

Experiments are technically well done.

Novelty is limited by many previous studies on this compound from this group and others.

We must firmly disagree with the notion that the novelty of our work is limited by previous studies. Instead, our findings significantly extend these studies, making new and important points in the field:

It is true that anti-cancer response to OxPhos inhibition with IACS-010759 has been described in multiple papers, including our own regarding sensitization by oncogenic MYC (Donati *et al.*, 2022). Nonetheless, we would like to point out the novelty of our findings regarding the disruption of redox equilibrium as the major mechanism of action for of IACS-010759, as well as the role of this mechanism in MYC-induced sensitization. Within this work, we have also exploited these findings for the rational design of a new combinatorial therapy (i.e. IACS-010759 + ascorbate) in MYC-associated lymphomas.

The medical impact as it stands today is limited, to my knowledge the compound has not advanced to the clinic.

The Referee is right in pointing this out, and this is precisely why we deem our findings timely and important from a clinical standpoint. Altogether, our data make a strong case for the combination of drugs targeting the ETC (of which IACS-010759 can be taken as a paradigm here) with ascorbate or other drugs that potentiate killing of tumor cells with a mechanism-driven rationale.

Considering the specific case of IACS-010759, our work offers new perspectives that may guide further clinical developments. This is a key aspect, which is developed in a dedicated paragraph in our Discussion: "The combinatorial action of IACS-010759 and ascorbate unraveled here might prove to be relevant in diverse clinical settings. First, etc..."

Model systems. Most of the study is done in vitro, this is limiting with respect to tumor metabolism. Only the final conclusion (Vit C and IACS) is tested in vivo. I am not a metabolism expert, but this would raise concerns for me.

We understand and agree with the concerns regarding anti-cancer mechanisms in vivo. Indeed, we had already tried to assess oxidative damage by immunohistochemical analysis of specific biomarkers, including 8-hydroxydeoxyguanosine and 4-hydroxynonenal. These experiments did not yield conclusive results, owing mainly to the non-quantitative nature of the assay. More detailed in vivo studies (such as metabolic profiling, etc…) are not readily accessible, and beyond the scope of the present study.

This notwithstanding, we would like to point out that the pro-oxidant in vivo effects of high-dose ascorbate are well documented in the literature, as mentione in our text: "Parenteral administration of a high dose of ascorbate (vitamin C) has been shown to have pro-oxidant and anti-cancer activity in preclinical models, etc…". Finally, the same objection could be made regarding the specific inhibition of mitochondrial complex I by IACS-010759 in vitro (Donati *et al.*, 2022; Molina *et al*, 2018), for which no tractable in vivo biomarker was described so far.

Referee #2 (Remarks for Author):

The authors use a series of pharmacological single agent and combination studies to show that MYC expressing lymphoma cell lines are sensitive to a ETC I inhibitor (IACS010759) and that various (more or less characterized) compounds that purport to target glucose metabolism, or redox signals alter sensitivity to the IACS compound. The study builds on a series of previous studies by this group and others on the ETC 1 inhibitory compound, but it appears to pursue a new angle related to redox and the role of Vit. C in drug synergy through the production of ROS.

I would be curious to learn more about the compound and its selectivity and the introduction should provide a better explanation.

As mentioned in the Introduction, and as rigorously characterized in the original report (Molina *et al.*, 2018), IACS-010759 is a specific inhibitor of ETC complex I. There is really not much else to be explained about this, and documenting details of the previous study would be beyond the scope in our text.

Would there be formal reasons to suspect off-target effects or alternative targets of the compound behind the biological effects described in our work, this would of course become a key point of our discussion. However, there are no such alternative targets documented, nor do we have any reason to suspect their existence based on the available data. While this type of considerations will always be relevant in pharmacological studies, raising them here without a formal reason to do so would provide no added value to our study.

I would also be curious to learn more about the selective activity against MYC expressing tumor cells.

This was documented in detail in our previous study (Donati *et al.*, 2022): as written in our Introduction this had shown that "a specific inhibitor of ETC complex I, IACS-010759 (Molina et al., 2018), selectively killed MYC-overexpressing cells by inducing intrinsic apoptosis (Donati et al., 2022)". Indeed, that was the basis for the follow-up study presented here.

Does MYC directly increase ETC 1 activity? Does MYC increase expression or (ribosomal?) translation of the ETC1 proteins?

There is ample evidence in the literature for the importance of MYC in promoting mitochondrial gene expression and biogenesis. We have added a sentence to clarify this at the beginning of the second paragraph in our Introduction:

"Multiple studies linked MYC to mitochondrial biogenesis and activity (Li *et al*, 2005; Morrish & Hockenbery, 2014; Wolpaw & Dang, 2018), in particular via activation of nuclear genes encoding the mitochondrial RNA polymerase POLRMT (Oran *et al*, 2016) or mitochondrial ribosomal proteins (D'Andrea *et al.*, 2016), leading to enhanced respiratory activity (Donati *et al.*, 2022)". Etc…

This is a relevant point to be made in as background information in our work, and the thank the Referee from bringing it up. Yet, we believe that reviewing this aspect in further detail is unnecessary.

Experimentally, the study is interesting although somewhat minimal and only the final conclusion (synergy of IACS and Vit C) has been tested in more than one cell line or in vivo.

As mentioned in our reply to Referee #1, besides FL^{MycER} cells we now present data in a second B-cell line BaF^{MycER}. The new results clearly confirm the effects of ascorbate in reinforcing IACS-induced killing (Fig. 4A). For further detail, please refer to our reply to Referee #1.

The above notwithstanding, we must disagree with the Referee on the "somewhat minimal" nature of the in vitro data included in our original submission. In fact, besides the data in FL^{MycER} cells (and now also BaF^{MycER}), some of the key pharmacological interactions were also demonstrated in the DoHH2 and Ramos lymphoma cell lines. In particular:

i. Our data in in FL^{MycER} cells showed that (quoting our test) "the inhibitor of GSH synthesis buthionine sulfoximine (BSO) enhanced killing (Figure 2D). This effect of BSO in potentiating the cytotoxic action of IACS-010759 was confirmed in two MYC-rearranged human lymphoma cell lines, DoHH2 and Ramos, derived from a double-hit and a Burkitt's lymphoma (BL), respectively (Supplemental Figure 2A)."
ii. "To confirm the importance of the oxidative PPP for the selective killing of MYC-overexpressing cells by IACS-010759, we inhibited G6pd and Pgd (Figure 3A) with dehydroepiandrosterone (DHEA) and 6-aminonicotinamide (6AN), respectively" etc…
iii. "Finally, either DHEA or 6AN also potentiated killing by IACS-010759 in human MYC-rearranged lymphoma cells lines (Supplemental Figure 3H).

Concerning the in vivo studies, we have the same comment here as written above in reply to Referee 1:

We understand and agree with the concerns regarding anti-cancer mechanisms in vivo. Indeed, we had already tried to assess oxidative damage by immunohistochemical analysis of specific biomarkers, including 8-hydroxydeoxyguanosine and 4-hydroxynonenal. These experiments did not yield conclusive results, owing mainly to the non-quantitative nature of the assay. More detailed in vivo studies (such as metabolic profiling, etc…) are not readily accessible, and beyond the scope of the present study.

Given the uncertain specific of compounds that are used to elicit biological effect, the study would be improved if key data were supported using genetic studies: E.g., the study relies heavily on

compounds that directly/indirectly influence ROS biology (NAC, VitC, PPP inhibition etc). the conclusions would be strengthened by experiments that show effects of NRF2 activation or inhibition (e.g. by modulating Keap1 levels).

We agree that genetic models can provide important evidence against off-target and other confounding effects that may affect pharmacological studies. Indeed, to confirm the involvement of the PPP pathway, we targeted Pgd, as described in our text: "We then sought to confirm these results in a genetic model of PPP impairment obtained by ablation of *Pgd* through CRISPR-Cas9 targeting. Of note, all of the Pgd KO FL^{MycER} clones obtained were heterozygous, with residual Pgd protein expression (Supplemental Figure 3I), consistent with the essential nature of this gene, as defined in the Broad Institute Dependency Map (DepMap) portal (Ghandi *et al*, 2019). This notwithstanding, these *Pgd*-targeted clones showed increased sensitivity to IACS-010759 following OHT priming (Supplemental Figure 3J)".

The above notwithstanding, we would like to emphasize here the compelling nature of our pharmacological data. In particular, the use of different pharmacological treatments to modulate the cells' antioxidant defenses (see Fig. 2 and 3) concordantly pointed to oxidative stress as the mediator of IACS-010759 selective cytotoxic activity, makes it highly unlikely that the results shown are products of off-target effects of any single drug.

Regarding the role of the Nrf2-Keap1 pathway, we targeted the Nrf2 and Keap1 loci in FL^{MycER} cells and addressed response of the resulting KO clones to MycER activation and IACS treatment (Fig. R1E, F). Please refer to our detailed response to Referee #1 (point 4) for a description of these results.

The drug synergy shown in Figure 4 seems modest (or the presentation is hard to interpret). A more intuitive comparison of IC50 data might be more convincing.

The figure has been updated with new data obtained with a shorter ascorbate treatment (6h instead of 12h), which more convincingly show increased sensitivity to the IACS/ascorbate combination after MycER activation (Fig. 4A). Moreover, the results were also reproduced in BaF^{MycER} cells.

The in vivo synergy data showing tumor volumes look good. I am less sure about the imaging studies.

In vivo luminescence provides a quantitative measure of tumor load, provided as bilateral femur radiant efficiency (Fig. 6, legend; Materials and methods), and differences among treatment groups were assessed with the most appropriate statistical test (see also answer to Referee #1, point 3). We thus see no reason to question the relevance of the effects reported by imaging of the PDX-derived tumors (Fig. 6C, D), given also their consistency with those scored by subcutaneous tumor volume with the lymphoma cell lines Ramos and DoHH2 (Fig. 6A, B).

Some results remain speculative and inconclusive. E.g., compounds (of unclear specificity) are used to block the PPP shunt. This will alter glucose levels and increase glycolysis may bypass the ETC1 inhibition. Alternatively, blocking PPP will affect NADPH and redox potential of cells. What is the relevant role of these effects in cells in vitro and in tumor model (physiological glucose) in vivo?

While we reiterate the improbability of incurring in off-target effects from different drugs, all coherently pointing toward redox homeostasis as critical for the cytotoxic action of IACS in MYC overexpressing cells, we now provide a genetic validation for our model. Specifically, FL^{MycER}

clones where the *Pgd* gene was ablated showed increased sensitivity to IACS treatment after OHT-priming (Suppl. Fig. 3J).

Regarding the first scenario outlined here by the Referee, one would expect that increased compensatory glycolysis due to PPP inhibition would lead to increased resistance to IACS-010759 after MycER activation, but our results show that the opposite is true. In fact, PPP inhibition leads to increased killing by IACS-010759 in these cells, owing to disruption of redox homeostasis (Fig. 3E, F). Moreover, we now provide evidence that, upon pharmacological inhibition of the PPP pathway, IACS treatment is cytotoxic for OHT-primed FL^{MycER} cells even at a higher glucose concentration (Suppl. Fig. 3G), which would normally prevent IACS-induced cell death (Donati *et al.*, 2022). The importance of glucose-dependent glutathione regeneration through the PPP for the selective activity of IACS is underscored by the results obtained upon total inhibition of glucose metabolism with 2DG: treating the cells with 2DG induces a cytotoxic response of IACS irrespective of previous MycER activation (Fig. 3G, Suppl. Fig. 3G). This result is most compatible with an unavoidable energy crisis due to concurrent suppression of OxPhos and glycolysis.

Referee #3 (Comments on Novelty/Model System for Author):

Note to editor: this is a rather fundamental paper, some aspects require more rigorous data, and the direct medical impact cannot be predicted as yet (impossible question in my opinion)

This is an important point, and we thank the Referee for bringing it up. Our study provides an innovative mechanism-based therapeutic concept, with pre-clinical proof-of-principle: whether this may ultimately have a direct impact in patients may only be assessed in tailored clinical studies, as generally true for this type of studies.

Referee #3 (Remarks for Author):

Donati et al employ an inducible system for expresion of Myc in a B cell line to investigate the synthetic lethality between Myc overexpression and inhibition of ETC complex I by IACS-010759 - a compound currently in clincial trials.
They report that MYC hyperactivation and ETC inhibition disrupt redox homeostasis, leading to oxidative stress and apoptosis. By combining IACS-010759 with pro-oxidant drugs such as ascorbate, the efficacy can be further enhanced.
The work is a direct extension of a related recent paper by the group (Donati et al Mol Oncol 2022) where Myc overexpression combined with Bcl2 inhibition were investigated. The current work complements the previous paper by showing that Myc makes cells vulnerable for oxidative stress, which is in itself not a novel observation. The authors do provide novel mechanistic insight in order to make this paper novel and relevant. In addition, though all mechanistic experiments are done with just 1 genetically manipulated cell line, authors do make the transition to additional B cell lines and patient derived PDX in Figure 6, which is commendable.

While it is true that all the mechanistic experiments were done in the FL^{MycER} model, a mouse B-cell line engineered for conditional super-activation MYC, we would like to point out that we present important confirmatory results in two MYC-rearranged lymphoma cell lines, DoHH2 and Ramos. In particular, those data reinforce the conclusion that the anti-tumoral activity of IACS-010759 is potentiated by hampering antioxidant defenses (Suppl. Fig. 2A and 3H; see also the reply to Referee 2). Moreover, we have added a dose response curve showing ascorbate-dependent potentiation of cell killing by IACS-010759 in BaF^{MycER} (Fig. 4A).

Nevertheless several aspects should be improved and/or clarified to make the article more coherent and impactful.

Major comments

1. Figure 1B: This essential WB data require quantification of multiple experiments, as the change in Nrf2 levels in the nucleus is not clear. Why does OHT+IACS not result in increased Nrf2, if the signature of Nrf2 is present regardless of IACS+/-? In addition protein markers/ kD should be indicated, especially as there is controversy on the size of Nrf2 by western blot.

> As discussed above, a number of control experiments were performed, as a result of which we finally decided to remove the Nrf2 blot. Please refer to our reply to Referee #1 (point 4) for a detailed explanation.

2. Figure 2 E: in the text is mentioned there is an abrupt increase of 405/488. If t0 is defined per each cell, then many of them have this abrupt increase much before (2h) they die. Can these differences between cells be explained/discussed?

> As originally written in our text, our time-lapse data showed that "an abrupt fall in GSH availability (as revealed by the increase in 405/488 nm fluorescence ratio) regularly preceded death in double-treated cells (Figure 2E, Movie S1)." We believe this conclusion to be appropriate, accounting for the fact that other biological parameters, which are stochastic by nature, are likely to determine the onset of apoptosis following the drop in GSH. We have added a new sentence following to account for this phenomenon: "The observed time-window between the drop in GSH and cell death was variable, ranging from few minutes to hours: this should be considered the product of a series of stochastic parameters etc…"

3. Figure 4: Which kind of ROS does ascorbate generate? Are IACS and ascorbate inducing two ROS generating pathways, or do they generate the same kind of ROS species but at higher levels? It would be informative to perfom H2O2 and superoxide measurements as in Fig. 1 C and 2 with the 2 compounds + combination.

> As suggested by the Referee, we tested the effects of ascorbate treatment, alone and in combination with IACS, on $O2^{\bullet -}$ and H_2O_2 production. Our results showed that:
>
> i. "…ascorbate treatment rapidly induced high levels of H_2O_2 in both the cytoplasm and mitochondria, with IACS-010759 co-treatment further enhancing this effect in the cytoplasm (Supplemental Figure 4A)";
> ii. "… somewhat counterintuitive, ascorbate blunted superoxide production in IACS-010759-treated cells (Supplemental Figure 4B), which seem at odds with its pro-oxidant effects. A possible explanation for this result could be that superoxide is being scavenged by ascorbate radicals (Nishikimi, 1975; Scarpa *et al*, 1983) at a rate similar to that achieved with dihydroethidium (Zhao *et al*, 2003), the fluorescent probe used for superoxide quantification".

4. The role of glucose in the culture medium is important in determining the effects of IACS - as presented in the previous paper. Authors should make more clear why and what is new here in relation to glucose. The current article should be an independent body of work; proposed is to show new data on the role of glucose concentration in media and subsequent effects of IACS, ascorbate etc. - see also next point.

In our previous study, we showed that selective killing by IACS of OHT treated FL^{MycER} cells occurred only in reduced glucose medium, but was not associated with disruption of energy homeostasis (Donati *et al.*, 2022). These findings are summarized in our Results, within the first paragraph of the section entitled "Glucose and the pentose phosphate pathway maintain redox homeostasis in IACS-010759 treated cells":

"In all cell types examined so far, including OHT-treated FL^{MycER} cells, the cytotoxic action of IACS-010759 was suppressed by excess glucose in the culture medium […] We previously reported that IACS-010759-induced cell death in OHT-primed cells was not associated with ATP reduction and energy impairment in OHT-primed cells (Donati *et al.*, 2022). Altogether, these observations imply a distinct metabolic requirement for glucose – other than sustaining glycolysis for ATP production – in blocking the cytotoxic action of IACS-010759."

The present study complements and extends those findings by revealing that IACS-induced cell death is associated with disruption of redox homeostasis, an effect that high glucose opposes by boosting NADPH production through the PPP pathway. This is extensively documented in the same section of our Results, ending with the following conclusion: "Altogether, the above data show that glucose protects MYC-overexpressing cells from IACS-010759-induced killing by sustaining NAPDH production through the oxidative phase of the PPP (Figure 3A), ensuring the regeneration of GSH required to maintain redox homeostasis."

Regarding the combination of IACS and ascorbate, the synergism between the two drugs is unaffected by glucose concentration (see Fig. 4C and Suppl. Fig. 4E) due to the fast kinetic of oxidative damage seen with the combination (see Fig. 4B and below the answer to point 5).

5. Since ferric iron chelator deferoxamine (DFX) fully prevented cell death in double-treated cells (Figure 4C), the question arise to what extent the processes studied may involve a ferroptotic component. IS there synergy with ferroptosis inducers/prevention by ferroptosis inhibitors? What is the role of GPX4? In Discussion authors make some assumptions about that, suggesting future investigations, but these aspects should be included here, to increase significance and impact.

The point raised by the Referee regarding the potential involvement of ferroptosis in the response to the IACS/ascorbate combination, and more generally targeting oncogenic MYC-expressing cells with ferroptosis inducers, is indeed of high interest. We have now added data showing that ascorbate treatment was sufficient to induce lipid peroxidation (Fig. 4D). As written in our Results: "given the importance of iron in the combinatorial effects of IACS-010759 and ascorbate, we investigated the involvement of ferroptosis, a form of regulated cell death initiated in response to lipid peroxidation by iron-generated ROS (Jiang *et al*, 2021). Indeed, ascorbate treatment caused a marked increase in lipid peroxidation (Figure 4D); while IACS-010759 had no effect alone, it showed a tendency (albeit below statistical significance) to reinforce the effect of ascorbate. Altogether, the above results suggests that the potentiation of IACS-010759-induced cell death by ascorbate was contributed by ferroptosis."

And in our Discussion: "Remarkably, IACS-010759 and ascorbate synergized in killing MYC-overexpressing B-cells, owing most likely to the cooperative induction of oxidative damage, including lipid peroxidation and ferroptosis."

To address the role of GPX4, as requested by the Referee, we tested RSL3, an inhibitor of the lipid peroxide scavenging enzyme GPX4 (Yang *et al*, 2014). However, unlike ascorbate, RSL3 alone showed strong toxicity in FL^{MycER} cells, regardless of OHT or IACS-010759. While showing that GPX4 is required for cell survival in normal growth conditions, this result does not contribute any

additional insight regarding its contribution to cell killing by IACS-010759 and ascorbate. We thus decided not to include this experiment in our paper.

Minor comments

1. Fig 1A Which part of these data is new and which was already published? In the previous paper Donati, 2022 also RNAseq analysis of OHT-treated versus not-treated are shown but a diferent time point. In there only the 1st 3 pathways are shown and Nrf2 does not appear. Please explain.

The RNA-seq data used here are the same as in our previous study (Donati *et al.*, 2022), thus reflecting exactly the same samples and time-points. However, the analysis presented here is different from that in our previous study, which we have now made clearer in our text (see the first paragraph of the Results, the legend to Fig. 1A and Materials and Methods). Briefly here, in our previous paper we employed Gene set enrichment analysis to compare the differentially expressed genes (DEG) from our samples to signatures from the MSigDB Hallmark collection (Liberzon *et al*, 2015), which does not contain a Nrf2 target signature. Instead, Fig. 1A of the present manuscript shows the result of the analysis performed with the Qiagen Ingenuity Pathway Analysis (IPA) software, which uses its own collection of curated signatures, among which that of the Nrf2 pathway.

2. Fig3C graph appears to be missing, only one representative histogram is shown.

This figure plots the 405/488 nm ratio from each single event (cell), which due to the bimodal value distribution from OHT/IACS treated samples we deemed more informative than a graph plotting the average values.

3. The synergy between Myc, IACS and ascorbate in Figures 5 and S4; these figures are of too low resolution.

Indeed, we realized after submission that the integration in the word file downgraded the resolution of the figures, which we now provide at the original resolution (note that Figure S4 has become S5).

4. Figure 6; Y-axes in B, D do not have a legend.

We have modified the figures to include a legend.

Citations:

Bottger F, Valles-Marti A, Cahn L, Jimenez CR (2021) High-dose intravenous vitamin C, a promising multi-targeting agent in the treatment of cancer. *J Exp Clin Cancer Res* 40: 343

Caro P, Kishan AU, Norberg E, Stanley IA, Chapuy B, Ficarro SB, Polak K, Tondera D, Gounarides J, Yin H *et al* (2012) Metabolic signatures uncover distinct targets in molecular subsets of diffuse large B cell lymphoma. *Cancer Cell* 22: 547-560

Caro-Maldonado A, Wang R, Nichols AG, Kuraoka M, Milasta S, Sun LD, Gavin AL, Abel ED, Kelsoe G, Green DR *et al* (2014) Metabolic reprogramming is required for antibody production that is suppressed in anergic but exaggerated in chronically BAFF-exposed B cells. *J Immunol* 192: 3626-3636

Chen Q, Espey MG, Sun AY, Pooput C, Kirk KL, Krishna MC, Khosh DB, Drisko J, Levine M (2008) Pharmacologic doses of ascorbate act as a prooxidant and decrease growth of aggressive tumor xenografts in mice. *Proc Natl Acad Sci U S A* 105: 11105-11109

D'Andrea A, Gritti I, Nicoli P, Giorgio M, Doni M, Conti A, Bianchi V, Casoli L, Sabo A, Mironov A *et al* (2016) The mitochondrial translation machinery as a therapeutic target in Myc-driven lymphomas. *Oncotarget* 7: 72415-72430

Donati G, Ravà M, Filipuzzi M, Nicoli P, Cassina L, Verrecchia A, Doni M, Rodighiero S, Parodi F, Boletta A *et al* (2022) Targeting mitochondrial respiration and the BCL2 family in high-grade MYC-associated B-cell lymphoma. *Mol Oncol* 16: 1132-1152

Ghandi M, Huang FW, Jane-Valbuena J, Kryukov GV, Lo CC, McDonald ER, 3rd, Barretina J, Gelfand ET, Bielski CM, Li H *et al* (2019) Next-generation characterization of the Cancer Cell Line Encyclopedia. *Nature* 569: 503-508

Holze C, Michaudel C, Mackowiak C, Haas DA, Benda C, Hubel P, Pennemann FL, Schnepf D, Wettmarshausen J, Braun M *et al* (2018) Oxeiptosis, a ROS-induced caspase-independent apoptosis-like cell-death pathway. *Nat Immunol* 19: 130-140

Jiang X, Stockwell BR, Conrad M (2021) Ferroptosis: mechanisms, biology and role in disease. *Nat Rev Mol Cell Biol* 22: 266-282

Kopacz A, Kloska D, Forman HJ, Jozkowicz A, Grochot-Przeczek A (2020) Beyond repression of Nrf2: An update on Keap1. *Free Radic Biol Med* 157: 63-74

Li F, Wang Y, Zeller KI, Potter JJ, Wonsey DR, O'Donnell KA, Kim JW, Yustein JT, Lee LA, Dang CV (2005) Myc stimulates nuclearly encoded mitochondrial genes and mitochondrial biogenesis. *Mol Cell Biol* 25: 6225-6234

Liberzon A, Birger C, Thorvaldsdottir H, Ghandi M, Mesirov JP, Tamayo P (2015) The Molecular Signatures Database (MSigDB) hallmark gene set collection. *Cell Syst* 1: 417-425

Magri A, Germano G, Lorenzato A, Lamba S, Chila R, Montone M, Amodio V, Ceruti T, Sassi F, Arena S *et al* (2020) High-dose vitamin C enhances cancer immunotherapy. *Sci Transl Med* 12

Molina JR, Sun YT, Protopopova M, Gera S, Bandi M, Bristow C, McAfoos T, Morlacchi P, Ackroyd J, Agip ANA *et al* (2018) An inhibitor of oxidative phosphorylation exploits cancer vulnerability. *Nat Med* 24: 1036-1046

Morrish F, Hockenbery D (2014) MYC and mitochondrial biogenesis. *Cold Spring Harb Perspect Med* 4

Nishikimi M (1975) Oxidation of ascorbic acid with superoxide anion generated by the xanthine-xanthine oxidase system. *Biochem Biophys Res Commun* 63: 463-468

Oran AR, Adams CM, Zhang XY, Gennaro VJ, Pfeiffer HK, Mellert HS, Seidel HE, Mascioli K, Kaplan J, Gaballa MR *et al* (2016) Multi-focal control of mitochondrial gene expression by oncogenic MYC provides potential therapeutic targets in cancer. *Oncotarget* 7: 72395-72414

Ravà M, D'Andrea A, Nicoli P, Gritti I, Donati G, Doni M, Giorgio M, Olivero D, Amati B (2018) Therapeutic synergy between tigecycline and venetoclax in a preclinical model of MYC/BCL2 double-hit B cell lymphoma. *Sci Transl Med* 10

Scarpa M, Stevanato R, Viglino P, Rigo A (1983) Superoxide ion as active intermediate in the autoxidation of ascorbate by molecular oxygen. Effect of superoxide dismutase. *J Biol Chem* 258: 6695-6697

Townsend EC, Murakami MA, Christodoulou A, Christie AL, Koster J, DeSouza TA, Morgan EA, Kallgren SP, Liu H, Wu SC *et al* (2016) The Public Repository of Xenografts Enables Discovery and Randomized Phase II-like Trials in Mice. *Cancer Cell* 29: 574-586

Wheeler ML, Defranco AL (2012) Prolonged production of reactive oxygen species in response to B cell receptor stimulation promotes B cell activation and proliferation. *J Immunol* 189: 4405-4416

Wolpaw AJ, Dang CV (2018) MYC-induced metabolic stress and tumorigenesis. *Biochim Biophys Acta*

Yang WS, SriRamaratnam R, Welsch ME, Shimada K, Skouta R, Viswanathan VS, Cheah JH, Clemons PA, Shamji AF, Clish CB *et al* (2014) Regulation of ferroptotic cancer cell death by GPX4. *Cell* 156: 317-331

Zhao H, Kalivendi S, Zhang H, Joseph J, Nithipatikom K, Vasquez-Vivar J, Kalyanaraman B (2003) Superoxide reacts with hydroethidine but forms a fluorescent product that is distinctly different from ethidium: potential implications in intracellular fluorescence detection of superoxide. *Free Radic Biol Med* 34: 1359-1368

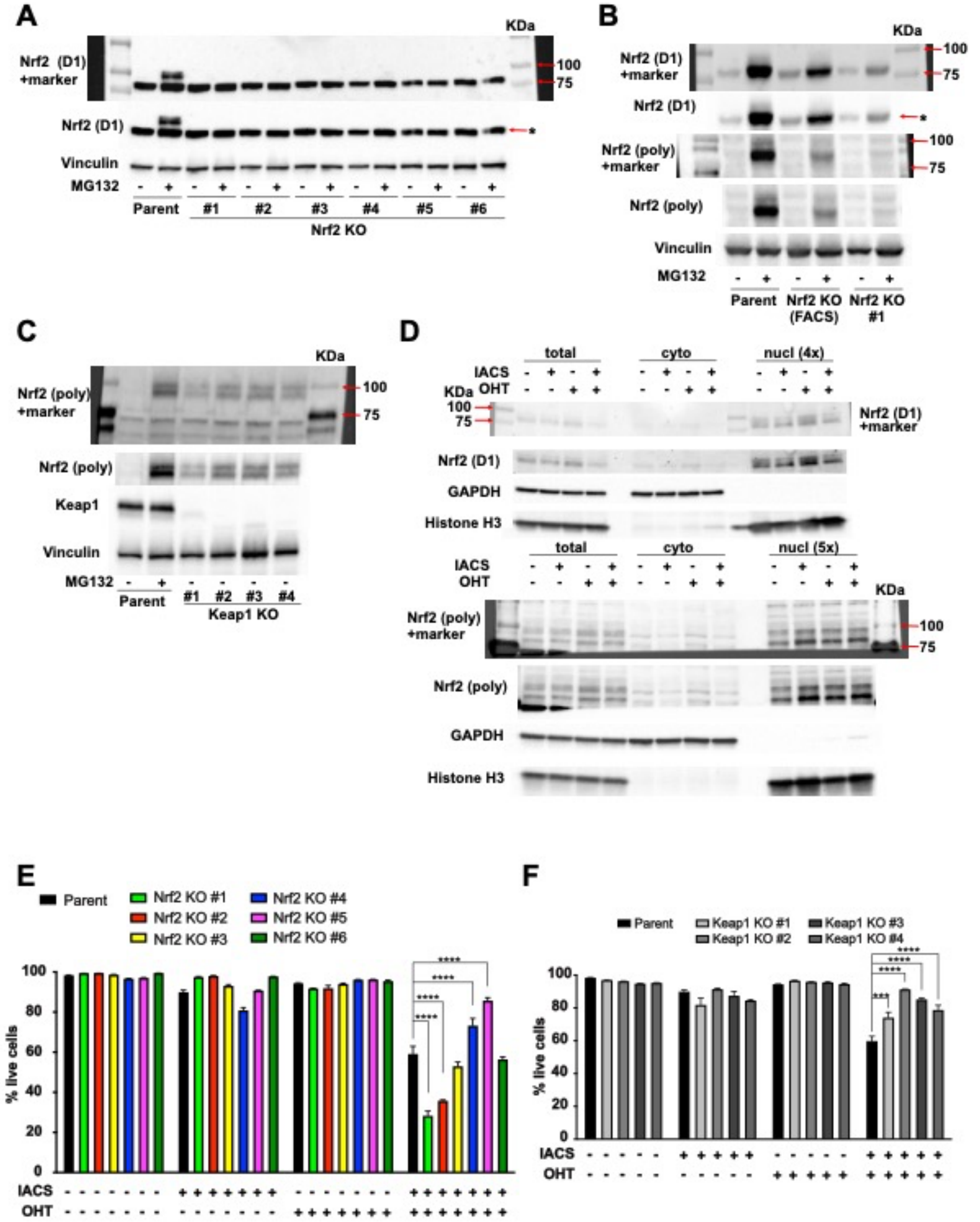

Figure R1. (**A**) Immunoblot on lysates from parental FL^{MycER} cells (Parent) and Nrf2 KO clones (#1-6) treated or not with 10 µM MG132 for 3 hours (MG132 +/-). Nrf2 (D1): blot probed with the anti-Nrf2 monoclonal antibody D1Z9C; +marker: image merged with that of the prestained molecular weight marker on the membrane; * non-specific band. (**B**) Immunoblot on lysates from parental FL^{MycER} cells, NRF KO clone #1, as well as a polyclonal population of Nrf2-targeted cells grown after transfection and flow-cytometric sorting (Nrf2 KO – FACS). Nrf2 (poly): blot probed with the anti-Nrf2 polyclonal antibody (PA5-27882, ThermoFisher). (**C**) Immunoblot on lysates from parental FL^{MycER} cells and Keap1 KO clones (#1-4). (**D**) Immunoblots on total, cytoplasmic and nuclear fractions of FL^{MycER} cells treated as indicated. The top blots correspond to Figure 1B in our original manuscript. The bottom is a repeat of the same experiment with the anti-Nrf2 polyclonal antibody. (**E**) Viability of parental FL^{MycER} cells and Nrf2 KO clones (#1-6) primed with 100 nM OHT and treated with 135 nM IACS for 48 hours, as indicated. (**F**) As in (E) for Keap1 KO clones (#1-4). *** $p \leq 0.001$; **** $p \leq 0.0001$.

4th Apr 2023

Dear Dr. Amati,

Thank you for the submission of your revised manuscript to EMBO Molecular Medicine. I am pleased to inform you that we will be able to accept your manuscript pending the following final amendments:

1) Please address the referee's #3 minor suggestions.
2) Figures: Please upload high-resolution main Figure files and remove figure legends from the files. Figure legends should be placed at the end of the main manuscript file. Please check "Author Guidelines" for more information:
https://www.embopress.org/page/journal/17574684/authorguide#figureformat
3) In the main manuscript file, please do the following:
- Correct/answer the track changes suggested by our data editors by working from the attached document.
- Add up to 5 keywords.
- In M&M, provide the antibody dilutions that were used for each antibody.
- Please rename "Competing Interest" to "Disclosure Statement & Competing Interests". We updated our journal's competing interests policy in January 2022 and request authors to consider both actual and perceived competing interests. Please review the policy https://www.embopress.org/competing-interests and update your competing interests if necessary.
- Author contributions: Please specify author contributions in our submission system. CRediT has replaced the traditional author contributions section because it offers a systematic machine-readable author contributions format that allows for more effective research assessment. You are encouraged to use the free text boxes beneath each contributing author's name to add specific details on the author's contribution. More information is available in our guide to authors:
https://www.embopress.org/page/journal/17574684/authorguide#authorshipguidelines
- In data availability statement please remove information of previous published datasets. If no data produced within this study are deposited in public repositories, please add the sentence: "This study includes no data deposited in external repositories". Please check "Author Guidelines" for more information.
https://www.embopress.org/page/journal/17574684/authorguide#availabilityofpublishedmaterial
4) Supplemental information: Please rename the file to "Appendix" and add table of content on the first page. Rename figures to Appendix Figure S1 etc. and update the callouts in the main manuscript text.
5) Movie: Please rename the movie file to Movie EV1, zipp the legend with the movie file and update the callout in the main text.
6) Funding: Please place the information about funding to "Acknowledgments".
7) The Paper Explained: Please provide a summary of the study structured as followed: PROBLEM - the medical issue you are addressing, RESULTS - the results obtained, and IMPACT - their clinical impact. Please refer to any of our published primary research articles for an example. Please check "Author Guidelines" for more information.
https://www.embopress.org/page/journal/17574684/authorguide#researcharticleguide
8) Synopsis: Every published paper now includes a 'Synopsis' to further enhance discoverability. Synopses are displayed on the journal webpage and are freely accessible to all readers. They include separate synopsis image and synopsis text.
- Synopsis image: Please provide a striking image or visual abstract as a high-resolution jpeg file 550 px-wide x (250-400)-px high to illustrate your article.
- Synopsis text: Please provide a short standfirst (maximum of 300 characters, including space) as well as 2-5 one sentence bullet points that summarise the paper as a .doc file. Please write the bullet points to summarise the key NEW findings. They should be designed to be complementary to the abstract - i.e. not repeat the same text. We encourage inclusion of key acronyms and quantitative information (maximum of 30 words / bullet point). Please use the passive voice.
- Please check your synopsis text and image before submission with your revised manuscript. Please be aware that in the proof stage minor corrections only are allowed (e.g., typos).
9) For more information: This space should be used to list relevant web links for further consultation by our readers. Could you identify some relevant ones and provide such information as well? Some examples are patient associations, relevant databases, OMIM/proteins/genes links, author's websites, etc...
10) Source data: Please upload source data as clearly labelled one (zipped) file per figure.
11) As part of the EMBO Publications transparent editorial process initiative (see our Editorial at http://embomolmed.embopress.org/content/2/9/329), EMBO Molecular Medicine will publish online a Review Process File (RPF) to accompany accepted manuscripts. This file will be published in conjunction with your paper and will include the anonymous referee reports, your point-by-point response and all pertinent correspondence relating to the manuscript. Let us know whether you agree with the publication of the RPF and as here, if you want to remove or not any figures from it prior to publication. Please note that the Authors checklist will be published at the end of the RPF.
12) Please provide a point-by-point letter INCLUDING my comments as well as the reviewer's reports and your detailed responses (as Word file).

I look forward to reading a new revised version of your manuscript as soon as possible.

Yours sincerely,

Zeljko Durdevic

Zeljko Durdevic
Editor
EMBO Molecular Medicine

*** Instructions to submit your revised manuscript ***

*** PLEASE NOTE *** As part of the EMBO Publications transparent editorial process initiative (see our Editorial at https://www.embopress.org/doi/pdf/10.1002/emmm.201000094), EMBO Molecular Medicine will publish online a Review Process File to accompany accepted manuscripts.

In the event of acceptance, this file will be published in conjunction with your paper and will include the anonymous referee reports, your point-by-point response and all pertinent correspondence relating to the manuscript. If you do NOT want this file to be published, please inform the editorial office at contact@embomolmed.org.

When submitting your revised manuscript, please include:

1) a .docx formatted version of the manuscript text (including Figure legends and tables)

2) Separate figure files*

3) supplemental information as Expanded View and/or Appendix. Please carefully check the authors guidelines for formatting Expanded view and Appendix figures and tables at https://www.embopress.org/page/journal/17574684/authorguide#expandedview

4) a letter INCLUDING the reviewer's reports and your detailed responses to their comments (as Word file).

5) The paper explained: EMBO Molecular Medicine articles are accompanied by a summary of the articles to emphasize the major findings in the paper and their medical implications for the non-specialist reader. Please provide a draft summary of your article highlighting
- the medical issue you are addressing,
- the results obtained and
- their clinical impact.
This may be edited to ensure that readers understand the significance and context of the research.
Please refer to any of our published articles for an example.

6) For more information: There is space at the end of each article to list relevant web links for further consultation by our readers. Could you identify some relevant ones and provide such information as well? Some examples are patient associations, relevant databases, OMIM/proteins/genes links, author's websites, etc...

7) Author contributions: the contribution of every author must be detailed in a separate section.

8) EMBO Molecular Medicine now requires a complete author checklist (https://www.embopress.org/page/journal/17574684/authorguide) to be submitted with all revised manuscripts. Please use the checklist as guideline for the sort of information we need WITHIN the manuscript. The checklist should only be filled with page numbers were the information can be found. This is particularly important for animal reporting, antibody dilutions (missing) and exact values and n that should be indicted instead of a range.

9) Every published paper now includes a 'Synopsis' to further enhance discoverability. Synopses are displayed on the journal webpage and are freely accessible to all readers. They include a short stand first (maximum of 300 characters, including space) as well as 2-5 one sentence bullet points that summarise the paper. Please write the bullet points to summarise the key NEW findings. They should be designed to be complementary to the abstract - i.e. not repeat the same text. We encourage inclusion

of key acronyms and quantitative information (maximum of 30 words / bullet point). Please use the passive voice. Please attach these in a separate file or send them by email, we will incorporate them accordingly.

You are also welcome to suggest a striking image or visual abstract to illustrate your article. If you do please provide a jpeg file 550 px-wide x 400-px high.

10) A Conflict of Interest statement should be provided in the main text

11) Please note that we now mandate that all corresponding authors list an ORCID digital identifier. This takes <90 seconds to complete. We encourage all authors to supply an ORCID identifier, which will be linked to their name for unambiguous name identification.

Currently, our records indicate that the ORCID for your account is 0000-0002-2958-1799.

Please click the link below to modify this ORCID:
Link Not Available

12) The system will prompt you to fill in your funding and payment information. This will allow Wiley to send you a quote for the article processing charge (APC) in case of acceptance. This quote takes into account any reduction or fee waivers that you may be eligible for. Authors do not need to pay any fees before their manuscript is accepted and transferred to our publisher.

*Additional important information regarding Figures

Each figure should be given in a separate file and should have the following resolution:
Graphs 800-1,200 DPI
Photos 400-800 DPI
Colour (only CMYK) 300-400 DPI"

Figures are not edited by the production team. All lettering should be the same size and style; figure panels should be indicated by capital letters (A, B, C etc). Gridlines are not allowed except for log plots. Figures should be numbered in the order of their appearance in the text with Arabic numerals. Each Figure must have a separate legend and a caption is needed for each panel.

*Additional important information regarding figures and illustrations can be found at https://bit.ly/EMBOPressFigurePreparationGuideline. See also figure legend preparation guidelines: https://www.embopress.org/page/journal/17574684/authorguide#figureformat

The system will prompt you to fill in your funding and payment information. This will allow Wiley to send you a quote for the article processing charge (APC) in case of acceptance. This quote takes into account any reduction or fee waivers that you may be eligible for. Authors do not need to pay any fees before their manuscript is accepted and transferred to our publisher.

***** Reviewer's comments *****

Referee #1 (Comments on Novelty/Model System for Author):

Developing new treatment strategies against MYC-driven cancers is a pressing need. These cancers are typically aggressive and refractory to first-line therapies, .e.g. the case of double-hit lymphomas. To solve this problem, Donati et al turn their attention to metabolic vulnerabilities imposed by MYC deregulation in cancer cells, which they show can be exploited for therapy. Although the data presented in this manuscript relies on work on a limited number of mouse and human cell lines, as well as xenograft studies in a couple of PDX lines, the thorough molecular studies presented here make a very strong case for specific drug combinations that could be easily moved into preclinical and early phase clinical trials.

Referee #1 (Remarks for Author):

The revised version of this manuscript by Donati et al. has been strengthened by thorough discussions and additional experimental evidence. The old and new data extend previous findings (Donati et al, Mol Oncol 2022; 16) and thoroughly investigate the molecular mechanisms underlying the sensitivity of cell lines with MYC deregulation to a combination of IACS-010759, a specific inhibitor of the electron transport chain (ETC) complex I, and ascorbate or inhibitors of NADPH production through the PPP. The data is well supported with studies in different cell lines and xenograft-PDX lines, as well as a combination of pharmacological and also gene KO experiments to confirm the specificity of the drug effects. These studies make a strong case for using this or similar drug combinations in future clinical studies; and provide a wealth of data and mechanistic insights that should help rationalize this drug combination or additional combinations, and further refine these for future clinical testing.

I do not have major comments to the new version. All my prior concerns were answered, and the new experimental data

included in this revised version of the manuscript - i.e., the use of additional cell lines and the investigation into Nrf2, as well as the detailed discussions in the rebuttal and the main text, are greatly appreciated.

Referee #2 (Comments on Novelty/Model System for Author):

The authors have addressed my concerns.

Referee #2 (Remarks for Author):

Thank you for revisions. The manuscript is clearly enhanced.

Referee #3 (Remarks for Author):

Th eauthors did a thorough job of adressong my (and the other reviewers) comments. There are two minor (text) issues remaining that may be clarified:
1.- In using the 13-C glucose tracing authors used [1,2-13C]-glucose, to discriminate between straight glycolysis and the bypass via the PPP. This could be clarified in the text a bit better (page7/8), especially since the data in fact do not quite confirm their first assumption, making this section difficult to understand.
2. Authors have added new data on the possibel contribution of ferroptosis; the data do not allow a straight yes or no but altogether it is now more informative. The last sentence of that section in Results seems in (syntax) error, please correct: Altogether, the above results suggest that the potentiation of IACS-010759-induced cell death by ascorbate was contributed by ferroptosis.

EMM-2022-16910 V3

Donati et al.

Oxidative stress enhances the therapeutic action of a respiratory inhibitor in MYC-driven lymphoma

Authors' Response to the comments on EMM-2022-16910 V2

Editorial queries:

Dear Dr. Amati,

Thank you for the submission of your revised manuscript to EMBO Molecular Medicine. I am pleased to inform you that we will be able to accept your manuscript pending the following final amendments:

1) Please address the referee's #3 minor suggestions.

This was done as requested (see detail below)

2) Figures: Please upload high-resolution main Figure files and remove figure legends from the files. Figure legends should be placed at the end of the main manuscript file. Please check "Author Guidelines" for more information: https://www.embopress.org/page/journal/17574684/authorguide#figureformat

Done.

3) In the main manuscript file, please do the following:
- Correct/answer the track changes suggested by our data editors by working from the attached document.

Done.

- Add up to 5 keywords.

We introduced 5 keywords as instructed.

- In M&M, provide the antibody dilutions that were used for each antibody.

Done.

- Please rename "Competing Interest" to "Disclosure Statement & Competing Interests". We updated our journal's competing interests policy in January 2022 and request authors to consider both actual and perceived competing interests. Please review the policy https://www.embopress.org/competing-interests and update your competing interests if necessary.

Done.

- Author contributions: Please specify author contributions in our submission system. CRediT has replaced the traditional author contributions section because it offers a systematic machine-readable author contributions format that allows for more effective research assessment. You are encouraged to use the free text boxes beneath each contributing author's name to add specific details on the author's contribution. More information is available in our guide to authors:
https://www.embopress.org/page/journal/17574684/authorguide#authorshipguidelines

Done.

- In data availability statement please remove information of previous published datasets. If no data produced within this study are deposited in public repositories, please add the sentence: "This study includes no data deposited in external repositories".
Please check "Author Guidelines" for more information. https://www.embopress.org/page/journal/17574684/authorguide#availabilityofpublishedmaterial

Done: as instructed, we specified that "This study includes no data deposited in external repositories".

4) Supplemental information: Please rename the file to "Appendix" and add table of content on the first page. Rename figures to Appendix Figure S1 etc. and update the callouts in the main manuscript text.

Done.

5) Movie: Please rename the movie file to Movie EV1, zipp the legend with the movie file and update the callout in the main text.

Done.

6) Funding: Please place the information about funding to "Acknowledgments".

Done.

7) The Paper Explained: Please provide a summary of the study structured as followed: PROBLEM - the medical issue you are addressing, RESULTS - the results obtained, and IMPACT - their clinical impact. Please refer to any of our published primary research articles for an example. Please check "Author Guidelines" for more information.
https://www.embopress.org/page/journal/17574684/authorguide#researcharticleguide

Done.

8) Synopsis: Every published paper now includes a 'Synopsis' to further enhance discoverability. Synopses are displayed on the journal webpage and are freely accessible to all readers. They include separate synopsis image and synopsis text.
- Synopsis image: Please provide a striking image or visual abstract as a high-resolution jpeg file 550 px-wide x (250-400)-px high to illustrate your article.
- Synopsis text: Please provide a short standfirst (maximum of 300 characters, including space) as well as 2-5 one sentence bullet points that summarise the paper as a .doc file.

Please write the bullet points to summarise the key NEW findings. They should be designed to be complementary to the abstract - i.e. not repeat the same text. We encourage inclusion of key acronyms and quantitative information (maximum of 30 words / bullet point). Please use the passive voice.
- Please check your synopsis text and image before submission with your revised manuscript. Please be aware that in the proof stage minor corrections only are allowed (e.g., typos).

Done.

9) For more information: This space should be used to list relevant web links for further consultation by our readers. Could you identify some relevant ones and provide such information as well? Some examples are patient associations, relevant databases, OMIM/proteins/genes links, author's websites, etc...

The following is the corresponding authors' institutional website: https://www.research.ieo.it/research-and-technology/principal-investigators/oncogenes-transcription-and-cancer/

10) Source data: Please upload source data as clearly labelled one (zipped) file per figure.

Done.

11) As part of the EMBO Publications transparent editorial process initiative (see our Editorial at http://embomolmed.embopress.org/content/2/9/329), EMBO Molecular Medicine will publish online a Review Process File (RPF) to accompany accepted manuscripts. This file will be published in conjunction with your paper and will include the anonymous referee reports, your point-by-point response and all pertinent correspondence relating to the manuscript. Let us know whether you agree with the publication of the RPF and as here, if you want to remove or not any figures from it prior to publication. Please note that the Authors checklist will be published at the end of the RPF.

We agree with the publication of our detailed response, including the figures.

12) Please provide a point-by-point letter INCLUDING my comments as well as the reviewer's reports and your detailed responses (as Word file).

Provided with the present letter.

***** Reviewer's comments *****

Referee #1 (Comments on Novelty/Model System for Author):

Developing new treatment strategies against MYC-driven cancers is a pressing need. These cancers are typically aggressive and refractory to first-line therapies, .e.g. the case of double-hit lymphomas. To solve this problem, Donati et al turn their attention to metabolic vulnerabilities imposed by MYC deregulation in cancer cells, which they show can be exploited for therapy. Although the data presented in this manuscript relies on work on a limited number of mouse and human cell lines, as well as xenograft studies in a couple of

PDX lines, the thorough molecular studies presented here make a very strong case for specific drug combinations that could be easily moved into preclinical and early phase clinical trials.

Referee #1 (Remarks for Author):

The revised version of this manuscript by Donati et al. has been strengthened by thorough discussions and additional experimental evidence. The old and new data extend previous findings (Donati et al, Mol Oncol 2022; 16) and thoroughly investigate the molecular mechanisms underlying the sensitivity of cell lines with MYC deregulation to a combination of IACS-010759, a specific inhibitor of the electron transport chain (ETC) complex I, and ascorbate or inhibitors of NADPH production through the PPP. The data is well supported with studies in different cell lines and xenograft-PDX lines, as well as a combination of pharmacological and also gene KO experiments to confirm the specificity of the drug effects. These studies make a strong case for using this or similar drug combinations in future clinical studies; and provide a wealth of data and mechanistic insights that should help rationalize this drug combination or additional combinations, and further refine these for future clinical testing.

I do not have major comments to the new version. All my prior concerns were answered, and the new experimental data included in this revised version of the manuscript - i.e., the use of additional cell lines and the investigation into Nrf2, as well as the detailed discussions in the rebuttal and the main text, are greatly appreciated.

Referee #2 (Comments on Novelty/Model System for Author):

The authors have addressed my concerns.

Referee #2 (Remarks for Author):

Thank you for revisions. The manuscript is clearly enhanced.

Referee #3 (Remarks for Author):

Th eauthors did a thorough job of adressong my (and the other reviewers) comments. There are two minor (text) issues remaining that may be clarified:
1.- In using the 13-C glucose tracing authors used [1,2-13C]-glucose, to discriminate between straight glycolysis and the bypass via the PPP. This could be clarified in the text a bit better (page7/8), especially since the data in fact do not quite confirm their first assumption, making this section difficult to understand.

> We have inserted a new paragraph break and explanatory section in our text, which now reads as follows:
>
> “Lactate is the end product of glycolysis: by using the [1,2-^{13}C]glucose tracer, lactate produced by glucose that passed directly through glycolysis can be distinguished from that produced by glucose processed through the PPP (Figure 3A): the former would be quantified as lactate M2 isotopomer, and the latter as lactate M1. Given the decreased

PPP flux in IACS-010759 treated cells, we expected a reduced production of lactate M1: etc…"

2. Authors have added new data on the possibel contribution of ferroptosis; the data do not allow a straight yes or no but altogether it is now more informative. The last sentence of that section in Results seems in (syntax) error, please correct: Altogether, the above results suggest that the potentiation of IACS-010759-induced cell death by ascorbate was contributed by ferroptosis.

We have modified this final sentence, which now reads as follows:

"Altogether, the above results suggest that ferroptosis contributes to the potentiation of IACS-010759-induced cell death by ascorbate."

21st Apr 2023

Dear Dr. Amati,

We are pleased to inform you that your manuscript is accepted for publication and is now being sent to our publisher to be included in the next available issue of EMBO Molecular Medicine.

Please read below for additional IMPORTANT information regarding your article, its publication and the production process.

Congratulations on your interesting work,

Zeljko Durdevic

Zeljko Durdevic
Editor
EMBO Molecular Medicine

Follow us on Twitter @EmboMolMed
Sign up for eTOCs at embopress.org/alertsfeeds

*** *** *** IMPORTANT INFORMATION *** *** ***

SPEED OF PUBLICATION
The journal aims for rapid publication of papers, using using the advance online publication "Early View" to expedite the process: A properly copy-edited and formatted version will be published as "Early View" after the proofs have been corrected. Please help the Editors and publisher avoid delays by providing e-mail address(es), telephone and fax numbers at which author(s) can be contacted.

Should you be planning a Press Release on your article, please get in contact with embomolmed@wiley.com as early as possible, in order to coordinate publication and release dates.

LICENSE AND PAYMENT:

All articles published in EMBO Molecular Medicine are fully open access: immediately and freely available to read, download and share.

EMBO Molecular Medicine charges an article processing charge (APC) to cover the publication costs. You, as the corresponding author for this manuscript, should have already received a quote with the article processing fee separately. Please let us know in case this quote has not been received.

Once your article is at Wiley for editorial production you will receive an email from Wiley's Author Services system, which will ask you to log in and will present you with the publication license form for completion. Within the same system the publication fee can be paid by credit card, an invoice, pro forma invoice or purchase order can be requested.

Payment of the publication charge and the signed Open Access Agreement form must be received before the article can be published online.

PROOFS

You will receive the proofs by e-mail approximately 2 weeks after all relevant files have been sent o our Production Office. Please return them within 48 hours and if there should be any problems, please contact the production office at embopressproduction@wiley.com.

Please inform us if there is likely to be any difficulty in reaching you at the above address at that time. Failure to meet our

deadlines may result in a delay of publication.

All further communications concerning your paper proofs should quote reference number EMM-2022-16910-V3 and be directed to the production office at embopressproduction@wiley.com.

Thank you,

Zeljko Durdevic
Editor
EMBO Molecular Medicine

EMBO Press Author Checklist

Corresponding Author Name: Bruno Amati	
Journal Submitted to: EMBO Molecular Medicine	
Manuscript Number: EMM-2022-16910-V2	

USEFUL LINKS FOR COMPLETING THIS FORM
The EMBO Journal - Author Guidelines
EMBO Reports - Author Guidelines
Molecular Systems Biology - Author Guidelines
EMBO Molecular Medicine - Author Guidelines

Reporting Checklist for Life Science Articles (updated January
This checklist is adapted from Materials Design Analysis Reporting (MDAR) Checklist for Authors. MDAR establishes a minimum set of requirements in transparent reporting in the life sciences (see Statement of Task: 10.31222/osf.io/9sm4x). Please follow the journal's guidelines in preparing your
Please note that a copy of this checklist will be published alongside your article.

Abridged guidelines for figures
1. Data
The data shown in figures should satisfy the following conditions:
- the data were obtained and processed according to the field's best practice and are presented to reflect the results of the experiments in an accurate and unbiased manner.
- ideally, figure panels should include only measurements that are directly comparable to each other and obtained with the same assay.
- plots include clearly labeled error bars for independent experiments and sample sizes. Unless justified, error bars should not be shown for technical
- if n<5, the individual data points from each experiment should be plotted. Any statistical test employed should be justified.
- Source Data should be included to report the data underlying figures according to the guidelines set out in the authorship guidelines on Data

2. Captions
Each figure caption should contain the following information, for each panel where they are relevant:
- a specification of the experimental system investigated (eg cell line, species name).
- the assay(s) and method(s) used to carry out the reported observations and measurements.
- an explicit mention of the biological and chemical entity(ies) that are being measured.
- an explicit mention of the biological and chemical entity(ies) that are altered/varied/perturbed in a controlled manner.
- the exact sample size (n) for each experimental group/condition, given as a number, not a range;
- a description of the sample collection allowing the reader to understand whether the samples represent technical or biological replicates (including how many animals, litters, cultures, etc.).
- a statement of how many times the experiment shown was independently replicated in the laboratory.
- definitions of statistical methods and measures:
  - common tests, such as t-test (please specify whether paired vs. unpaired), simple χ2 tests, Wilcoxon and Mann-Whitney tests, can be unambiguously identified by name only, but more complex techniques should be described in the methods section;
  - are tests one-sided or two-sided?
  - are there adjustments for multiple comparisons?
  - exact statistical test results, e.g., P values = x but not P values < x;
  - definition of 'center values' as median or average;
  - definition of error bars as s.d. or s.e.m.

Please complete ALL of the questions below.
Select "Not Applicable" only when the requested information is not relevant for your study.

Materials

Newly Created Materials	Information included in the manuscript?	In which section is the information available? (Reagents and Tools Table, Materials and Methods, Figures, Data Availability Section)
New materials and reagents need to be available; do any restrictions apply?	Yes	Engineered KO derivatives of the FL5.12 cell line and DNA constructs are described in Results and Materials and Methods section.

Antibodies	Information included in the manuscript?	In which section is the information available? (Reagents and Tools Table, Materials and Methods, Figures, Data Availability Section)
For **antibodies** provide the following information:<br>- Commercial antibodies: RRID (if possible) or supplier name, catalogue number and or/clone number<br>- Non-commercial: RRID or citation	Yes	Materials and Methods section

DNA and RNA sequences	Information included in the manuscript?	In which section is the information available? (Reagents and Tools Table, Materials and Methods, Figures, Data Availability Section)
**Short novel DNA or RNA including primers, probes**: provide the sequences.	Yes	Materials and Methods section

Cell materials	Information included in the manuscript?	In which section is the information available? (Reagents and Tools Table, Materials and Methods, Figures, Data Availability Section)
**Cell lines:** Provide species information, strain. Provide accession number in repository **OR** supplier name, catalog number, clone number, and/**OR** RRID.	Yes	Materials and Methods section
**Primary cultures:** Provide species, strain, sex of origin, genetic modification status.	Not Applicable	
Report if the cell lines were recently **authenticated** (e.g., by STR profiling) and tested for mycoplasma contamination.	Yes	IEO's core Tissue Culture facility authenticates and tests all cell lines for mycoplasma infection.

Experimental animals	Information included in the manuscript?	In which section is the information available? (Reagents and Tools Table, Materials and Methods, Figures, Data Availability Section)
**Laboratory animals or Model organisms:** Provide species, strain, sex, age, genetic modification status. Provide accession number in repository **OR** supplier name, catalog number, clone number, **OR** RRID.	Yes	Materials and Methods section
**Animal observed in or captured from the field:** Provide species, sex, and age where possible.	Not Applicable	
Please detail **housing and husbandry conditions**.	Yes	Experiments involving animals were done in accordance with the Italian Laws (D.lgs. 26/2014), which enforces Dir. 2010/63/EU (Directive

Plants and microbes	Information included in the manuscript?	In which section is the information available? (Reagents and Tools Table, Materials and Methods, Figures, Data Availability Section)
**Plants:** provide species and strain, ecotype and cultivar where relevant, unique accession number if available, and source (including location for collected wild specimens).	Not Applicable	
**Microbes:** provide species and strain, unique accession number if available, and source.	Yes	Standard E.coli laboratory strains were used for recombinant DNA experiments.

Human research participants	Information included in the manuscript?	In which section is the information available? (Reagents and Tools Table, Materials and Methods, Figures, Data Availability Section)
If collected and within the bounds of privacy constraints report on age, sex and gender or ethnicity for all study participants.	Not Applicable	

Core facilities	Information included in the manuscript?	In which section is the information available? (Reagents and Tools Table, Materials and Methods, Figures, Data Availability Section)
If your work benefited from core facilities, was their service mentioned in the acknowledgments section?	Yes	Materials and Methods: All lines were stocked and made available by IEO's core Tissue Culture facility, where they were also validated and tested for mycoplasma infection. Acknowledgments: We thank […] Simona Ronzoni for her help with flow cytometry.

Design

**Study protocol**	**Information included in the manuscript?**	**In which section is the information available?** (Reagents and Tools Table, Materials and Methods, Figures, Data Availability Section)
If study protocol has been **pre-registered, provide DOI in the manuscript**. For clinical trials, provide the trial registration number **OR** cite DOI.	Not Applicable	
Report the **clinical trial registration number** (at ClinicalTrials.gov or equivalent), where applicable.	Not Applicable	

**Laboratory protocol**	**Information included in the manuscript?**	**In which section is the information available?** (Reagents and Tools Table, Materials and Methods, Figures, Data Availability Section)
Provide DOI OR other citation details if **external detailed step-by-step protocols** are available.	Not Applicable	

**Experimental study design and statistics**	**Information included in the manuscript?**	**In which section is the information available?** (Reagents and Tools Table, Materials and Methods, Figures, Data Availability Section)
Include a statement about **sample size** estimate even if no statistical methods were used.	Yes	Materials and Methods section, Xenograft models and treatment.
Were any steps taken to minimize the effects of subjective bias when allocating animals/samples to treatment (e.g. **randomization procedure**)? If yes, have they been described?	Yes	Materials and Methods section, Xenograft models and treatment.
Include a statement about **blinding** even if no blinding was done.	Yes	Materials and Methods section, Quantification and statistical analysis.
Describe **inclusion/exclusion criteria** if samples or animals were excluded from the analysis. Were the criteria pre-established?<br><br>If sample or data points were omitted from analysis, report if this was due to attrition or intentional exclusion and provide justification.	Yes	Materials and Methods section, Xenograft models and treatment.The criteria were pre-established.
For every figure, are **statistical tests** justified as appropriate? Do the data meet the assumptions of the tests (e.g., normal distribution)? Describe any methods used to assess it. Is there an estimate of variation within each group of data? Is the variance similar between the groups that are being statistically compared?	Yes	Materials and Methods section, Quantification and statistical analysis.

**Sample definition and in-laboratory replication**	**Information included in the manuscript?**	**In which section is the information available?** (Reagents and Tools Table, Materials and Methods, Figures, Data Availability Section)
In the figure legends: state number of times the experiment was **replicated** in laboratory.	Yes	Replicate numbers are indicate in the Figure Legends.
In the figure legends: define whether data describe **technical or biological replicates**.	Yes	The nature of the replicates is indicated in the Figure Legends.

Ethics

**Ethics**	**Information included in the manuscript?**	**In which section is the information available?** (Reagents and Tools Table, Materials and Methods, Figures, Data Availability Section)
Studies involving **human participants**: State details of **authority granting ethics approval** (IRB or equivalent committee(s), provide reference number for approval.	Not Applicable	
Studies involving **human participants**: Include a statement confirming that **informed consent** was obtained from all subjects and that the experiments conformed to the principles set out in the WMA Declaration of Helsinki and the Department of Health and Human Services Belmont Report.	Not Applicable	
Studies involving **human participants:** For publication of **patient photos**, include a statement confirming that consent to publish was obtained.	Not Applicable	
Studies involving experimental **animals**: State details of **authority granting ethics approval** (IRB or equivalent committee(s), provide reference number for approval. Include a statement of compliance with ethical regulations.	Yes	AuthorIty: Italian Health Ministry (MINSTERO DELLA SALUTE; DIREZIONE GENERALE DELLA SANITÀ ANIMALE E DEI FARMACI VETERINARI). As defined in our Materials and Methods: Experiments involving animals were done in accordance with the Italian Laws (D.lgs. 26/2014), which enforces Dir. 2010/63/EU (Directive
Studies involving **specimen and field samples:** State if relevant **permits** obtained, provide details of authority approving study; if none were required, explain why.	Not Applicable	

**Dual Use Research of Concern (DURC)**	**Information included in the manuscript?**	**In which section is the information available?** (Reagents and Tools Table, Materials and Methods, Figures, Data Availability Section)
Could your study fall under dual use research restrictions? Please check biosecurity documents and list of **select agents and toxins** (CDC): https://www.selectagents.gov/sat/list.htm	Not Applicable	
If you used a select agent, is the security level of the lab appropriate and reported in the manuscript?	Not Applicable	
If a study is subject to dual use research of concern regulations, is the name of the **authority granting approval and reference number** for the regulatory approval provided in the manuscript?	Not Applicable	

Reporting

The MDAR framework recommends adoption of discipline-specific guidelines, established and endorsed through community initiatives. Journals have their own policy about requiring specific guidelines and recommendations to complement MDAR.

**Adherence to community standards**	**Information included in the manuscript?**	**In which section is the information available?** (Reagents and Tools Table, Materials and Methods, Figures, Data Availability Section)
State if relevant guidelines or checklists (e.g., **ICMJE, MIBBI, ARRIVE, PRISMA**) have been followed or provided.	Not Applicable	
For **tumor marker prognostic studies,** we recommend that you follow the **REMARK** reporting guidelines (see link list at top right). See author guidelines, under 'Reporting Guidelines'. Please confirm you have followed these guidelines.	Not Applicable	
For **phase II and III randomized controlled trials**, please refer to the **CONSORT** flow diagram (see link list at top right) and submit the CONSORT checklist (see link list at top right) with your submission. See author guidelines, under 'Reporting Guidelines'. Please confirm you have submitted this list.	Not Applicable	

Data Availability

**Data availability**	**Information included in the manuscript?**	**In which section is the information available?** (Reagents and Tools Table, Materials and Methods, Figures, Data Availability Section)
Have **primary datasets** been deposited according to the journal's guidelines (see 'Data Deposition' section) and the respective accession numbers provided in the Data Availability Section?	Not Applicable	
Were **human clinical and genomic datasets** deposited in a public access-controlled repository in accordance to ethical obligations to the patients and to the applicable consent agreement?	Not Applicable	
Are **computational models** that are central and integral to a study available without restrictions in a machine-readable form? Were the relevant accession numbers or links provided?	Not Applicable	
If publicly available data were reused, provide the respective **data citations in the reference list**.	Yes	As described in out Materials and Methods section: The RNA-seq data and Differentially expressed genes (DEGs) called in FLMycER cells treated with OHT and/or IACS-010759 were described in our previous work (Donati et
